# Association of Preoperative NANOG-Positive Circulating Tumor Cell Levels With Recurrence of Hepatocellular Carcinoma

**DOI:** 10.3389/fonc.2021.601668

**Published:** 2021-05-27

**Authors:** Yongrong Lei, Xishu Wang, Heng Sun, Yuna Fu, Yichen Tian, Ludi Yang, Jianhua Wang, Feng Xia

**Affiliations:** ^1^ Key Laboratory of Biorheological Science and Technology, Ministry of Education, College of Bioengineering, Chongqing University, Chongqing, China; ^2^ Key Laboratory of Hepatobiliary and Pancreatic Surgery, Institute of Hepatobiliary Surgery, Southwest Hospital, the First Hospital Affiliated to AMU (Southwest Hospital), Chongqing, China

**Keywords:** recurrence, cancer stem cells, hepatocellular carcinoma, circulating tumor cells, epithelial-mesenchymal

## Abstract

**Background:**

Cancer stem cells (CSCs) and Circulating tumor cells (CTCs) have been proposed as fundamental causes for the recurrence of hepatocellular carcinoma (HCC). CTCs isolated from patients with HCC illustrate a unique Nanog expression profile analysis. The aim of this study was to enhance the prediction of recurrence and prognosis of the CTC phenotype in patients with HCC by combining Nanog expression into a combined forecasting model.

**Subjects, Materials, and Methods:**

We collected 320 blood samples from 160 patients with HCC cancer before surgery and used CanPatrol™ CTC enrichment technology and in situ hybridization (ISH) to enrich and detect CTCs and CSCs. Nanog expression in all CTCs was also determined. In addition, RT-PCR and immunohistochemistry were used to study the expression of Nanog, E-Cadherin, and N-Cadherin in liver cancer tissues and to conduct clinical correlation studies.

**Results:**

The numbers of ^EpCAM mRNA+^ CTCs and ^Nanog mRNA+^ CTCs were strongly correlated with postoperative HCC recurrence (CTC number (P = 0.03), the total number of mixed CTCS (P = 0.02), and Nanog> 6.7 (P = 0.001), with Nanog > 6.7 (P = 0.0003, HR = 2.33) being the most crucial marker. There are significant differences in the expression of Nanog on different types of CTC: most Epithelial CTCs do not express Nanog, while most of Mixed CTC and Mesenchymal CTC express Nanog, and their positive rates are 38.7%, 66.7%, and 88.7%, respectively, (P=0.0001). Moreover, both CTC (≤/> 13.3) and Nanog (≤/>6.7) expression were significantly correlated with BCLC stage, vascular invasion, tumor size, and Hbv-DNA (all P < 0.05). In the young group and the old group, patients with higher Nanog expression had a higher recurrence rate. (P < 0.001).

**Conclusions:**

The number of Nanog-positive cells showed positive correlation with the poor prognosis of HCC patients. The detection and analysis of CTC markers (EpCAM and CK8, 18, CD45 Vimentin,Twist and 19) and CSCs markers (NANOG) are of great value in the evaluation of tumor progression.

## Introduction

Tumor invasion and metastasis can cause cancer-related mortality to reach more than 90% of the key reasons (1–3). The latest research shows that CTCs may be present in cell populations that come from either a common invasion of the cancer cell population or fragments shed from the primary tumor into the vascular system (4, 5). CTCs that mobilize from the primary lesion and enter the blood circulation are considered important factors for postoperative recurrence and metastasis in patients with malignant tumors (6–8). There have been studies that can isolate a single CTC in the blood of cancer patients (9). Surgical removal of tumors or (including biopsy) will increase CTCs in the blood system. In many cancers, the higher the content of CTCs in the blood system, the worse the prognosis after surgery (10, 11). and correlate with poor outcomes in patients with inchoate HCC (12–14).

During the EMT process, the invasion and metastasis rate of tumors increases, and the resistance of chemotherapy drugs is improved (15–17). There are phenotype and morphological changes of cancer cells formed by EMT in circulating tumor cells of HCC (18, 19). If the content of CSCs in HCC is high, the rate of tumor recurrence and metastasis will increase, the prognosis will be poor, and chemotherapy resistance will be strong (20). Due to the heterogeneity of tumors, the more tumor stem cells there are, the stronger the tumor metastasis (21).

CTCs may enhance their self-renewal capacity during cancer metastasis (22–25). Tumor cells with a mesenchymal-like phenotype are highly invasion and metastasis, but not all EMT-transformed cells are CSCs. Mesenchymal stem cells exhibit intrinsic therapeutic resistance and maintain their self-renewal ability to form a heterogeneous lineage of tumor cells (26–28). Previous studies have proposed different biomarkers for the self-renewal capabilities of HCC (29). NANOG as a transcriptional regulator is highly expressed in some cancer stem cells. Past research has indicated that Nanog plays an important part in HCC resistance and self-renewal (17, 30). In addition to Nanog, CTC-related CSC biomarkers include CD44 (25, 31), CD34 (32), and CD133 (33). Therefore, double checking of CTCs and CSCs can be an crucial monitoring therapy tool for liver cancer.

In this research, we speculated that CSCs embue CTCs with tumorigenicity and are the underlying initiators of HCC recurrence and metastasis (34). Thus, CSCs might be linked to an adverse clinical outcome. We optimized the CanPatrol ^TM^ CTC analysis system to isolate and classify CTCs based on EMT and Nanog phenotypes (35). Thus, We conducted this study mainly to investigate the relationship between the total number of CTCs in the blood, various phenotypes, nanog (CSCs markers) and postoperative recurrence and prognosis of liver cancer patients (35).

## Material and Methods

### Patient Samples

From March 2012 to December 2019, a total of 160 patients treated with R0 resection at the Southwest Hospital, Chongqing, China, were enrolled. The inclusion criteria were as follows: 1) PST score(performance status test) 0-1 and Child-Pugh A stage; 2) World Health Organization Standards were used for the pathological diagnosis of HCC; 3) Achievement of R0 resection (Thorough elimination of the tumor and negative margins according to the naked eye, and no intrahepatic and extrahepatic metastases were observed); and 4) Without prior antitumor therapy. BCLC stage and Edmondson were used for tumor staging and differentiation, respectively (36). Tumor markers PIVKA (vitamin K deficiency or antagonist-II induced protein) and AFP (alpha-fetoprotein) and related detection factors AST (aspartate aminotransferase) and ALT (alanine aminotransferase) are often used to improve the diagnosis of liver cancer Correct rate (37). The deadline recorded follow-up was July 31, 2019. The research is based on the Helsinki Guidelines Declaration, in which the ethics of the pilot program was derived from the Ethics Committee of the Southwest Hospital. Written informed consent has been obtained from the selected patient.

### Isolation of CTCs With the CanPatrolTM System and Tri-Color RNA-ISH Assay

**Figure 1:**
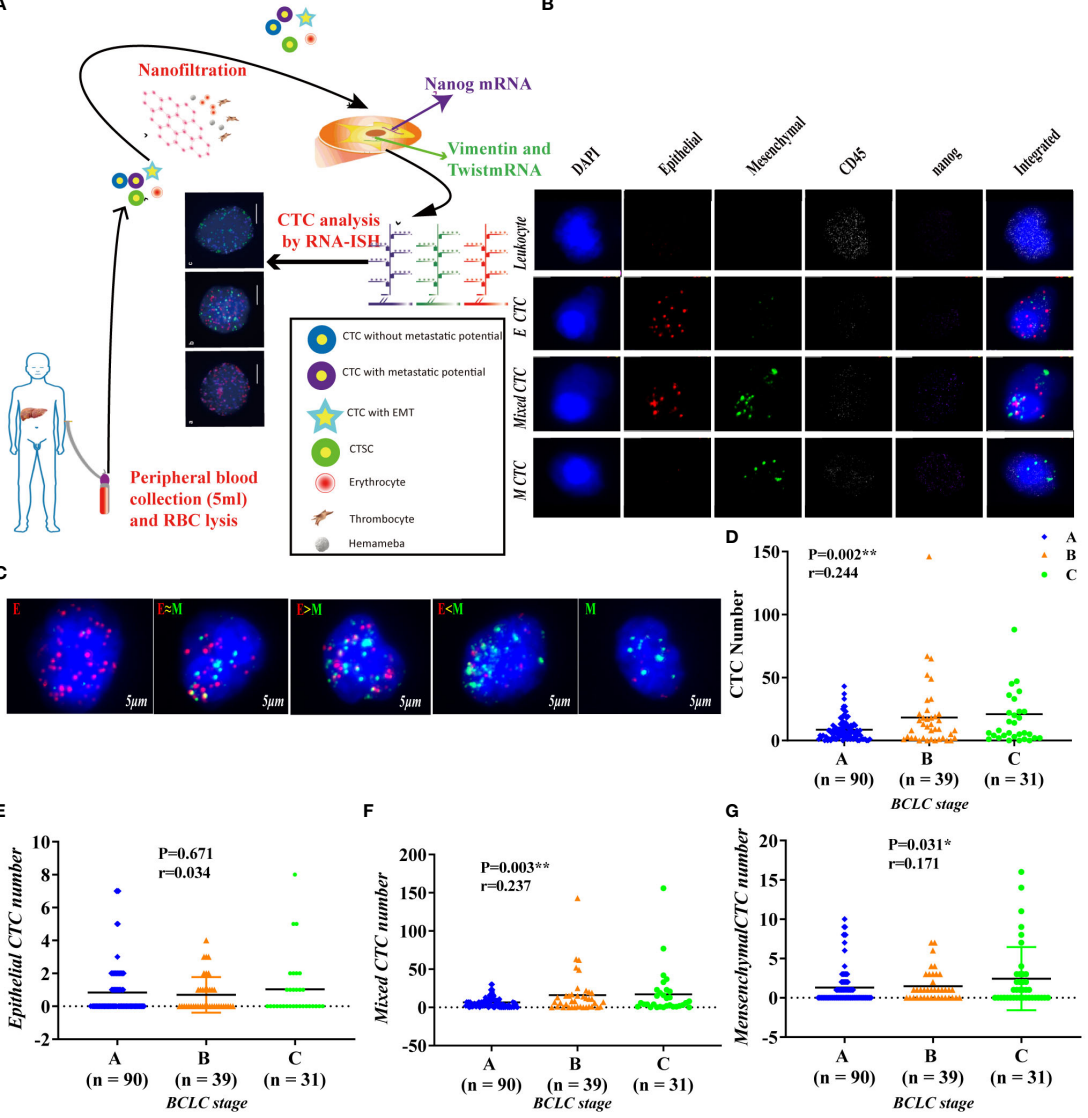
Use RNA-ISH technology to enrich and analyze CTCs in blood samples of HCC patients **(A)** The expression of epithelial marker mRNA (EpCAM and E-cadherin) and **(B)** mesenchymal marker mRNA (Twist and vimentin) and CSC marker mRNA (nanog) were validated by qPCR, p<0.05; **(C)** The protein expression levels of epithelial markers (EpCAM and E-cadherin), p<0.001. **(D–G)** The total number of CTCs, epithelial CTCs, mixed CTCs, and mesenchymal CTCs correlated with BCLC staging, p<0.05. **represents P<0.01, *represents P<0.05.

In order to separate and detect CTCs we used Can -Patrol ^TM^ system (38, 39) (**Figure 1A**). We collected peripheral blood of liver cancer patients 1-3 days before surgery. Collected 15 ml of peripheral blood sample from each patient, placed in EDTA tubes and centrifuged and collected cell beads. The supernatant was discarded, 5 mL PBS was added to the tube, and the cell pellet was resuspended. The high-concentration cell suspension was passed through the filter membrane under vacuum conditions, and CTCs were collected on the filter membrane (37).

We have adopted multiple RNA in situ hybridization (RNA-ISH) technology and designed three sets of nucleic acid probes to detect the expression of CSCs marker (nanog), Leukocyte Marker (CD45), epithelial marker (CK8, 18 and 19 and EpCAM), and mesenchymal marker (Twist and Vimentin) in CTCs (40). We used fluorescence optical microscope to quantitatively analyze the cells, red to mark epithelial cells, green to mark mesenchymal cells, purple to mark Nanog^+^ cells, and white to mark CD45^+^ cells, and used 4,6-diamidino-2-phenylindole (DAPI) to stain Nucleus (37, 41). The primer sequences used by the probes are shown in **Table S2**.

### Immunohistochemistry

To assess the relationship of Nanog expression in human HCC, we retrospectively studied Nanog expression in resected tissues from HCC patients. We collected 160 paraffin-embedded hepatocellular carcinoma specimens. The sections were then incubated with anti-N-cadherin (Proteintech, 22018-1-AP, 1:1000) and anti-Nanog (Abcam, ab80892, 1:200) antibodies at 4°C overnight. We used DAKO En-Vision (K5007) for immunohistochemical analysis, and two pathologists from Southwest Hospital independently completed the pathological assessment of each patient's tissue specimen. The final score of the immune response = the percentage of stained area × the intensity of staining, the staining area score method: 75-100% = 4, 50-74% = 3, 25-49% = 2; staining intensity score: high staining = 3, moderate staining =2, weak staining=1, negative=0, the final score range of staining is 0-12 (42). Follow-up information of patients was collected. The mean follow-up time was 28.5 months, and the longest was 71 months (43, 44).

### Statistical Methods

SPSS 20.0 was used for all statistical analyses. Pearson, Spearman and Kendall's tau-b correlations were used for correlation analysis. We used the χ2 test and t test to evaluate categorical data and measurement data respectively (35). To evaluate the prognostic factors of early recurrence, univariate analysis and multivariate Cox regression analysis were used. Kaplan-Meier survival analysis was used to assess the association between recurrence and prognostic factors, and log-rank tests were used to evaluate the differences between curves. A P value<0.05 was considered statistically significant.

## Results

### Patient Characteristics

Among the patients included in the clinical study, 160 patients with HCC provided a total of 320 blood samples. The characteristics of the study participants are listed in Additional file 1. .A total of 160 HCC patients (141 males and 19 females) with an average age of 52 years (range: 22-83 years) underwent resection. There were 23 (14.4%) well-differentiated hepatocellular HCC patients, 103 (64.4%) moderately differentiated HCC patients, and 34 (21.3%) poorly differentiated HCC patients. There were 95 (58.1%) BCLC stage A patients, 39 (23.8%) BCLC stage B patients, and 29 (18.1%) BCLC stage C patients. Analysis of TNM stage revealed 91 TNM stage I patients (56.9%), 22 stage II patients (13.8%), 44 stage III patients (27.5%), and 3 stage IV patients (1.9%). Within the cohort, all the patients had cirrhosis, and 143 (89.4%) were HBV -positive patients. Intrahepatic metastasis occurred in 21 cases (13.1%), and tumor vascular invasion occurred in 59 cases (36.9%) (**Table S1**).

### Identification of CTC Subpopulations and Nanog-Expressing Cells in the Blood of All Patients

To study the cell capture efficiency, using Can-Patrol™ CTC technology to enrich and detect CTCs in blood samples. The red and green fluorescent signals represent epithelial and mesenchymal gene expression, respectively. The white fluorescent signal represents CD45 gene expression (a leukocyte marker), whereas the violet fluorescent signal represents Nanog gene expression (a CSC marker) (**Figure 1B**, **Table S2**).

For the purpose of classifying and counting CTCs, we used RNA-ISH analysis to divide CTCs into the following five subgroups: 1) Epithelial CTCs (E-CTCs), 2)Epithelial-predominant mixed CTCs (E>M-CTCs), 3) Epithelial/mesenchymal mixed CTCs (E≈M-CTCs), 4) Mesenchymal-predominant mixed CTCs (M>E-CTCs), and 5) mesenchymal CTCs (M-CTCs) (**Figure 1C**). Patient demographics were listed in **Table S1**. Most of the 144 patients (90%) had CTC-positive (EpCAM^+^CK^+^ CD45^-^DAPI ^+^), and 16 patients (10%) had CTC-negative (EpCAM^-^CK^+^CD45^-^DAPI^+^) in their blood samples. Our study found that in CTC-positive HCC patients, epithelial (E type) accounted for 10 (6.3%), mesenchymal (M type) accounted for 121 (75.6%) and mixed type (mixed type) accounted for 13 (8.1%) (**Table S3**).

### Association of CTC Counts and Subtypes With Early Clinical Characteristics

**Figure 2:**
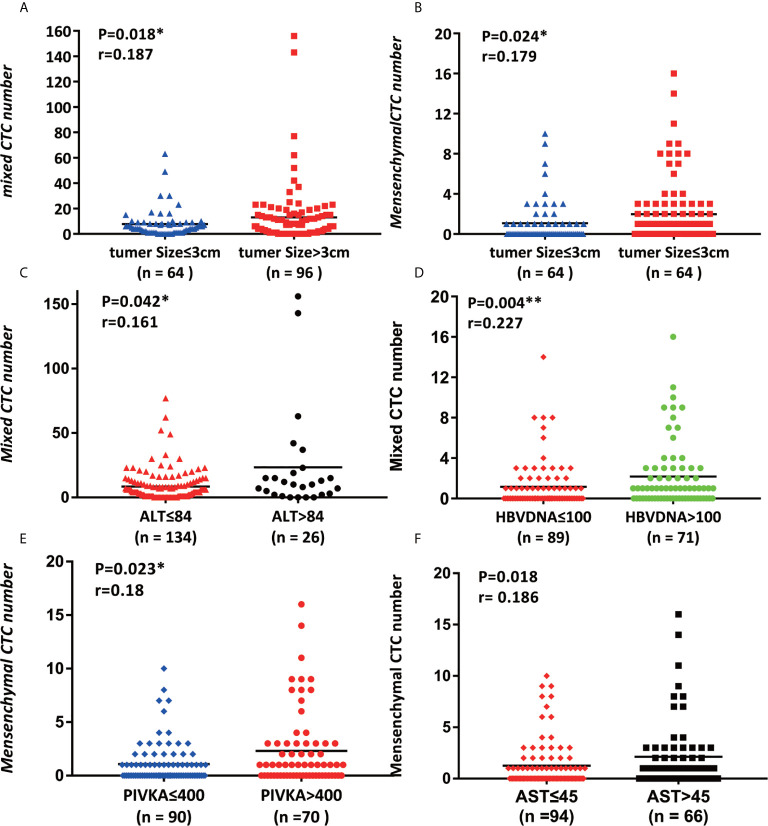
Association of CTC counts and subtypes with early clinical characteristics. **(A, B)** Tumor size was connected with the number of mixed CTCs and the number of mesenchymal CTCs, p<0.05; **(C, D)** alanine transaminase (ALT) and HBV DNA were correlated with the mixed CTC number, p<0.05; **(E, F)** PIVKA and aspartate amino transferase (AST) are related to mesenchymal CTC number, p<0.05.

We first proved that BCLC staging is significantly related to the total number of CTCs (P = 0.002) (**Figure 1D**); Further verify the correlation between various phenotypes of CTC and BCLC staging. Found no correlation between epithelial CTC and BCLC staging (**Figure 1E**). Larger numbers of mixed CTCs tended to be associated with larger tumors (P = 0.018; **Figure 2A**) and the possibility of BCLC (B + C) (P = 0.003; **Figure 1F**). Interestingly, it can also cause high expression of HBV-DNA (P = 0.004; **Figure 2D**), direct bilirubin (P = 0.022) (**Figure S1**) and ALT (P = 0.042) (**Figure 2C**). A high number of mesenchymal CTCs also showed a reasonably high correlation with tumor size (P = 0.024; **Figure 2B**), BCLC stage (P = 0.031; **Figure 1G**), PIVKA (P = 0.023; **Figure 2E**) and AST (P = 0.018; **Figure 2F**) in HCC patients (**Table S3**).

### High Numbers of CTCs Enhance Tumor Vascular Invasion and Promote Tumor Growth

According to the total number of CTCs, the admitted HCC patients are divided into the following categories, with 13.3 designated the cutoff. In patients in the CTC high group, vascular invasion was frequently identified (P = 0.041). The tumor size of the group with a high number of CTCs was larger than that of the group with a low number of CTCs (P=0.008). The two indicators related to hepatitis B, AST (p = 0.048) and HBV-DNA (≤100/>100) (P=0.004), were all proportional to the number of CTCs.

There is no significant correlation between the total number of CTCs and the clinical characteristics of gender, age, and liver cirrhosis, AFP, NLR, INR, Hb, PLT, PT and ICG (**Table 1**).

**Table 1:** Study on the correlation between the total number of CTCs and the clinical characteristics of HCC.

Clinical characteristics	CTC≤13.3	CTC>13.3	P
Sex (Male/Female)	97/16	44/3	0.166
Age (≤60/>60 years)	86/27	37/10	0.721
Tumor Size (≤5/>5 cm)	76/37	21/26	**0.008**
Tumor Number (1/>1)	90/23	32/15	0.118
Differentiation (H/M/L)	20/69/24	3/34/10	0.164
BCLC Stage (A/B+C)	72/41	21/26	**0.026**
Metastasis (Yes/No)	14/99	7/40	0.669
AFP (≤400/>400 μg/L)	72/41	32/15	0.598
PIVKA (≤400/>400 μg/L)	69/44	21/26	0.057
Vascular Invasion (+/-)	36/77	23/24	**0.041**
MVI (Yes/No)	29/84	20/27	**0.035**
MVD (Yes/No)	19/94	9/38	0.723
HBsAg (+/-)	101/12	42/5	0.997
Direct Bilirubin (≤4/>4)	53/60	17/30	0.213
Total Bilirubin (≤10/>10)	12/101	1/46	0.073
ALT (≤84/>84 IU/L)	98/15	36/11	0.114
TNM (I-II/III-IV)	82/31	31/16	0.403
Edmondson stage (H/M/L)	20/69/24	3/34/10	0.164
HBV DNA (≤100/>100)	71/42	18/29	**0.004**
NLR (≤1.77/>1.77)	26/87	12/35	0.733
INR (≤1/>1)	42/71	15/32	0.527
APRI (≤1/>1)	102/11	42/5	0.862
HB (≤120/>120)	13/100	4/43	0.576
Neutrophil (≤4/>4)	85/28	30/17	0.144
Lymphocyte (≤1/>1)	35/78	9/38	0.127
PT (≤12/>12)	63/50	23/24	0.431
PLT (≤100/>100)	32/81	13/34	0.933
AST (≤45/>45)	72/41	22/25	**0.048**
ALB (≤35/>35)	9/104	5/42	0.586
ICG (≤10/>10%)	96/17	40/7	0.981^1^

P value < 0.05 is Statistically Highly Significant (Pearson Correlation Coefficient); INR, International Normalized Ratio; APRI, AST to platelet ratio index; HB, hemoglobin; PT, prothrombin time; PLT, platelets; AST, glutamic oxaloacetic transaminase; ALB, albumin; ICG, indocyanine green. Bold font is used to describe p<0.05, which is statistically significant.

### Correlation Between CTC Number and Nanog Expression in Peripheral Blood and Clinical HCC Tissues

**Figure 3:**
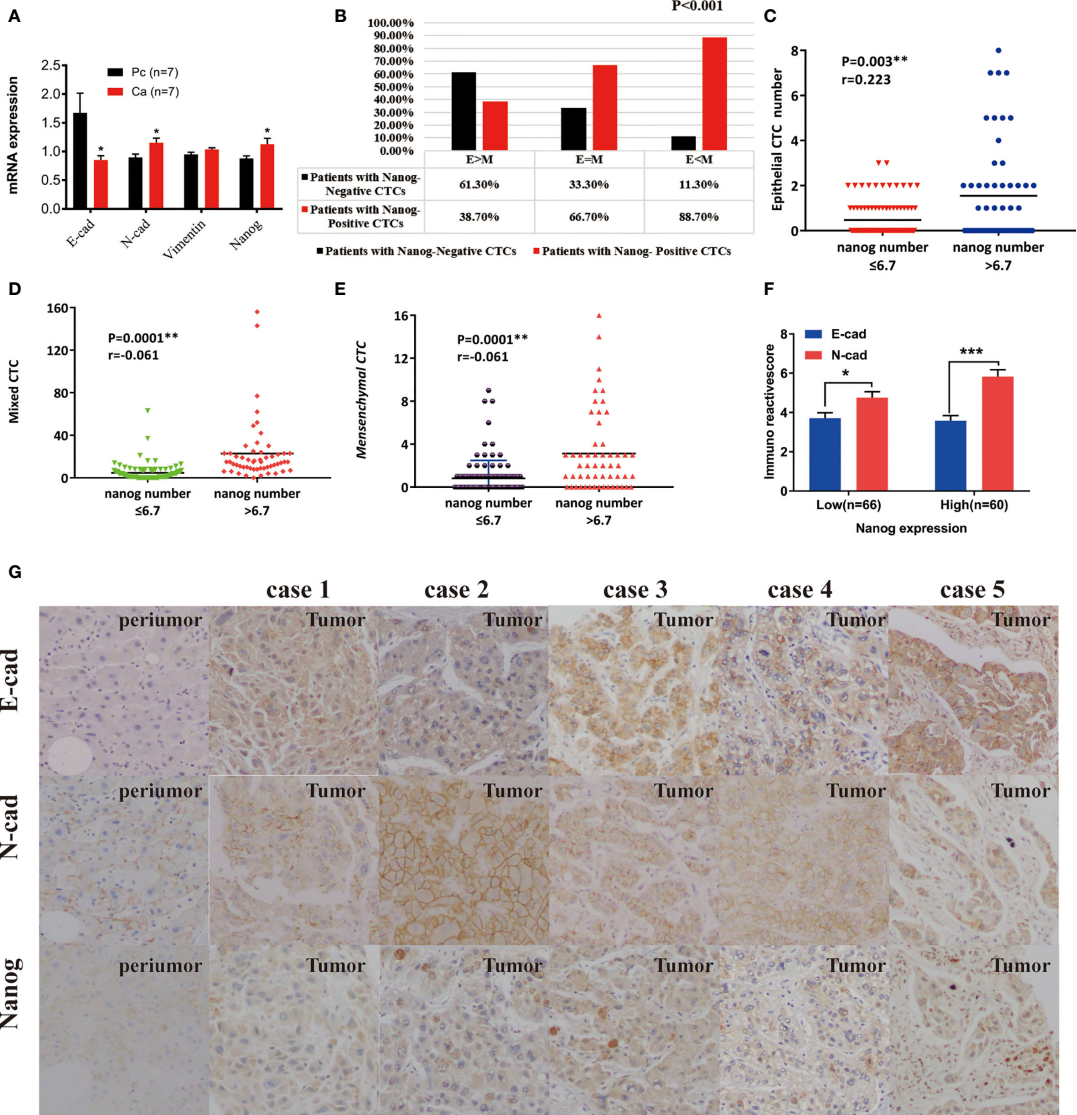
The expression of Nanog and CTCs in liver cancer peripheral blood and clinical liver cancer tissues. **(A)** Expression differences of E-cadherin, N-cadherin, and Nanog in 7 pairs of cancer and adjacent tissues (*P < 0.05 , 2-△△Ct). **(B)** The correlation between the phenotype of CTC and the Nanog positive rate in peripheral blood. **(C–E)** Correlation between phenotype and number of Nanog in peripheral blood. **(F, G)** Immunochemistry : Expression of E-cadherin, N-cadherin and Nanog in liver cancer tissues and their paired adjacent tissues (magnification, ×100 and ×400). ***P < 0.001.

In total, 160 patients (CTCs/5 mL blood) were eligible for analysis of Nanog expression in this study, with 110 patients (68.8%) presenting Nanog-positive blood samples. The percentage of individuals with Nanog-positive cells was 75.7% (109/144). There are significant differences in the expression of Nanog on different types of CTC: most Epithelial CTCs do not express Nanog, while most of Mixed CTC and Mesenchymal CTC express Nanog, and their positive rates are 38.7%, 66.7%, and 88.7%, respectively, (**Figure 3B**, P=0.0001). We found that clinical HCC tissues showed higher relative Nanog, N-cadherin, and Vimentin expression (2-ΔΔCt) than peritumoral liver tissues (p<0.05; **Figure 3A**). The group with high Nanog expression (Nanog>6.7) had a high total number of CTCs (**Figure S2**, P=0.0001), E-CTCs (**Figure 3C**, P=0.003), mixed CTCs (**Figure 3D**, p<0.001), and M-CTCs (**Figure 3E**, p<0.001).In order to explore whether the various phenotypes of CTC and the expression of CSCS marker (Nanog) are related to the occurrence of liver cancer, we used 160 cases of HCC tissue chip array for immunohistochemical analysis. E-cadherin and N-cadherin are located in the cell membrane, and Nanog is located in the nucleus. (**Figures 3F, G**). The expression of N-cadherin and Nanog is positively correlated, and the expression of both in liver cancer tumor tissues is higher than that in adjacent tissues. The expression of E-cadherin was negatively correlated with N-cadherin and Nanog, respectively.

### Nanog Correlates With BCLC Stages and Tumor Growth in Pretreated HCC Patients

The 160 research subjects were divided into three groups according to their ages, young people (≤39), middle-aged people (35, 41, 43–50), and old people (≥50). The results of Fisher's exact test showed that there were 14 young people High expression of nanog occurred in 9 persons (64.3%), 17 out of 50 subjects of middle-aged people had high expression of nanog (34%), and 29 out of 96 subjects of elderly had high expression of nanog (30.2 %) The difference between the above three groups is statistically significant (χ2=5.941, P=0.047). Pairwise comparison found that there was a significant difference in the high expression of Nanog between the young group and the elderly group and middle-aged group (p<0.05) (**Table S6**)

**Figure 4:**
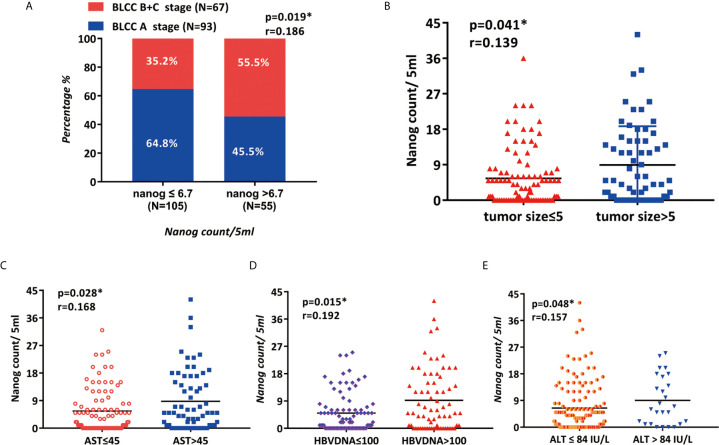
The clinicopathological features of patients are related to the expression of Nanog **(A)** BCLC staging, **(B)** tumor size, **(C)** AST, **(D)** HBV DNA, and **(E)** ALT are associated with Nanog.

The correlation between Nanog expression and the patients’ clinicopathological features was analyzed, and the results are shown in **Table 2**. According to the level of Nanog expression, HCC patients were divided into two groups, with low expression NANOG (Nanog≤6.7) and high Nanog expression (Nanog> 6.7). Patients with high Nanog levels had an increased probability of tumor size > 5 cm (P=0.031; **Table 2**, **Figure 4B**). The number of patients with high Nanog expression in the BCLC stage (B+C) was 54.5% higher than that of patients with low Nanog expression (35.2%) (P=0.019; **Figure 4A**). The incidence of HBsAg-positive status in patients with high Nanog expression was higher than that in patients with low Nanog expression (P=0.038). HBV-DNA (≤100/>100) showed a similar trend in the group with high Nanog expression (P=0.004; **Figure 4D**). In addition, patients who drink heavily are more likely to have higher Nanog expression (67.3%) (P = 0.032). The Nanog level is related to AST (≤45/>45; **Figure 4C**) and ALT (≤84/>84 IU/L; **Figure 4E**). These results suggest a positive trend in the correlation between Nanog expression in CTCs and the degree of tumor malignancy.

**Table 2:** Study on the correlation between clinical liver cancer and the total number of Nanog ^+^ CSCs.

Clinical Characteristics	Nanog≤6.7	Nanog>6.7	P
Sex (Male/Female)	92/13	49/6	0.785
Age (≤60/>60 years)	78/27	45/10	0.283
Age(≤39/40-49/≥50)	5/33/67	9/17/29	0.047
Tumor Size (≤5/>5 cm)	70/35	27/28	**0.031**
Tumor Number (≤1/>1)	79/26	43/12	0.678
Differentiation (H/M/L)	18/65/22	5/38/12	0.381
BCLC Stage (A/B+C)	68/37	25/30	**0.019**
Metastasis (Yes/No)	14/91	7/48	0.914
AFP (≤400/>400 μg/L)	66/39	38/17	0.432
PIVKA (≤400/>400 μg/L)	61/44	29/26	0.516
Vascular Invasion (+/-)	34/71	25/30	0.104
MVI (Yes/No)	29/76	20/35	0.254
MVD (Yes/No)	17/88	11/44	0.547
HBsAg (+/-)	90/15	53/2	**0.038**
CTC (≤13.3/>13.3)	96/9	17/38	**0.0001**
Direct Bilirubin (≤4/>4)	49/56	21/34	0.304
Total Bilirubin (≤18/>18)	60/45	31/24	0.925
ALT (≤84/>84 IU/L)	92/13	42/13	0.067
TNM (I-II/III-IV)	76/29	37/18	0.5
Edmondson Stage (I-II/III-IV)	71/34	37/18	0.965
HBV DNA (≤100/>100)	67/38	22/33	**0.004**
NLR (≤1.77/>1.77)	24/14	81/41	0.714
INR (≤1/>1)	38/67	19/36	0.937
APRI (≤1/>1)	95/10	49/6	0.781
HB (≤120/>120)	12/93	5/50	0.649
Neutrophil (≤4/>4)	80/25	35/20	0.093
Lymphocyte (≤1/>1)	34/71	10/45	0.056
PT (≤12/>12)	57/48	29/26	0.851
PLT (≤100/>100)	33/72	12/43	0.199
AST (≤45/>45)	67/38	27/28	0.072
ALB (≤35/>35)	9/96	5/50	0.912
ICG (≤10/>10%)	89/16	47/8	0.907^2^

P value < 0.05 was considered statistically significant (Pearson Correlation Coefficient); INR, International Normalized Ratio; APRI, AST to platelet ratio index; HB, hemoglobin; PT, prothrombin time; PLT, platelets; AST, glutamic oxaloacetic transaminase; ALB, albumin; ICG, indocyanine green. Bold font is used to describe p<0.05, which is statistically significant.

### Nanog^+^ CTC Expression Is Positively Correlated With Recurrence

**Figure 5:**
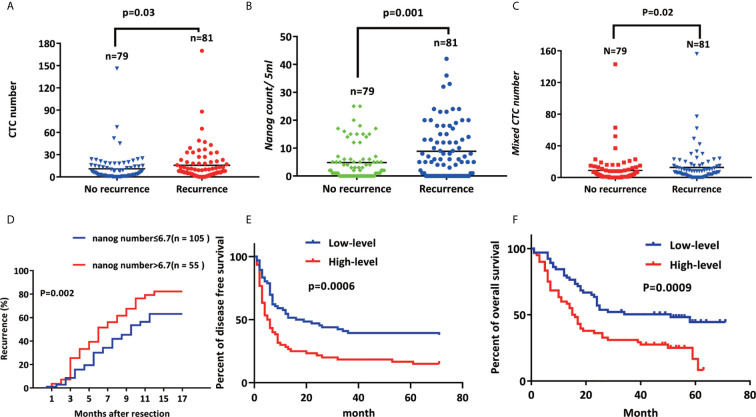
Nanog^+^ CTC expression is positively correlated with recurrence **(A)** CTC number, **(B)** Nanog count, **(C)** The higher the expression of Mixed CTC number and Nanog, the higher the early recurrence rate of liver cancer; **(D)** Kaplan–Meier estimates of disease-free survival **(E)** (p<0.001) and overall **(F)** (p<0.001) survival of 160 HCC patients with high-level Nanog (≥500 μm2/field) and low-level Nanog (< 500 μm2/field).

The one-year recurrence rate of patients with high Nanog expression (69.1%) was higher than that of patients with down-regulated expression (41.0 %) (48). Furthermore, the total number of CTCs (P =0.03; **Figure 5A**), mixed CTC count (P=0.02; **Figure 5B**) and Nanog levels (P = 0.016; **Figure 5C**) were all factors that led to the recurrence of liver cancer (**Table S4**). During the follow-up, 81 cases (50.6%) recurred, the BCLC stage A recurrence rate was 46.2% (43/93), and the BCLC stage (B+C) recurrence rate was 56.7% (38/67) (**Table S4**). These results suggest that Nanog expression, CTC count, mixed CTC count, and BCLC stage before surgery are significantly correlated with recurrence.

### AFP and Nanog as Key Prognostic Factors

We have used univariate and multivariate Cox analysis to construct a prognostic model for HCC. Univariate analysis of prognostic factors of HCC has been the following: Tumor size > 5 cm (P =0.049), BCLC stage (B+C) (P = 0.024), AFP > 400 μg/L (P = 0.002), PIVKA > 400 μg/L (P=0.039), Nanog > 6.7 (P = 0.005), and HBV-DNA > 100 (P=0.017). In addition, we have obtained the following results using multivariate Cox analysis:AFP > 400 μg/L [P=0.000067, HR (95% CI) = 2.733 (1.667-4.481)] and Nanog > 6.7 [P = 0.000282, HR (95% CI) = 2.33 (1.476-3.679)] were independent factors of prognosis. These two factors were identified as key prognostic factors (**Table 3**) (48).

**Table 3:** Cox proportional regression analysis of univariate and multivariate.

Variables	Univariate analysis		Multivariate analysis	
	HR (95% CI)	P	HR (95% CI)	P
Sex (Male/Female)	1.6(0.845-3.033)	0.149		
Age (≤60/>60 years)	0.977(0.594-1.607)	0.926		
Tumor Size (≤5/>5 cm)	1.557(1.002-2.417)	**0.049**	.	
Tumor Number (≤1/>1)	1.613(0.984-2.644)	0.058	.	
Differentiation (H/M/L)	1.466(0.704-3.053)	0.307		
BCLC Stage (A/B+C)	1.657(1.067-2.572)	**0.024**	.	
Metastasis (Yes/No)	1.892(0.958-3.737)	0.066		
AFP (≤400/>400 μg/L)	2.042(1.303-3.199)	**0.002**	2.733(1.667-4.481)	**0.000067**
PIVKA (≤400/>400 μg/L)	1.593(1.023-2.481)	**0.039**		
E CTC (+/-)	1.295(0.827-2.026)	0.258		.
Mixed CTC (+/-)	1.068(0.577-1.976)	0.835		
M CTC (+/-)	1.182(0.764-1.83)	0.452		
CTC (+/-)	1.233(0.567-2.680)	0.597		
CTC (≤13.3/>13.3)	1.039(0.648-1.668)	0.872		.
Nanog (≤6.7/>6.7)	1.881(1.215-2.911)	**0.005**	**2.33(1.476-3.679)**	**0.000282**
Vascular thrombus (+/-)	0.913(0.577-1.445)	0.697		
MVI (Yes/No)	0.795(0.49-1.292)	0.355	0.542(0.311-0.943)	**0.03**
MVD (Yes/No)	1.711(0.998-2.932)	0.051	2.418(1.331-4.392)	**0.004**
HBsAg (+/-)	1.816(0.835-3.953)	0.132		.
Direct Bilirubin (≤4/>4)	0.873(0.562-1.356)	0.546	.	.
Total bilirubin (≤10/>10)	0.747(0.342-1.722)	0.493	.	
ALT (≤84/>84 IU/L)	0.866(0.469-1.6)	0.647		
TNM (I-II/III-IV)	1.318(0.826-2.106)	0.247		.
Edmondson (I-II/III-IV)	1.498(0.945-2.376)	0.086		
HBV DNA (100≤/>100)	1.711(1.099-2.662)	**0.017**		.
NLR (≤1.77/>1.77)	0.74(0.446-1.23)	0.245	.	.
INR (≤1/>1)	0.966(0.593-1.574)	0.89		
APRI (≤1/>1)	0.638(0.293-1.389)	0.257	0.382(0.169-0.863)	**0.021**
HB (≤120/>120)	1.43(0.685-2.984)	0.341		.
Neutrophil (≤4/>4)	0.921(0.555-1.528)	0.75	0.543(0.298-0.991)	**0.047**
Lymphocyte (≤1/>1)	1.224(0.757-1.979)	0.409		
PT (≤12/>12)	0.938(0.604-1.456)	0.774	.	
PLT (≤100/>100)	1.308(0.809-2.116)	0.274		
AST (≤45/>45)	1.132(0.715-1.791)	0.597	.	
ALB (≤35/>35)	0.734(0.367-1.469)	0.382	.	
ICG (≤10/>10%)	1.058(0.584-1.916)	0.852		

Bold font is used to describe p<0.05, which is statistically significant.

Use immunohistochemistry to observe the expression of nanog in liver cancer and adjacent cancers to determine the content of CSCs. The density of CSCs was quantified, and liver tumor tissues from patients were designated high-level or low-level densities (compared to those in para carcinoma tissue) (**Figure 3F**). According to the correlation analysis of clinicopathological characteristics, Nanog concentration is positively correlated with liver cancer vascular invasion, metastasis, capsular infiltration, tumor stage (BCLC, TNM) and differentiation. However, gender, age, lymph node metastasis or tumor size are not related to Nanog expression. Next, we performed the correlation analysis between nanog concentration and patient survival. The study found that overall survival (OS) and disease-free survival are significantly related to the intensity of Nanog expression (**Figures 5E, F**). The above results indicate that high expression of Nanog results in poor prognosis for patients.

## Discussion

The study found that the content of Nanog ^+^ CTCs in HCC patients is high In addition, high expression of Nanog^+^CTCs was relevant to poor OS and disease-free survival and acted as an independent factor for predicting OS and disease-free survival. What's more, by merging Nanog expression into an integrated prediction model, the prognostic precision of the CTC phenotype in patients with HCC was significantly enhanced.

Quantification of CTCs in breast cancer (6, 13), non-small-cell lung cancer (NSCLC) (14), colorectal cancer (46), bladder cancer (16), HCC (12, 50), melanoma (50), and pancreatic cancer has been shown in connection with survival and recurrence. Currently, the prediction of recurrence is mainly conducted with imaging or measuring biomarkers, but these methods cannot discern dynamic changes in the tumor microenvironment (51, 52). According to previous reports, the prediction of liver cancer recurrence and metastasis is affected by tumor cells and the tumor immune microenvironment (53–55). It was reported that molecular markers expressed on CTCs are compactly related to the early diagnosis and early recurrence of liver cancer (18, 38, 56). High Nanog expression is expressively associated with a high percentage of tumor recurrence and survival (19, 57, 58). Our research found that the expression of Nanog on different types of CTCs is significantly different: most epithelial CTCs do not express Nanog, while most mixed CTCs and mesenchymal CTCs express Nanog, and the positive rates are 38.7%, 66.7%, and 88.7%, respectively. (**Figure 3B**, P = 0.0001). Consistent with the results of these studies, we determined that HCC patients with high Nanog-CTC content had a shorter progression-free survival period and proposed that Nanog be included as a marker for measuring liver cancer metastasis. Our research found that the higher the content of Nanog+ cells, the higher the expression of Vimentin. The high Nanog expression group was strongly associated with E-CTC (**Figure 3C**, p = 0.003), mixed CTC (**Figure 3D**, P < 0.001), and M-CTC (**Figure 3E**, P < 0.001). Interestingly, the high CTC expression group had a high vascular infiltration rate (P = 0.041), which enhanced the intrahepatic and extrahepatic metastasis rate of liver cancer and affected the tumor microenvironment. Liver cancer cells spread from the primary lesion to form CTCs (7, 8, 58). The higher the expression of CSCs in the tumor, the faster the cell self-renewal cycle, and the more unstable the gene, the faster the tumor’s progression (16). Cancer cells with distal organ colonization have the characteristics of CSCs and exert their tumorigenic ability under adverse microenvironmental conditions. Indeed, the latest clinical data suggest that CSC-associated molecular markers are potential surrogate markers for CSC density and can be used as markers for predicting and evaluating tumor progression (1, 59). The higher the content of tumor stem cell-like cells, the worse the prognosis (60, 61). CSCs can also fall off from tumor masses and enter the blood circulatory system like CTCs. They can evade the immune system even during systemic and local treatments, exist in the circulatory system, and re-seed at new sites to produce new local tumors, causing Intrahepatic metastasis (55).

According to previous studies, tumor stem cell-like cells can cause rapid tumor progression and increased resistance to chemotherapy drugs, resulting in poor clinical prognosis in patients with liver cancer (62, 63). The high early mortality and metastasis rate of liver cancer is due to the spread of tumor cells from the original place into the blood We take the content of Nanog^+^ CSCs in the blood and tissues of cancer patients as a new target for evaluating the survival and recurrence of cancer patients.

Here, Nanog+ CSC plays a supplementary part in determining the clinical utility of blood-borne CTCs analysis (P = 0.0001). This result also further validated the concept that Nanog ^+^ CSC can be used as a “soil” to help (“seed”) CTC promote the self-renewal of cancer stem cells and increase drug resistance, and promote the proliferation and metastasis of liver cancer.

Through the single-factor Cox regression analysis method, we found that the following clinical indicators are the key factors for the early recurrence and poor prognosis of liver cancer: Tumor size > 5 cm (P =0.049), BCLC stage (P = 0.024), AFP > 400 μg/L (P = 0.002), PIVKA > 400 μg/L (P = 0.039), Nanog>6.7 (P = 0.005), and HBV-DNA >100 (P = 0.017). Further multivariate Cox analysis revealed that Nanog > 6.7 (P = 0.000282, HR = 2.33) and AFP > 400 μg/L (P = 0.000067, HR =2.733) are significant and independent prognostic biomarkers for early recurrence. Through the analysis of the survival curve, it is found that the higher the expression of Nanog (P = 0.002), the greater the total number of CTCs (P = 0.03), and the greater the content of mixed CTCs (P = 0.02), the recurrence rate of primary liver cancer after resection increases significantly. We found that the higher the total number of CTCs and Nanog^+^ CSCs, the higher the early recurrence rate. Moreover, Our study found that CTCs with a stem cell-like phenotype are highly aggressive, more likely to invade the circulatory system, more easily metastasize from the original tumor to the distal end, and have an increased recurrence rate. In most tumors, poor prognosis and tumor invasion and metastasis are both positively correlated with the content of CSCs (29, 39, 61, 64). However, the mechanisms by which CTCs and Nanog interact remain unclear, which warrants future large-scale clinical studies and further basic exploratory research.

## Conclusions

In summary, the number of Nanog-positive cells is correlated with the poor prognosis of HCC patients. The combined measurement of CTCs and CSCs can improve prognosis. In general, despite the limitations and heterogeneity of negative results found in our study, there are still a large number of studies that prove that CTC and its EMT phenotype, nanog^+^CSCs are highly correlated with tumor staging, vascular invasion and metastasis and predicting HCC recurrence. There is no doubt that the detection and analysis of CTC markers (EpCAM and CK8, 18, CD45 Vimentin, Twist and 19) and CSCs markers (Nanog) are useful for evaluating tumor progression, developing tumor drugs, and monitoring the disease process of cancer patients. etc., are valuable research.

## Data Availability Statement

The original contributions presented in the study are included in the article/**Supplementary Material**. Further inquiries can be directed to the corresponding authors.

## Ethics Statement

The studies involving human participants were reviewed and approved by Ethics Committee of the First Affiliated Hospital of the Army Military Medical University (201703). The patients/participants provided their written informed consent to participate in this study.

## Author Contributions

Conception/design: YL and JW. Provision of study materials or patients: FX. Collection and/or assembly of data: YL, XW, YF, HS, and LY. Data analysis and interpretation: YL and XW. Manuscript writing: YL, FX, and JW. Financial approval of manuscript: YL, XW, HS, YF, LY, JW, and FX. All authors contributed to the article and approved the submitted version.

## Funding

This work is supported by the Natural Science Foundation of China (81773140) and the Foundation and Advanced Research Project of CQ CSTC (cstc2018jscx-mszd0280, cstc2017shmsA130007).

## Conflict of Interest

The authors declare that the research was conducted in the absence of any commercial or financial relationships that could be construed as a potential conflict of interest.

## Nomenclature

BCLC stage, Barcelona Clinical Liver Cancer; PIVKA, Abnormal prothrombin; INR, International Normalized Ratio; PLT, Platelets; ICG, Indocyanine green; HCC, hepatocellular carcinoma; TNM, Tumor Node Metastasis; MVI, microvascular invasion; RT-PCR, reverse Transcription-Polymerase Chain Reaction; APRI, AST to platelet ratio index; INR, international normalized ratio; AFP, Alpha fetoprotein; NLR, Neutrophil To Lymphocyte Ratio; Hb, Hematocrystallin; PT, Prothrombin time; EMT, epithelial-mesenchymal transition; HBV, hepatitis B virus; AST, glutamic oxaloacetic transaminase; ALT, alanine aminotransferase; ALB, albumin; MVD, Microvesseldensity.
