# Corrigendum: Association of preoperative NANOG-positive circulating tumor cell levels with recurrence of hepatocellular carcinoma

**DOI:** 10.3389/fonc.2024.1438731

**Published:** 2024-07-10

**Authors:** Yongrong Lei, Xishu Wang, Heng Sun, Yuna Fu, Yichen Tian, Ludi Yang, Jianhua Wang, Feng Xia

**Affiliations:** ^1^ Key Laboratory of Biorheological Science and Technology, Ministry of Education, College of Bioengineering, Chongqing University, Chongqing, China; ^2^ Key Laboratory of Hepatobiliary and Pancreatic Surgery, Institute of Hepatobiliary Surgery, Southwest Hospital, the First Hospital Affiliated to AMU (Southwest Hospital), Chongqing, China

**Keywords:** recurrence, cancer stem cells, hepatocellular carcinoma, circulating tumor cells, epithelial-mesenchymal

In the published article, there was an error in **Figure 1** as published. In the published article, there were errors in two parts of **Figure 1** as published. In **Figure 1B**, an error occurred in the arrangement of the images. The image corresponding to Mixed CTC’s CD45 (white) was mistakenly placed in the third row, third column, while the image for Mixed CTC’s Mesenchymal (green) was incorrectly positioned in the third row, fourth column. The positions should be swapped to correctly represent the respective markers. In **Figure 1C**, an error was identified in the second column of the E≈M group. The red fluorescence channel image used in the three-color fluorescence (red, green, blue) image was incorrect and does not accurately represent the intended data. The corrected **Figure 1** appears below.

**Figure 1:**
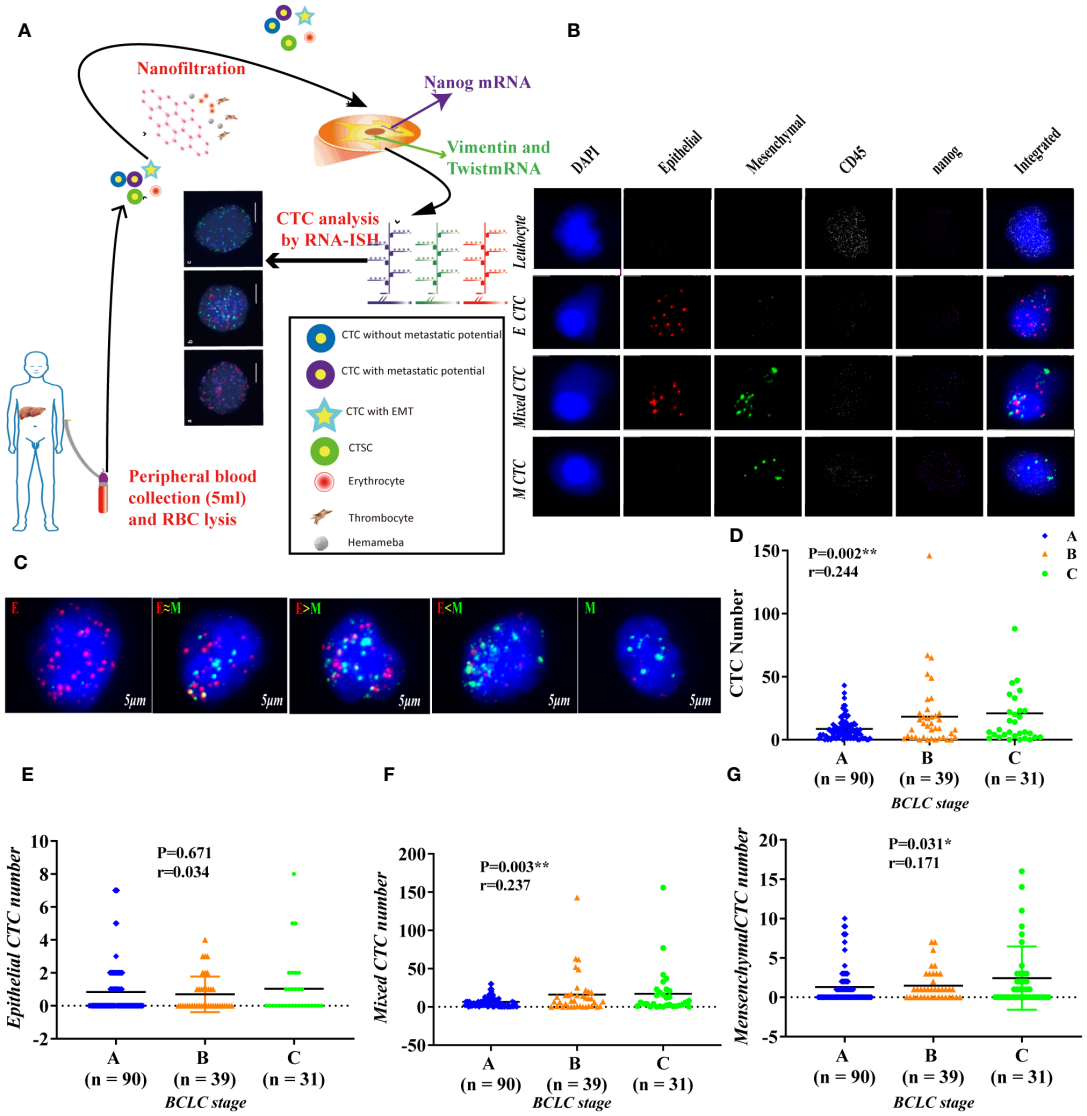
Use RNA-ISH technology to enrich and analyze CTCs in blood samples of HCC patients **(A)** The expression of epithelial marker mRNA (EpCAM and E-cadherin) and **(B)** mesenchymal marker mRNA (Twist and vimentin) and CSC marker mRNA (nanog) were validated by qPCR, p<0.05; **(C)** The protein expression levels of epithelial markers (EpCAM and E-cadherin), p<0.001. **(D–G)** The total number of CTCs, epithelial CTCs, mixed CTCs, and mesenchymal CTCs correlated with BCLC staging, p<0.05. **represents P<0.01, *represents P<0.05.

The authors apologize for this error and state that this does not change the scientific conclusions of the article in any way. The original article has been updated.

