# Supplementary material for: Association of Preoperative NANOG-Positive Circulating Tumor Cell Levels With Recurrence of Hepatocellular Carcinoma
Source: Front Oncol. 2021 May 27;11:601668. doi: 10.3389/fonc.2021.601668 (PMC8190394; doi:10.3389/fonc.2021.601668)
Supplement: Supplementary file 1 [file DataSheet_1.pdf]

Immunohistochemical microarray data

E-cad

2014007 ca pc N-CAD NANOH

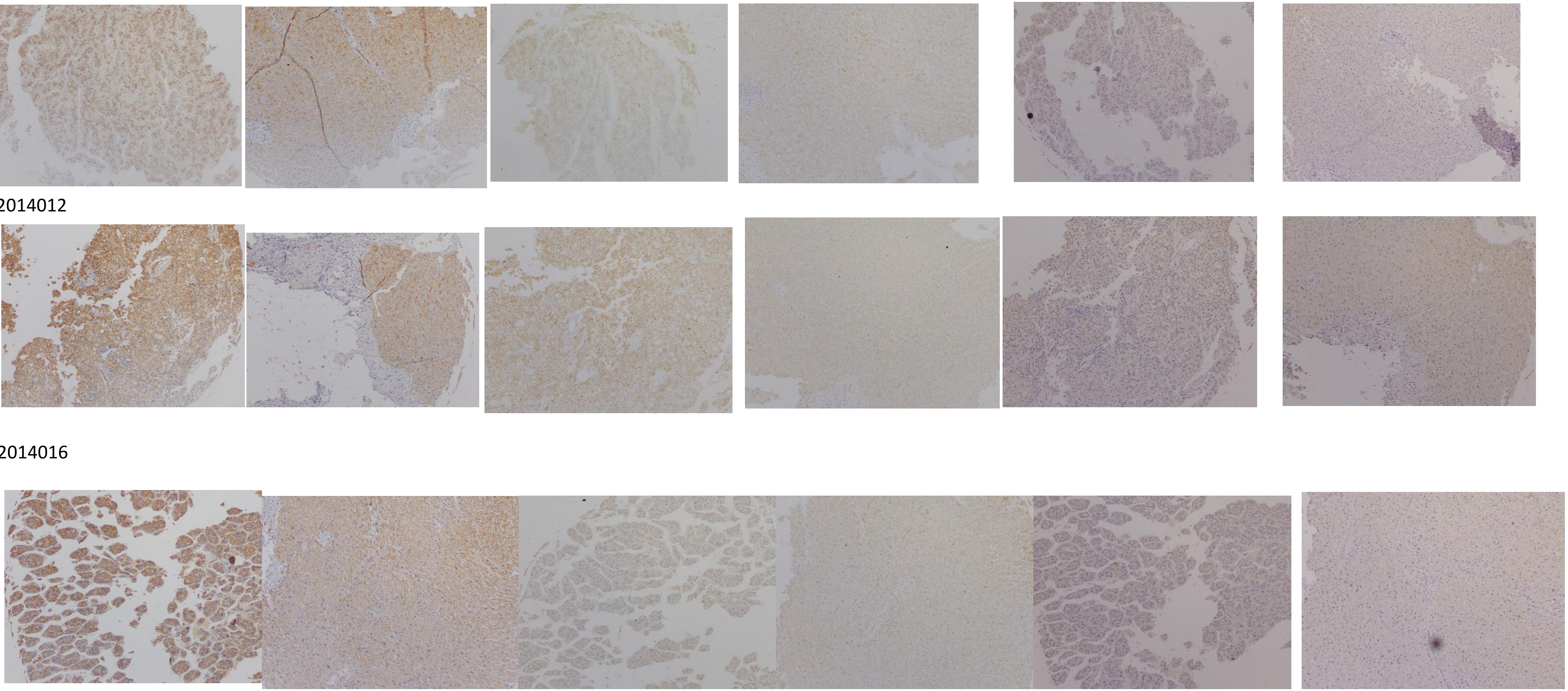

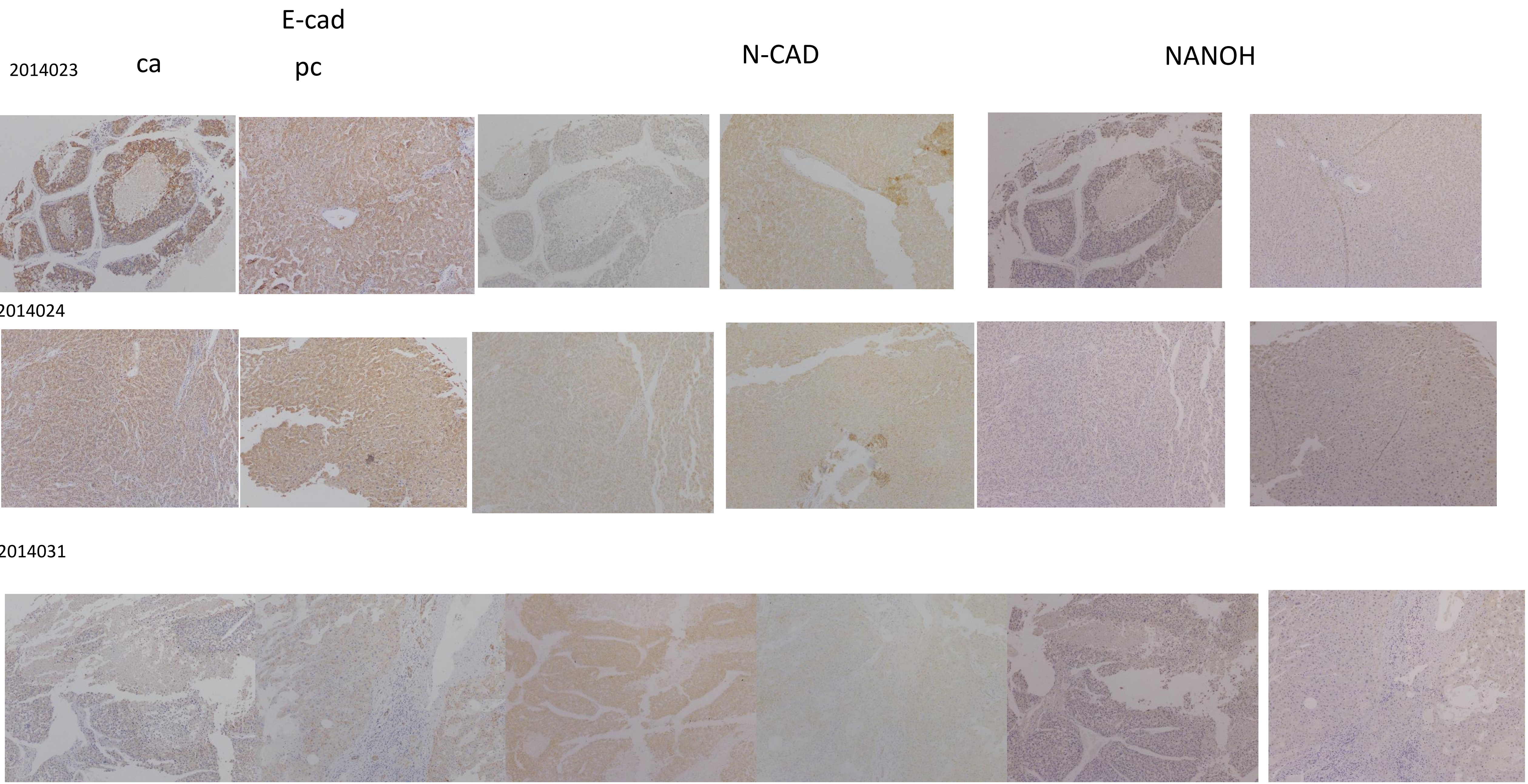
E-cad
2014023
ca
pc
N-CAD
NANOH
2014024
2014031

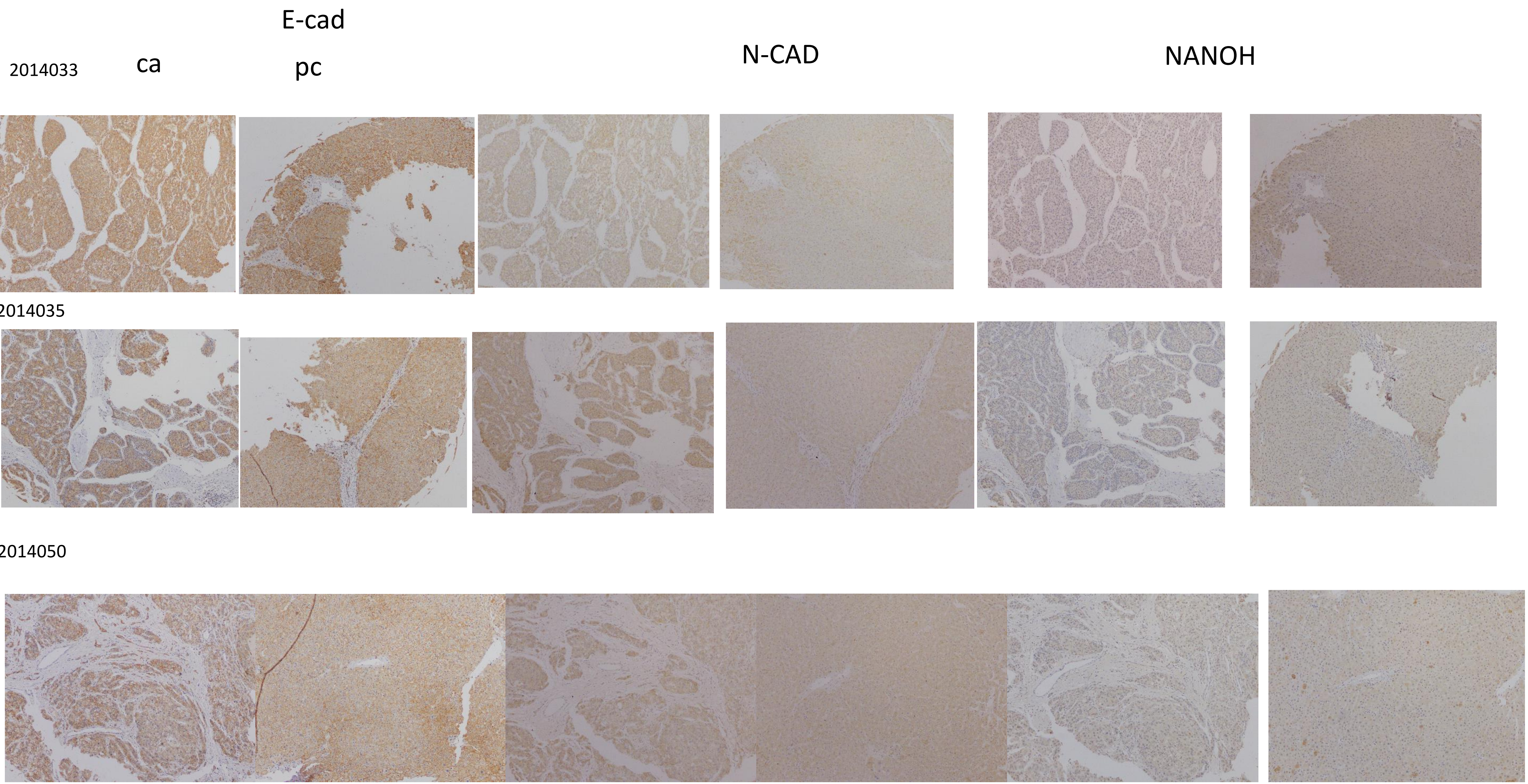
E-cad
2014033
ca
pc
N-CAD
NANOH
2014035
2014050

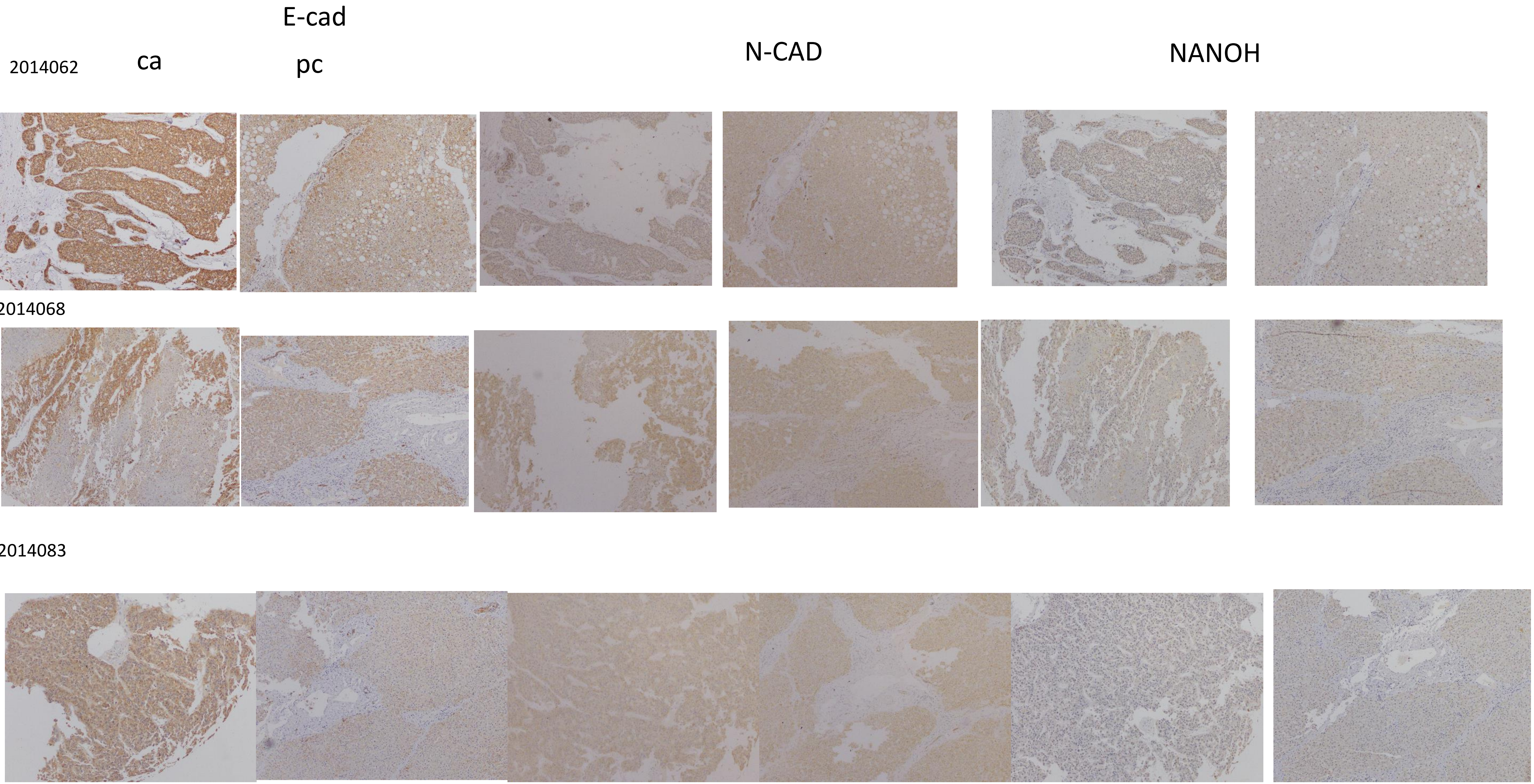
E-cad
N-CAD
NANOH
2014062
ca
pc
2014068
2014083

E-cad

2014095 ca pc N-CAD NANOH

2014096

2014108

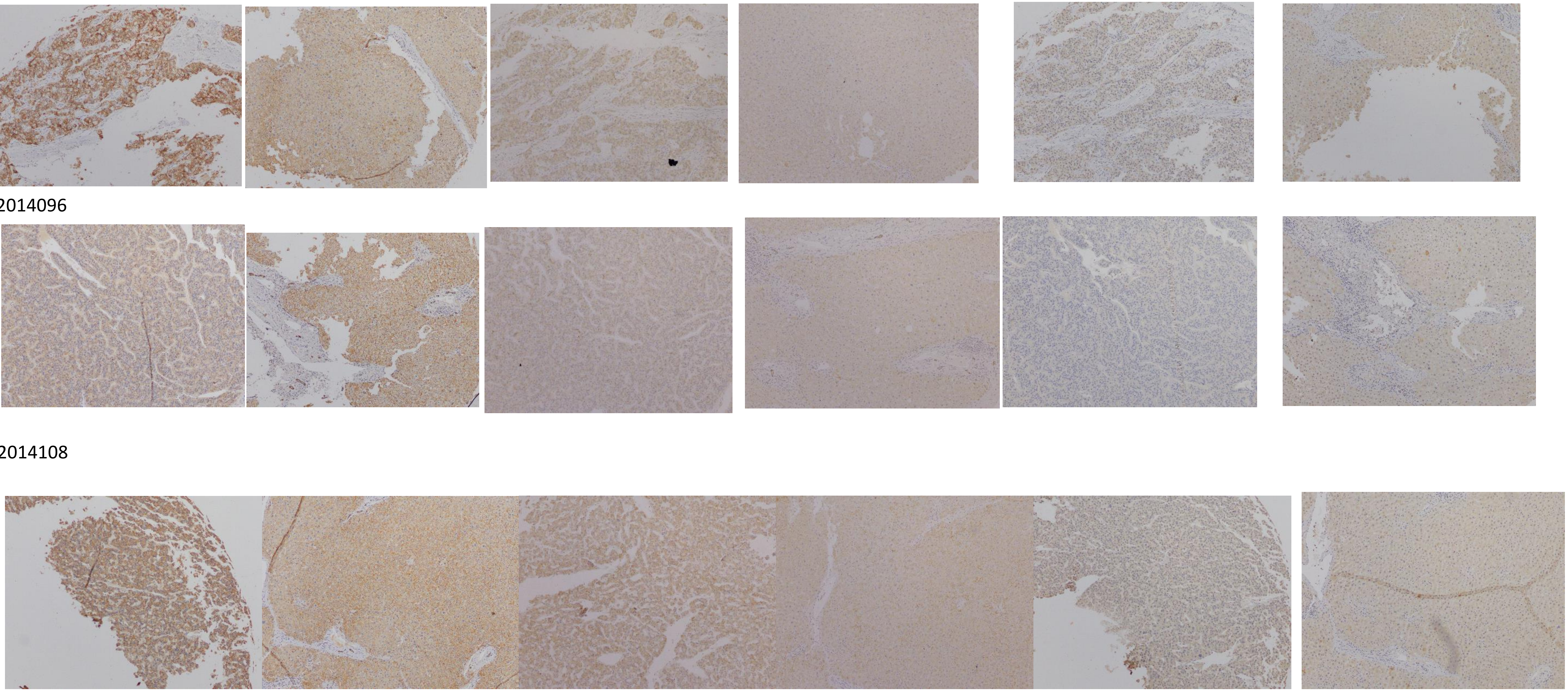

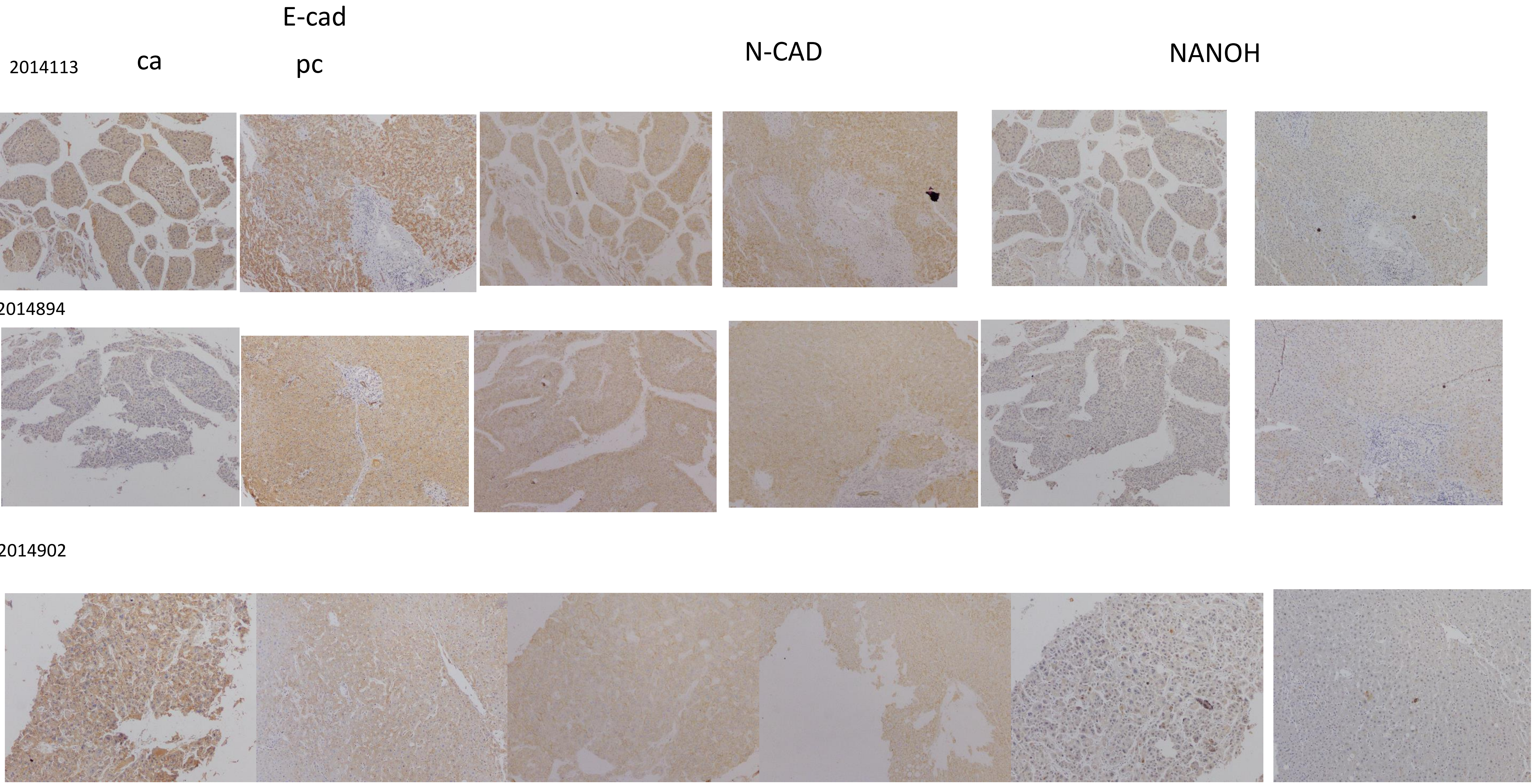
E-cad
2014113
ca
pc
N-CAD
NANOH
2014894
2014902

E-cad

N-CAD

NANOH

2015010 ca pc

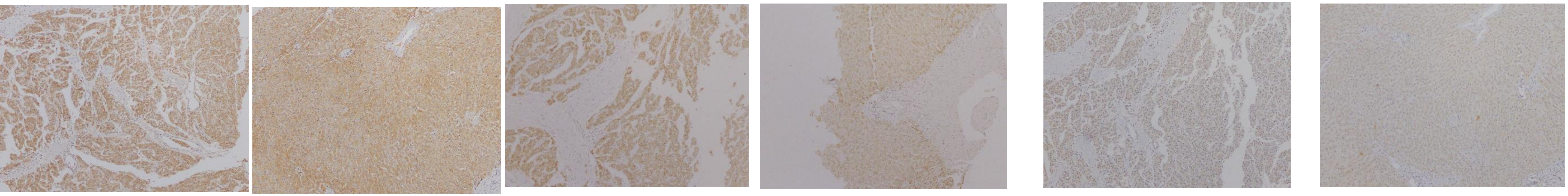

2015012

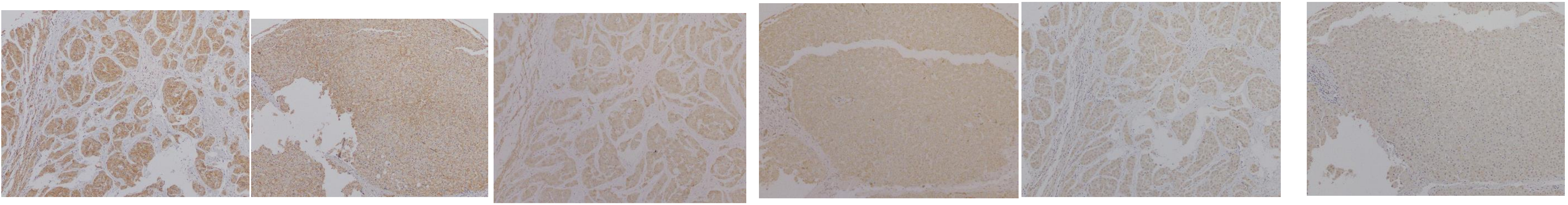

2015041

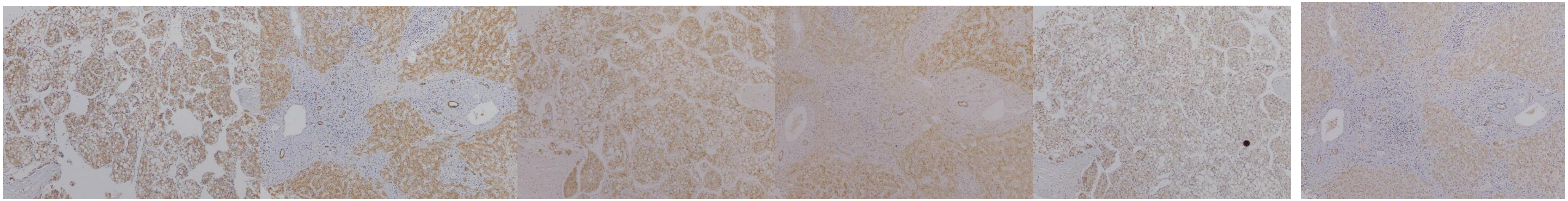

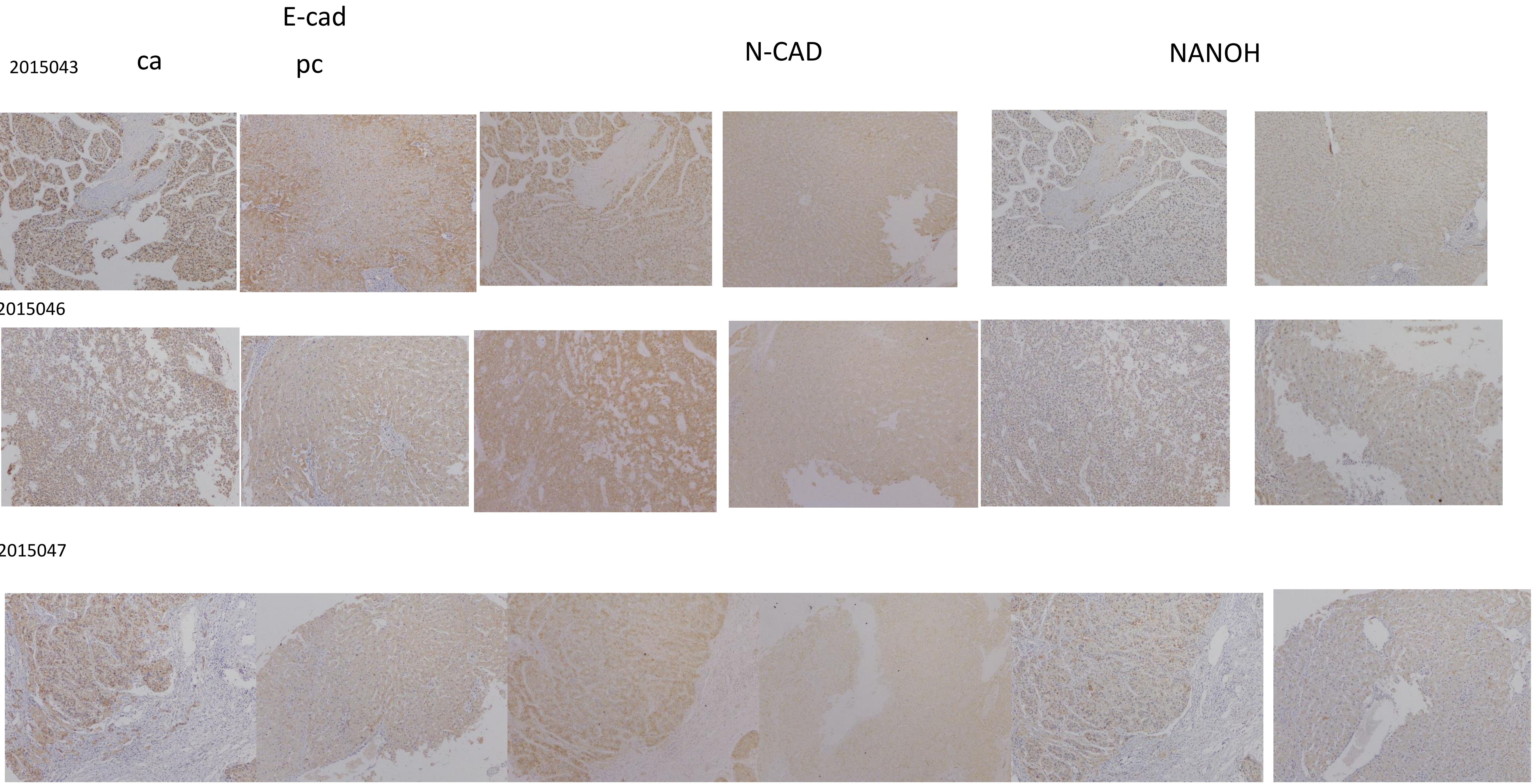
E-cad
2015043
ca
pc
N-CAD
NANOH
2015046
2015047

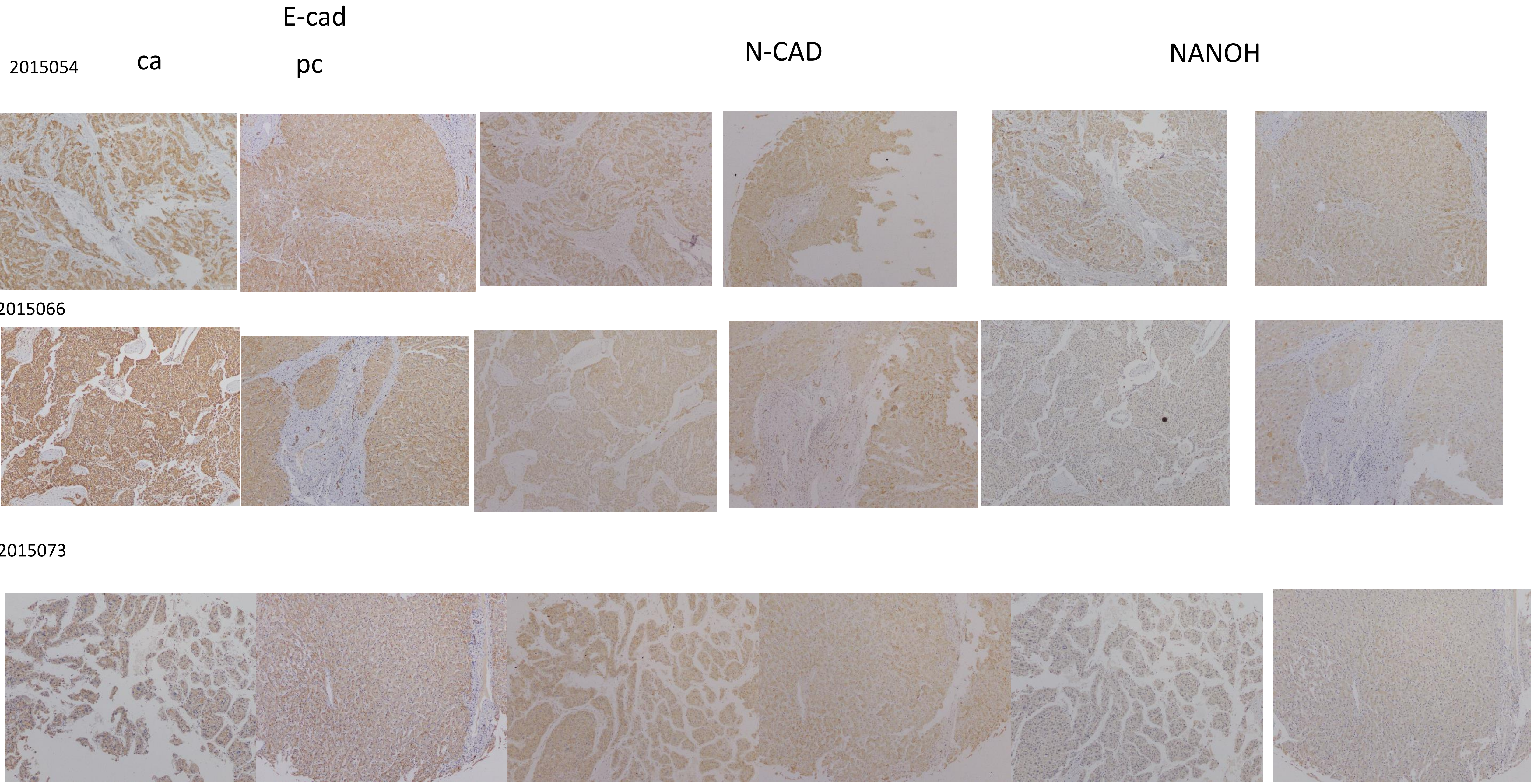

E-cad
2015054
ca
pc
N-CAD
NANOH
2015066
2015073

E-cad

2015083 ca pc N-CAD NANOH

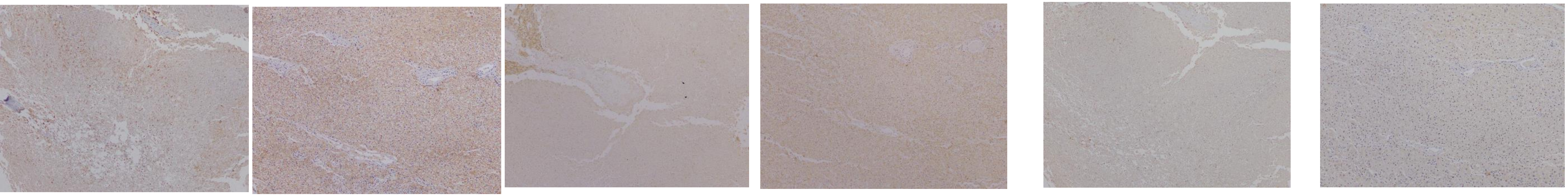

2015105

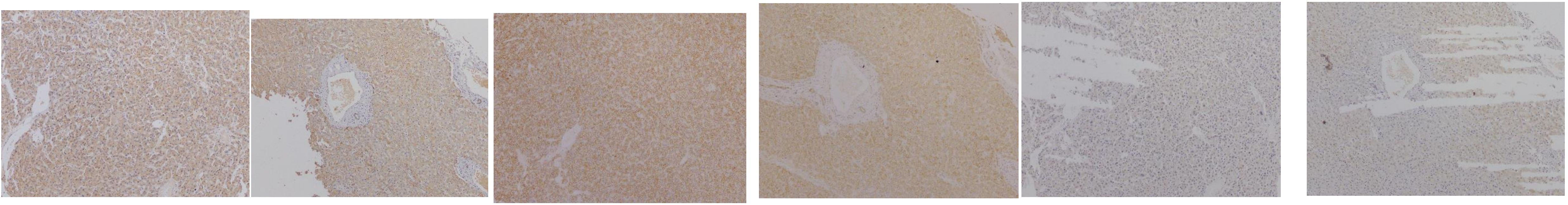

2015142

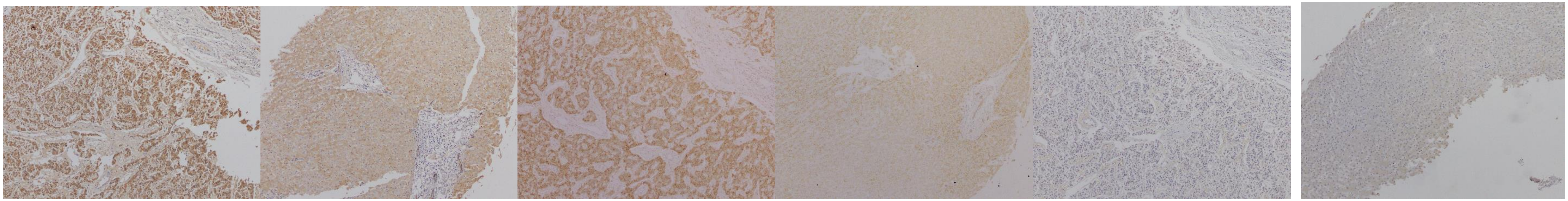

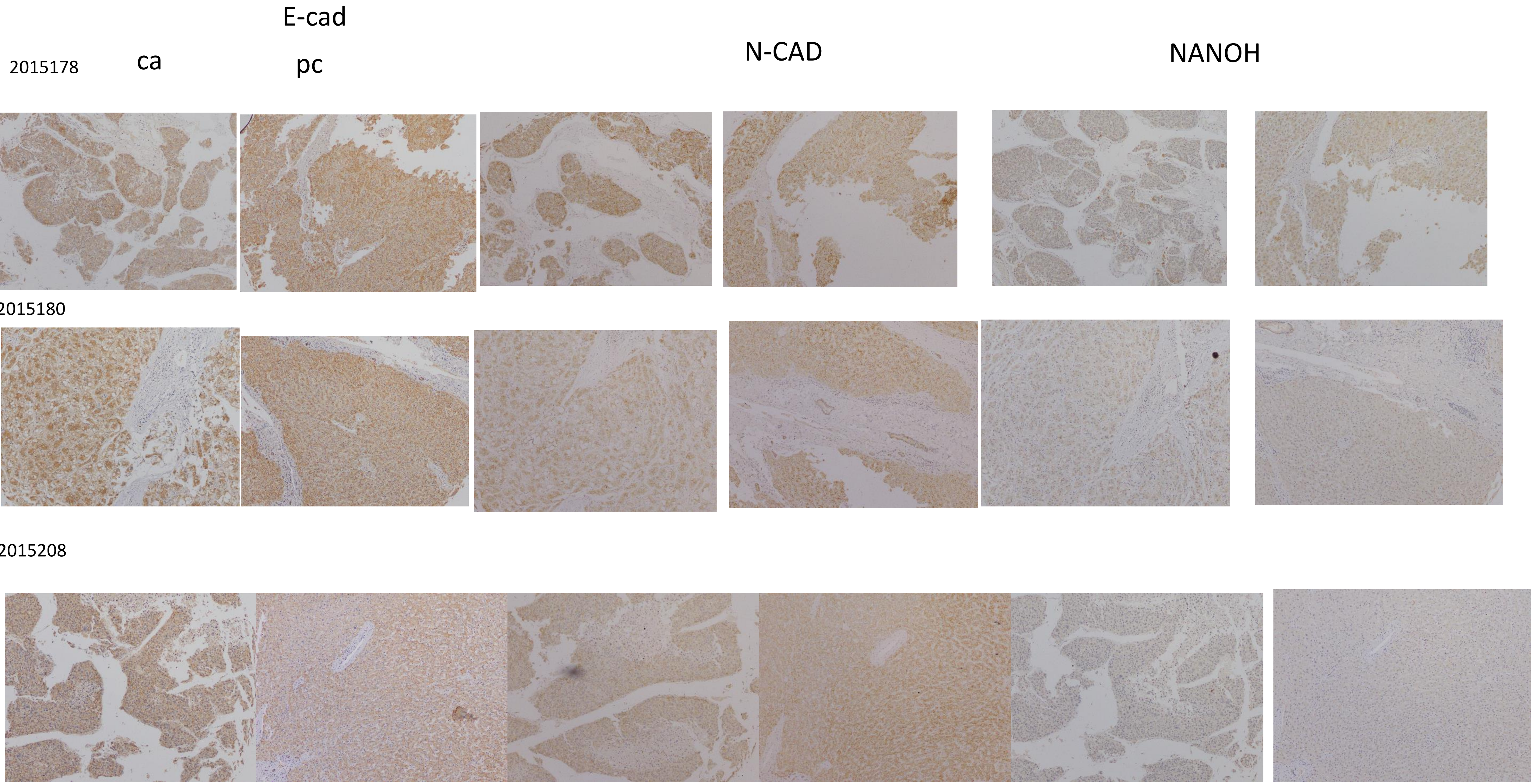
E-cad
2015178
ca
pc
N-CAD
NANOH
2015180
2015208

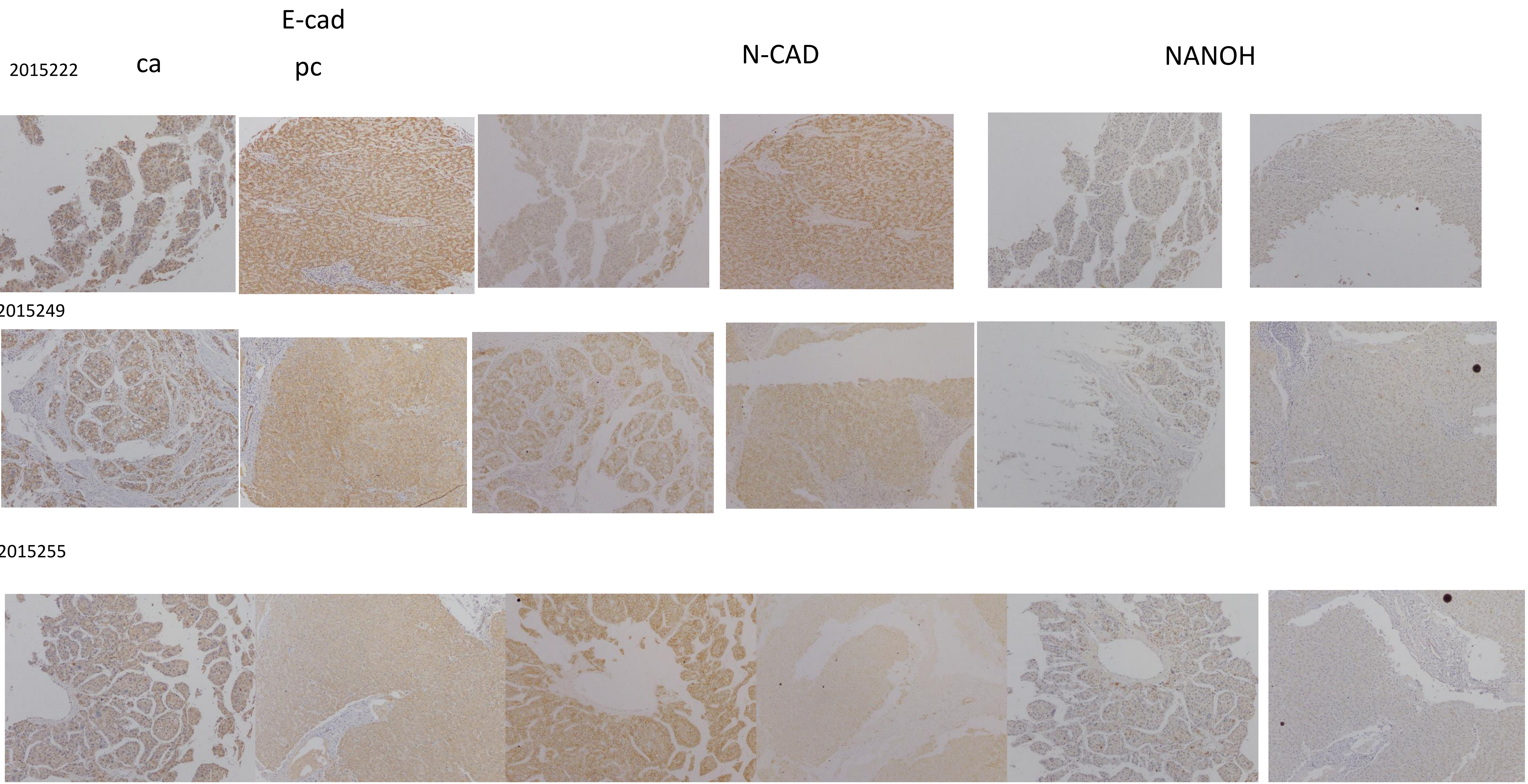
E-cad
2015222
ca
pc
N-CAD
NANOH
2015249
2015255

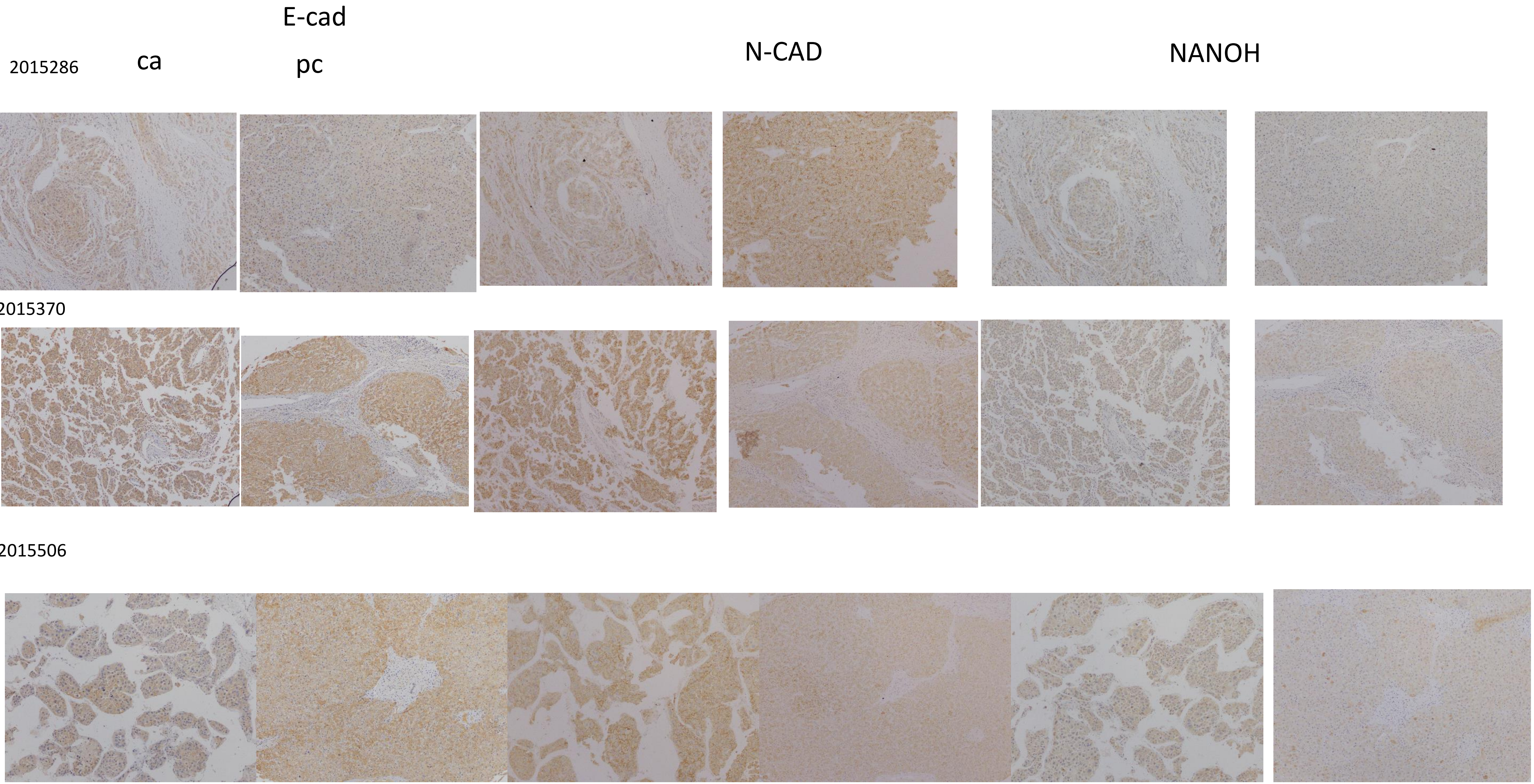
E-cad
2015286
ca
pc
N-CAD
NANOH
2015370
2015506

E-cad

2015605 ca pc N-CAD NANOH

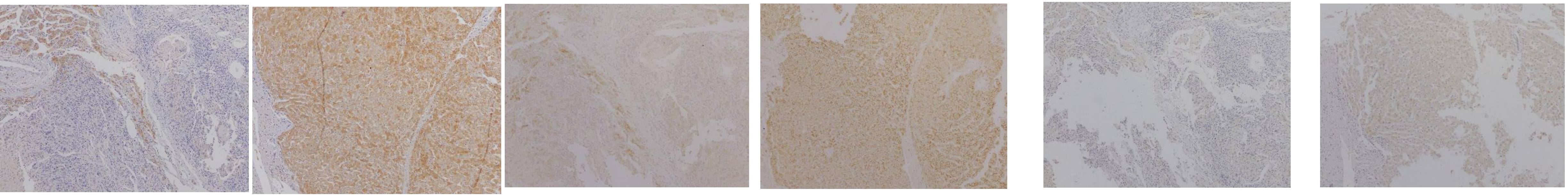

2015626

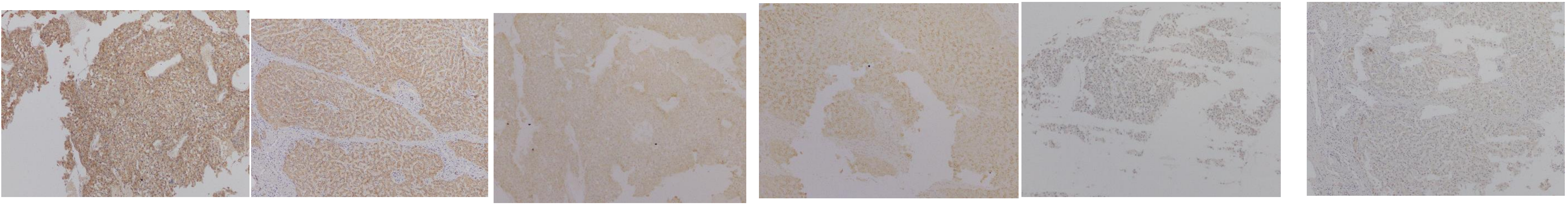

2015631

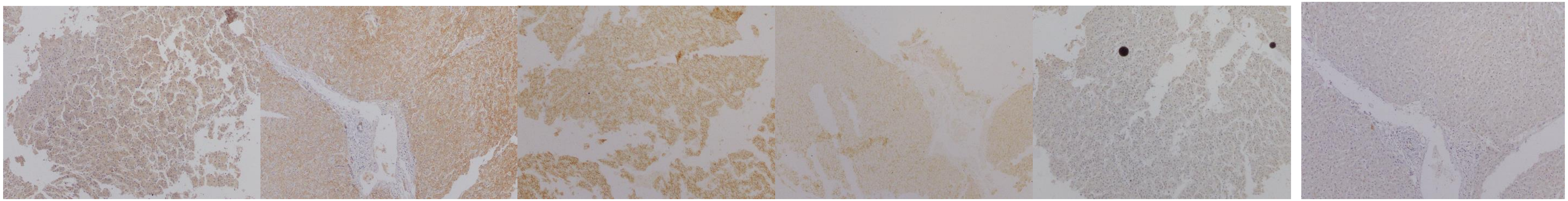

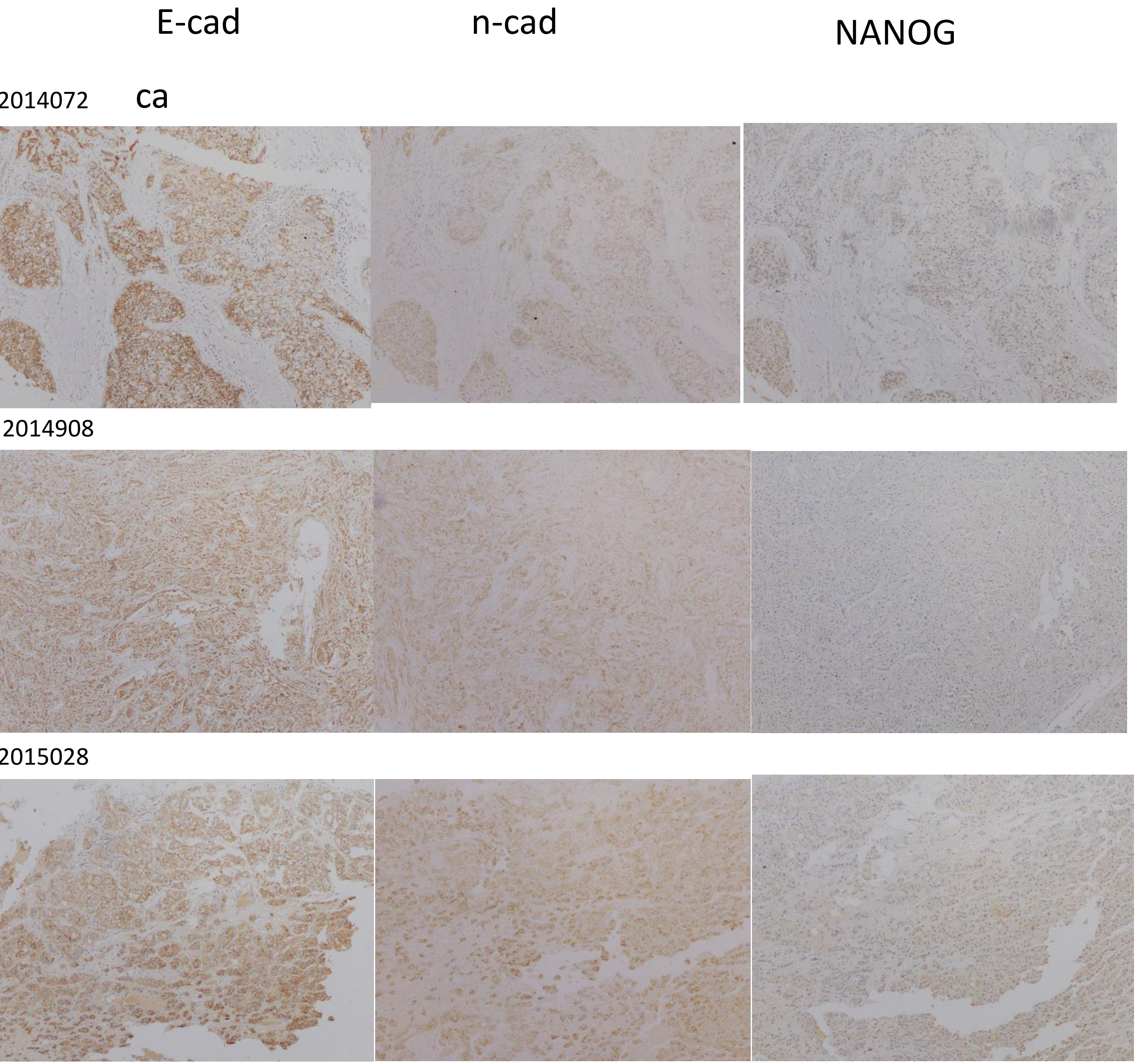
E-cad
n-cad
NANOG
2014072
ca
2014908
2015028

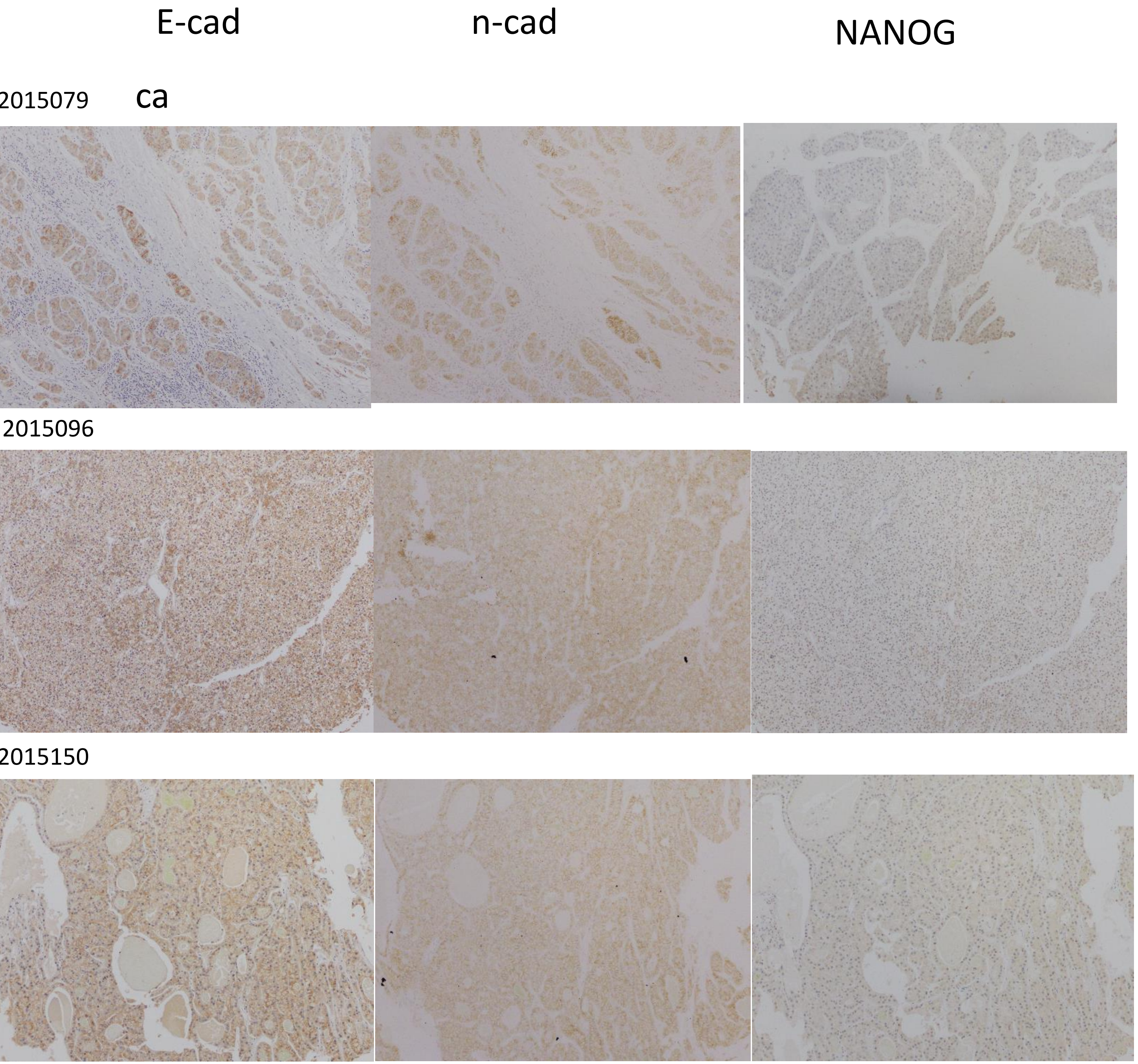
E-cad
n-cad
NANOG
2015079
ca
2015096
2015150

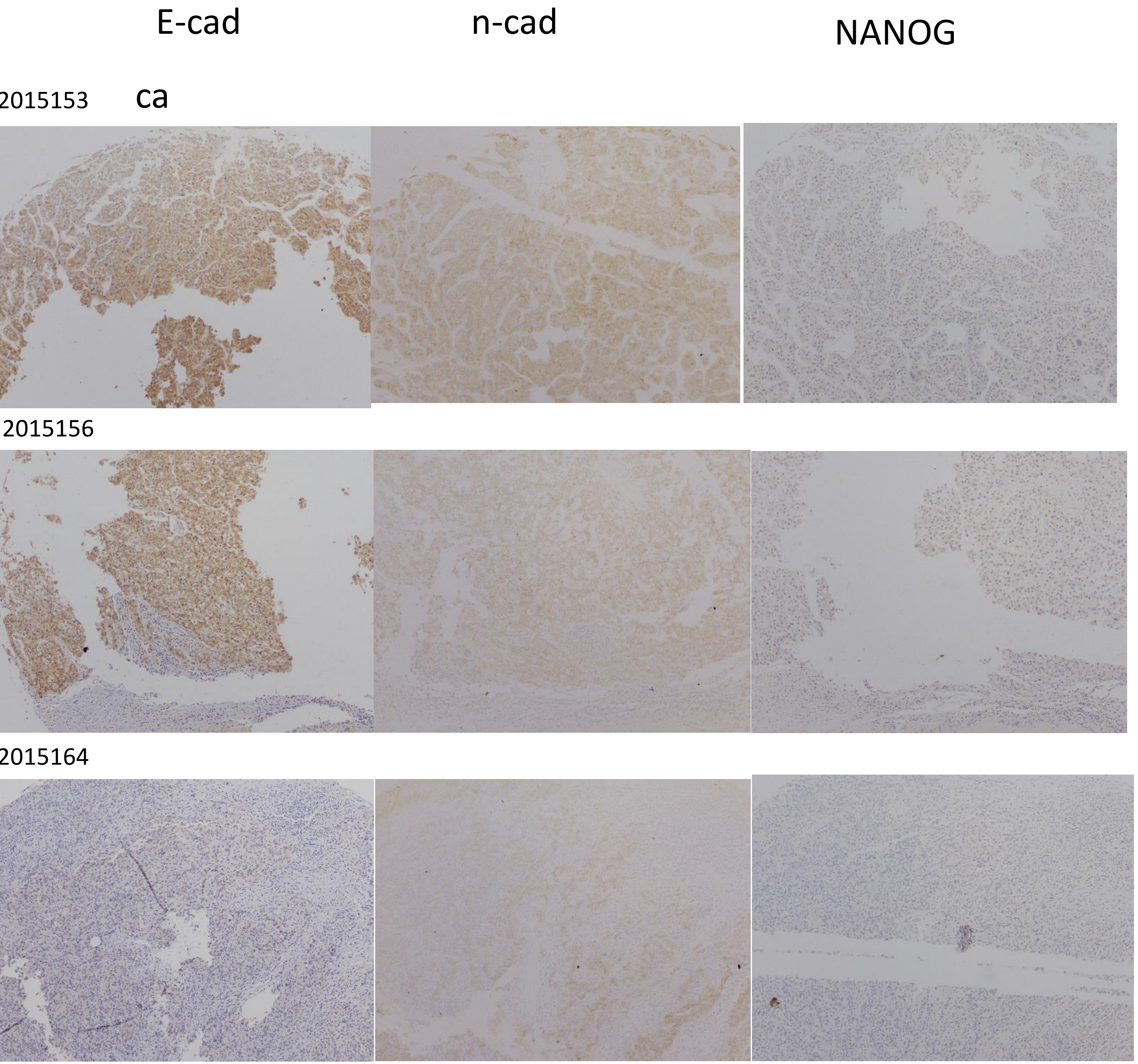
E-cad
n-cad
NANOG
2015153
ca
2015156
2015164

E-cad n-cad NANOG

2015213 ca

2015237

2015276

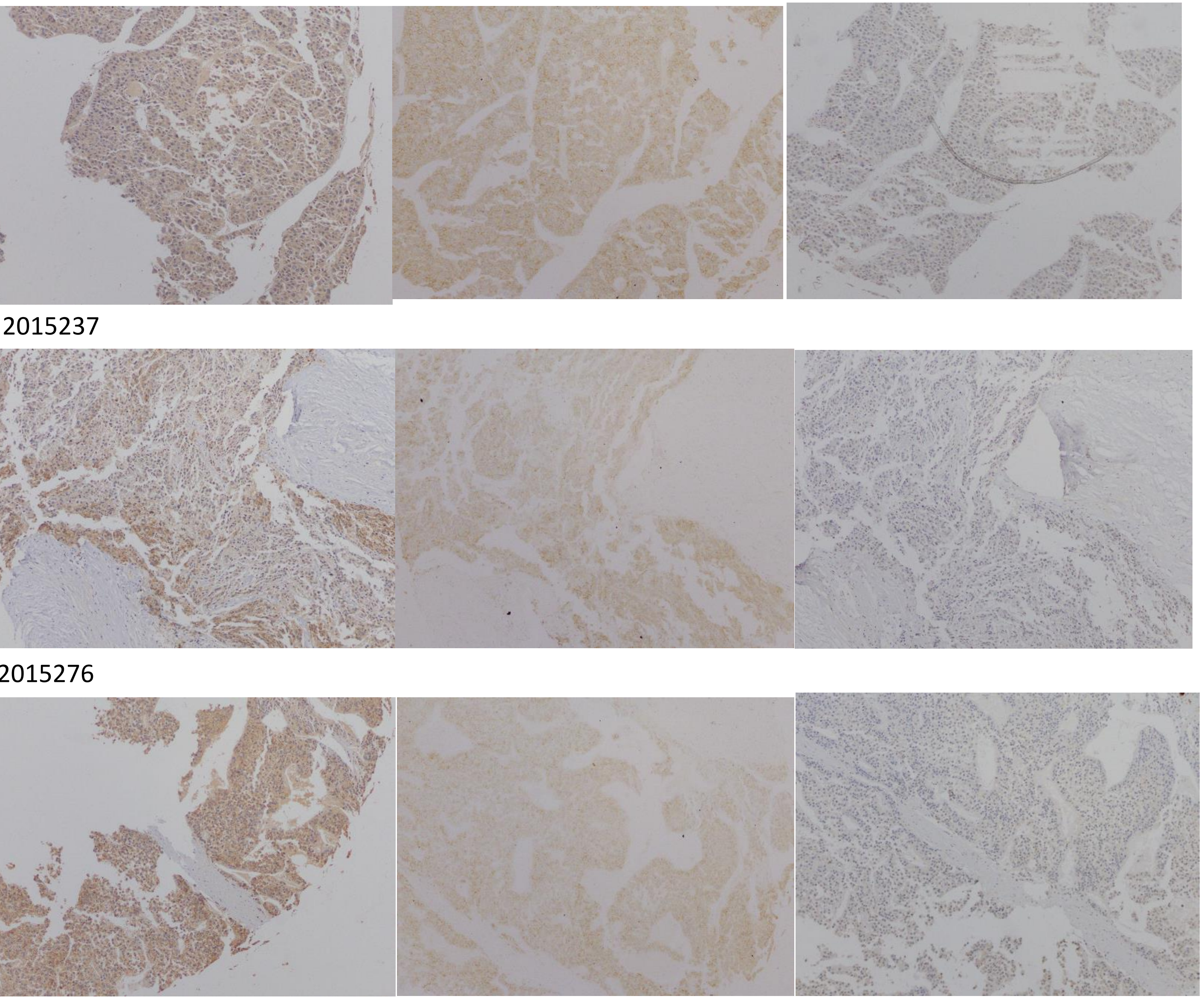

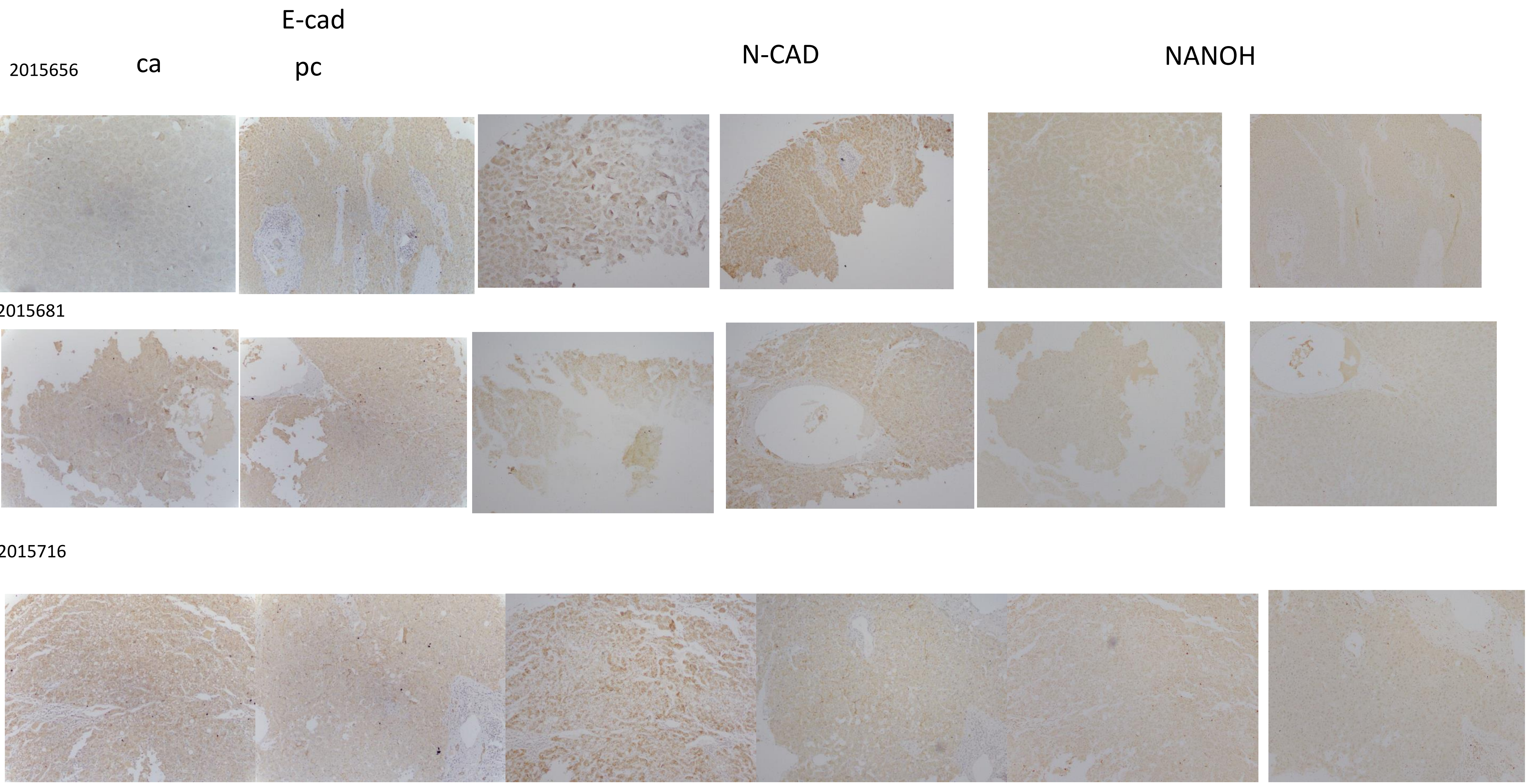
E-cad
2015656
ca
pc
N-CAD
NANOH
2015681
2015716

E-cad

N-CAD

NANOH

2014102

ca

pc

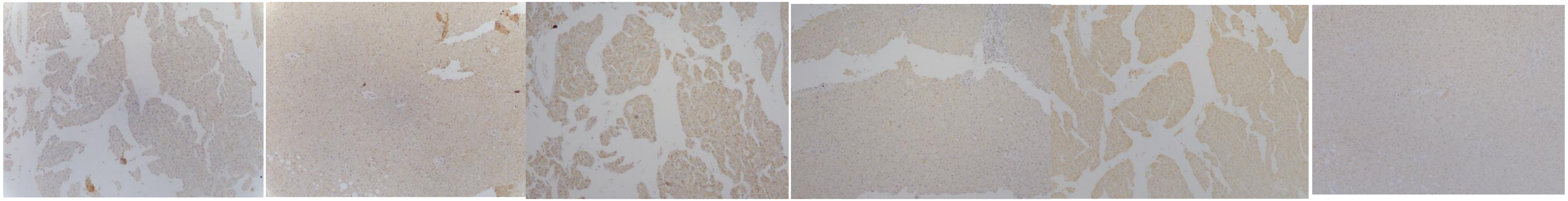

2016004

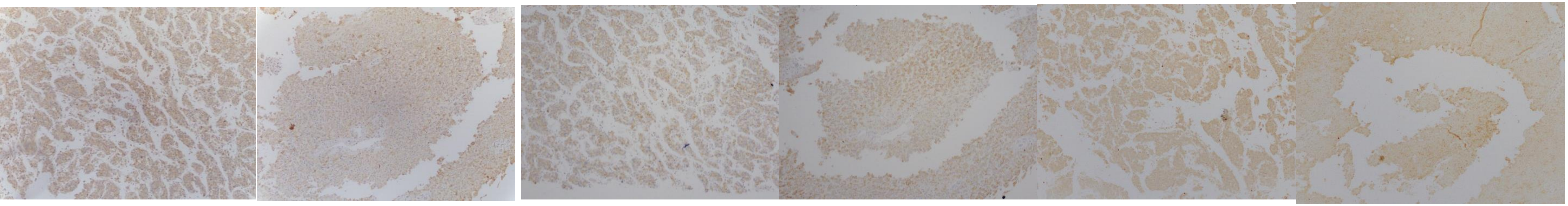

2016015

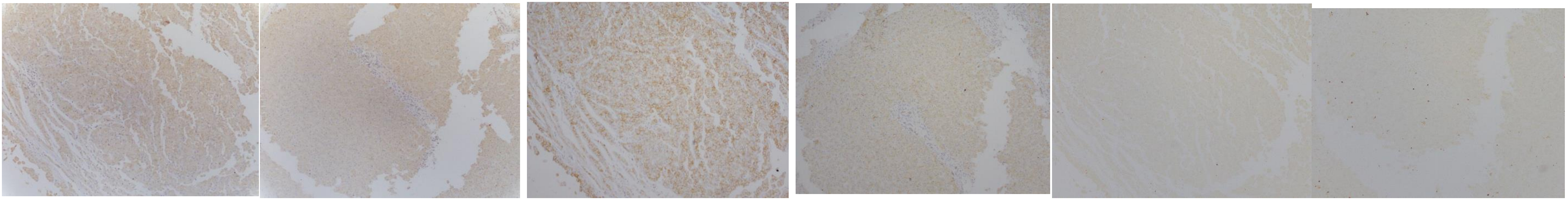

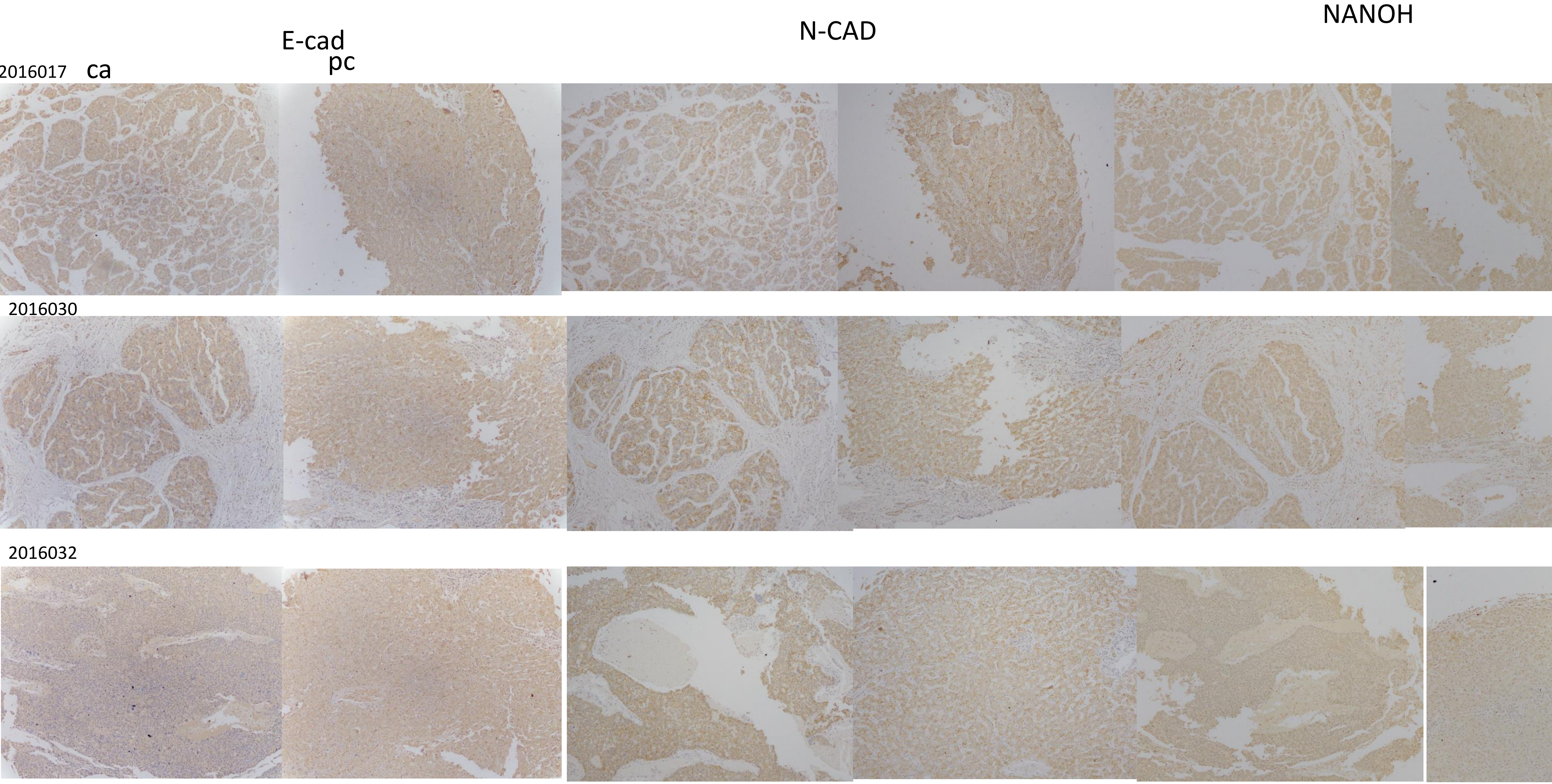
E-cad
N-CAD
NANOH
2016017
ca
pc
2016030
2016032

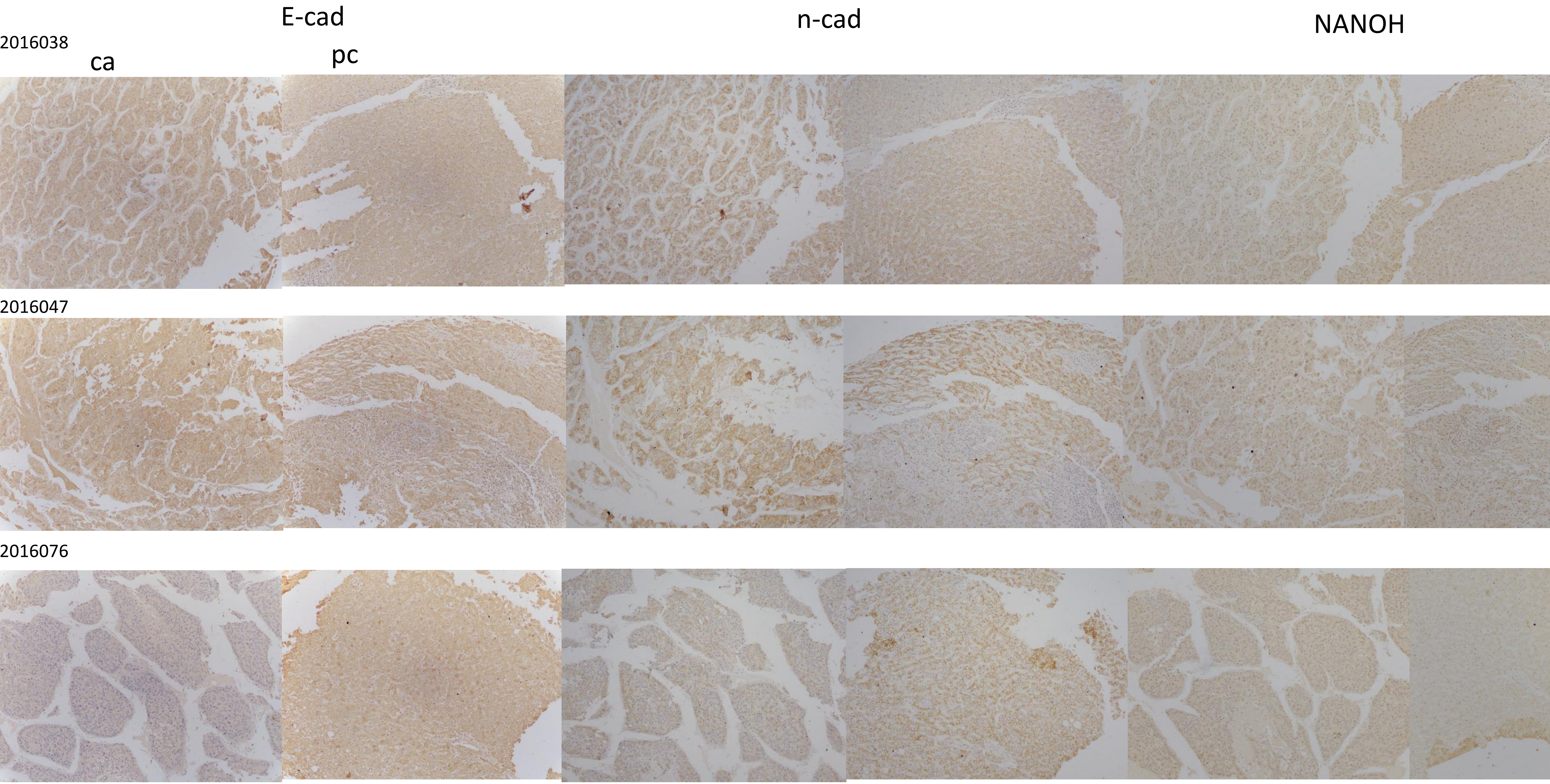
E-cad
n-cad
NANOH
2016038
ca
pc
2016047
2016076

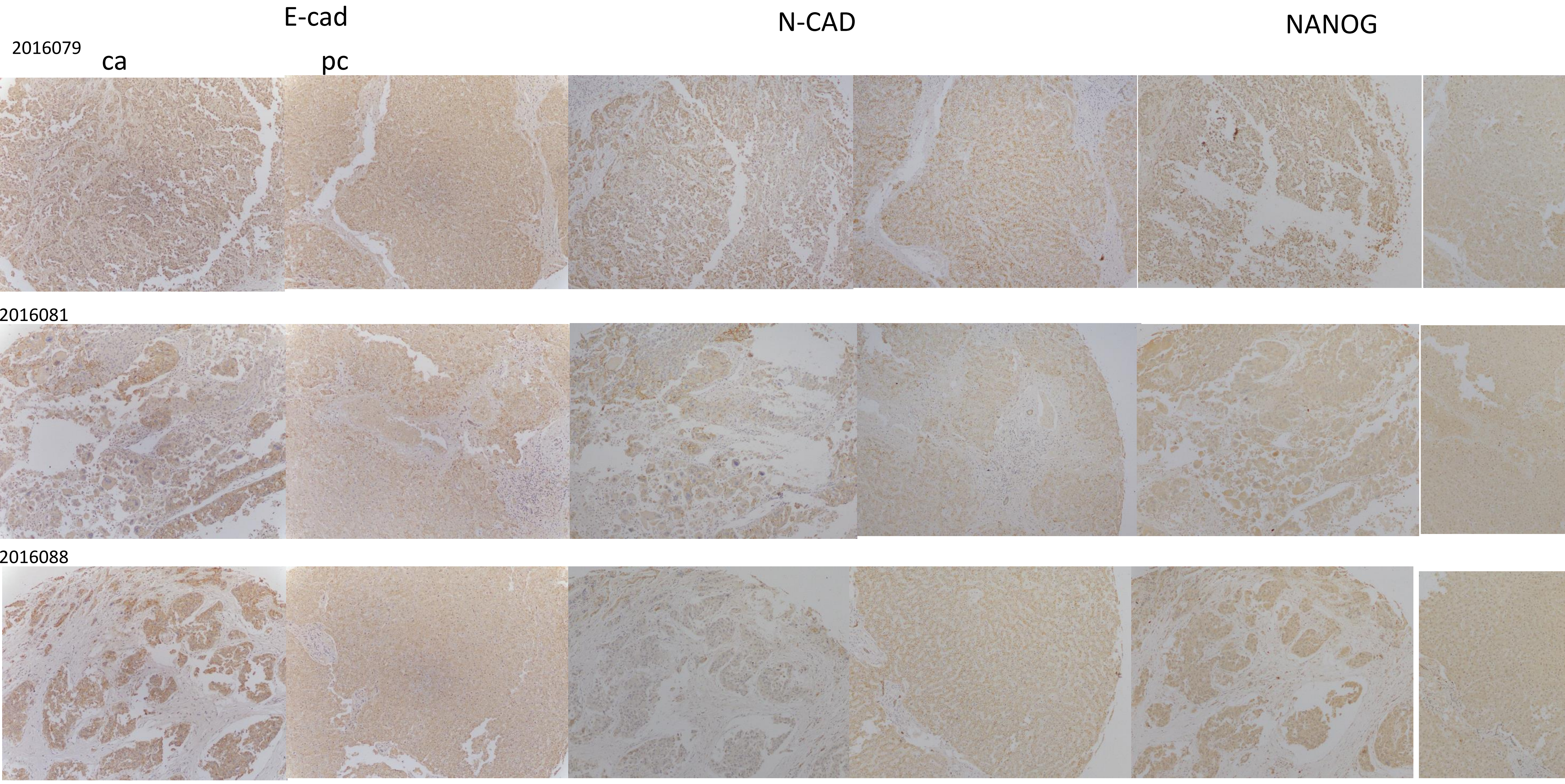
E-cad
N-CAD
NANOG
2016079
ca
pc
2016081
2016088

E-ca[illegible]c

ca

2016097

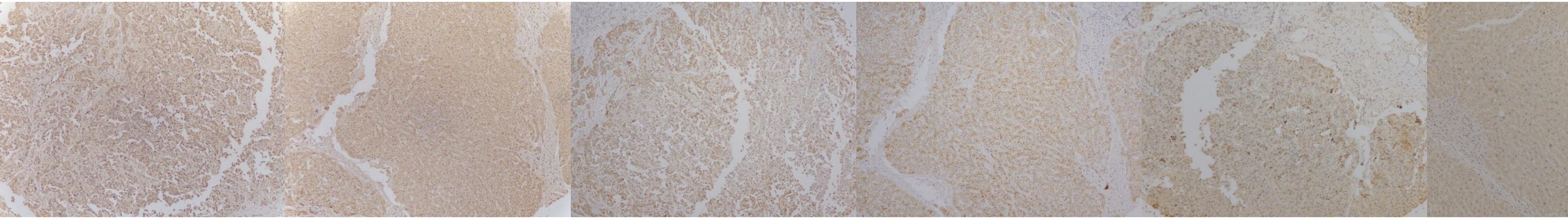

2016126

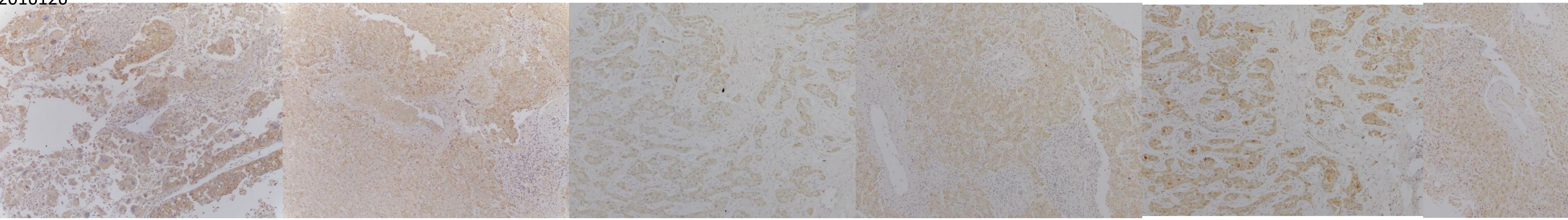

2016160

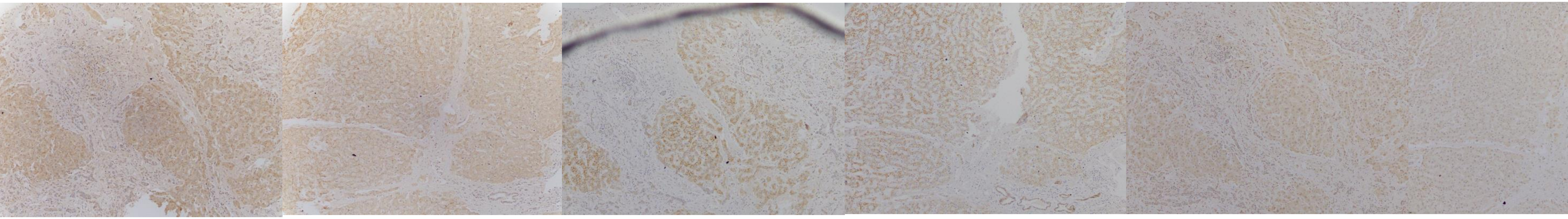

E-cad

2016131 ca pc

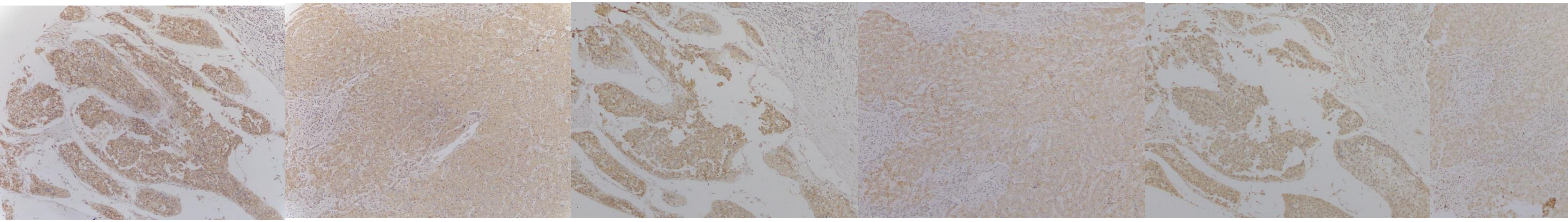

2016132

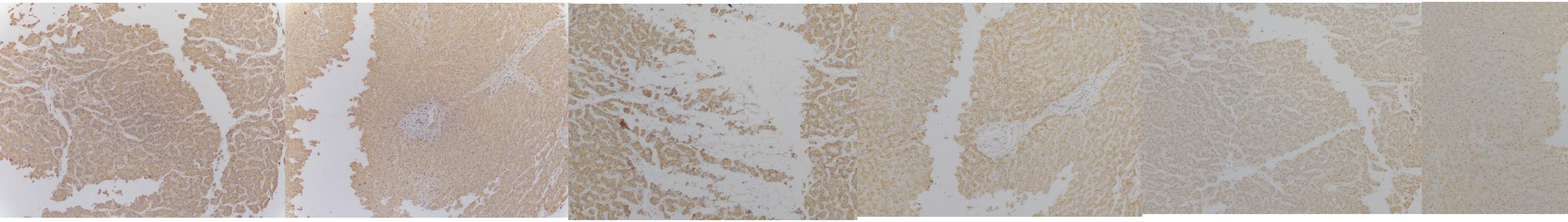

2016140

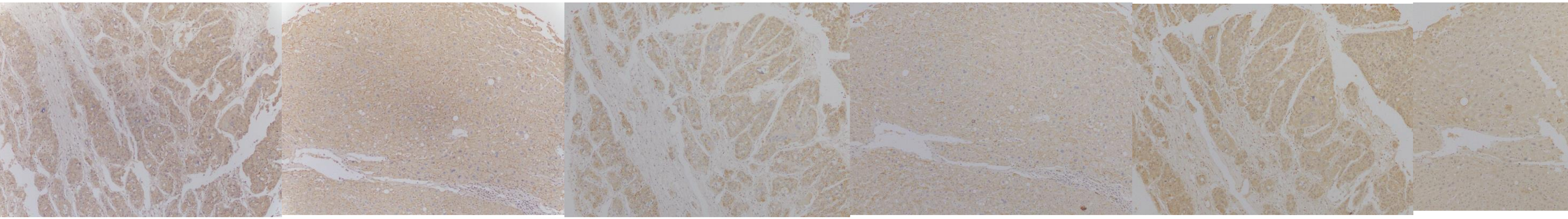

E-cad

ca pc

2016161

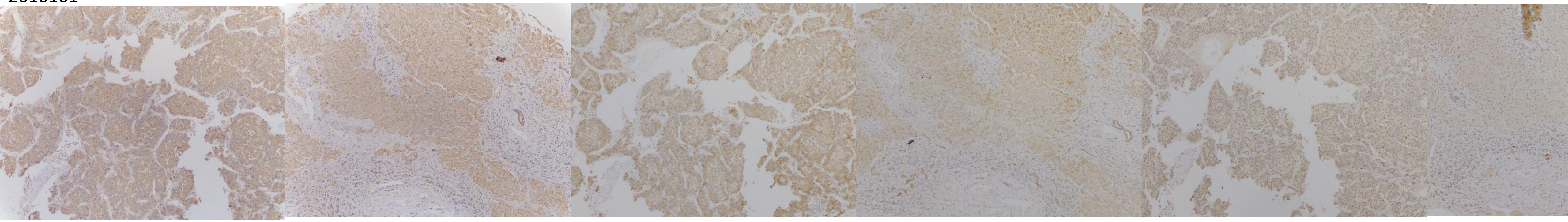

2016165

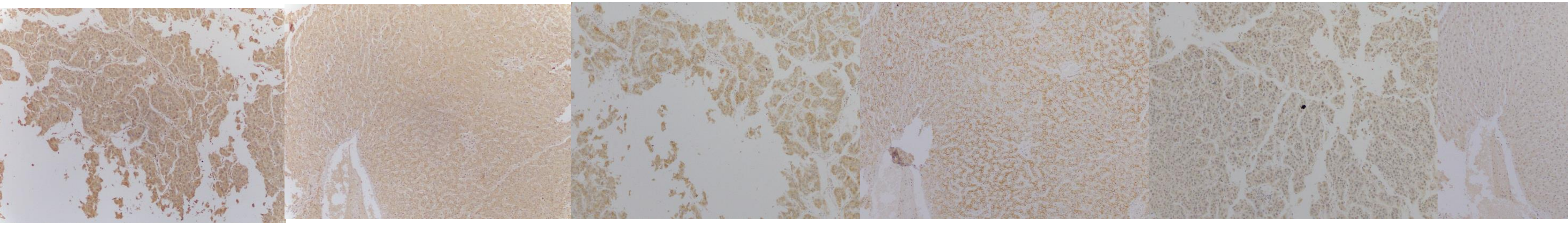

2016201

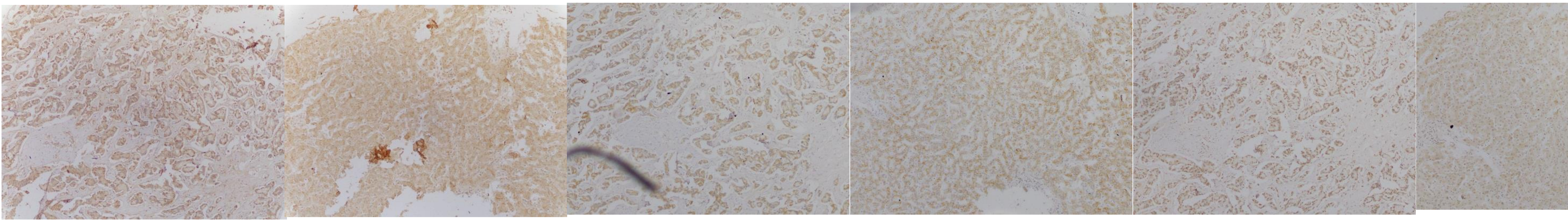

E-cad

ca pc

2016221

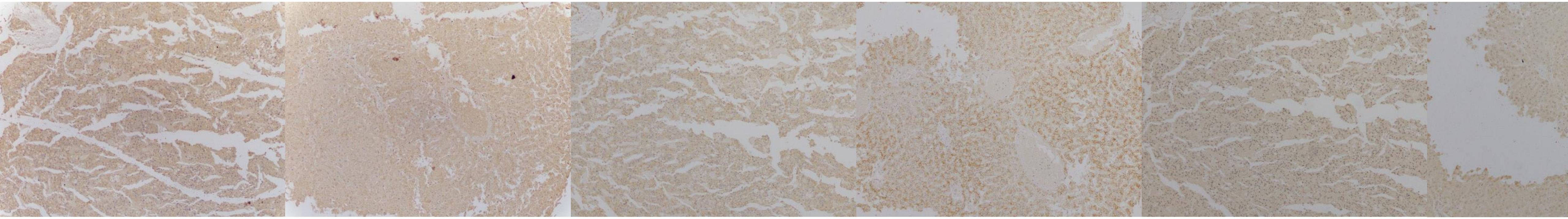

2016228

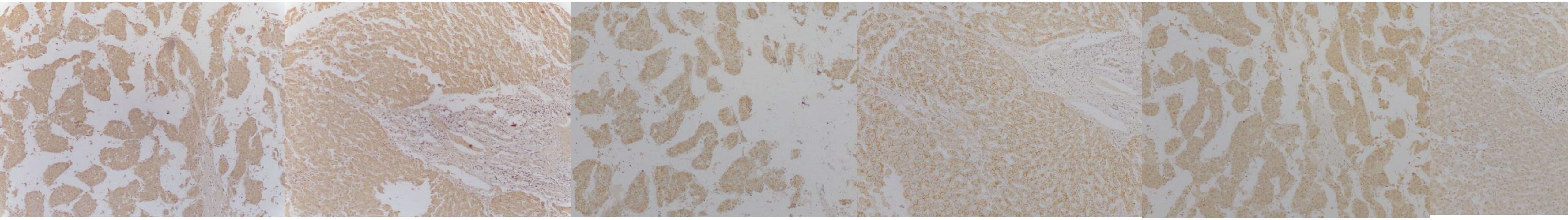

2016229

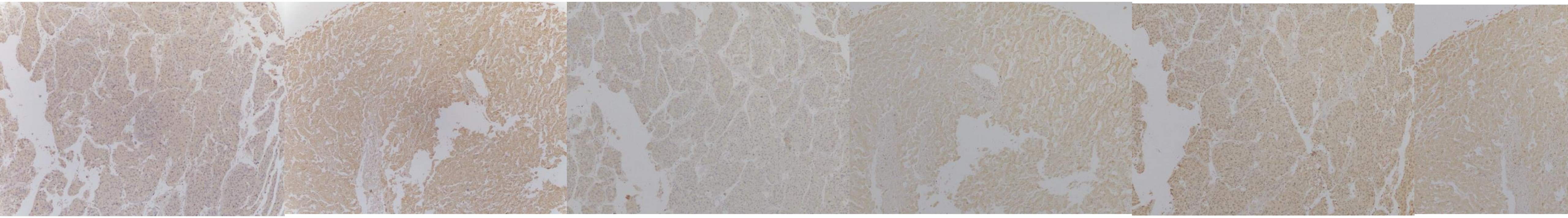

E-cad

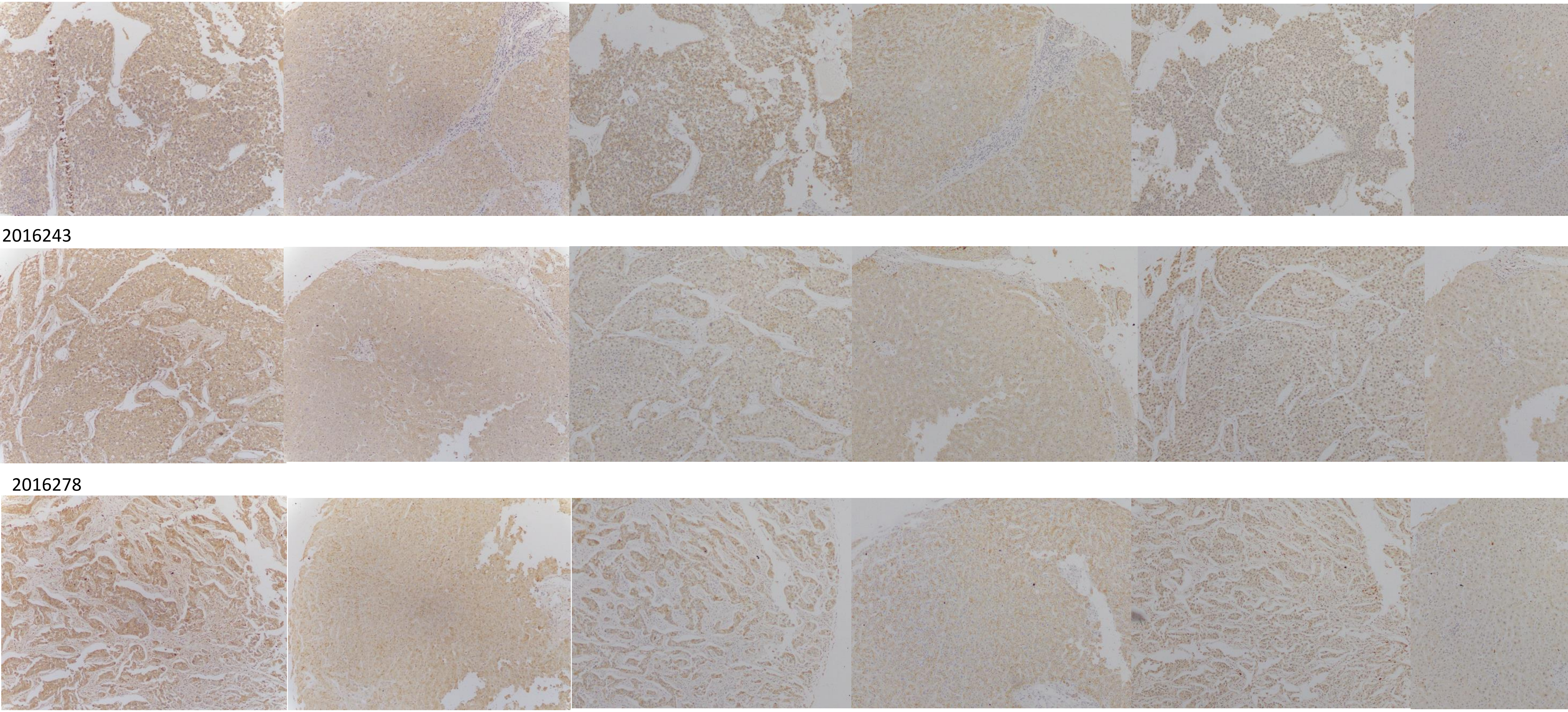

E-cad

ca pc

2016289

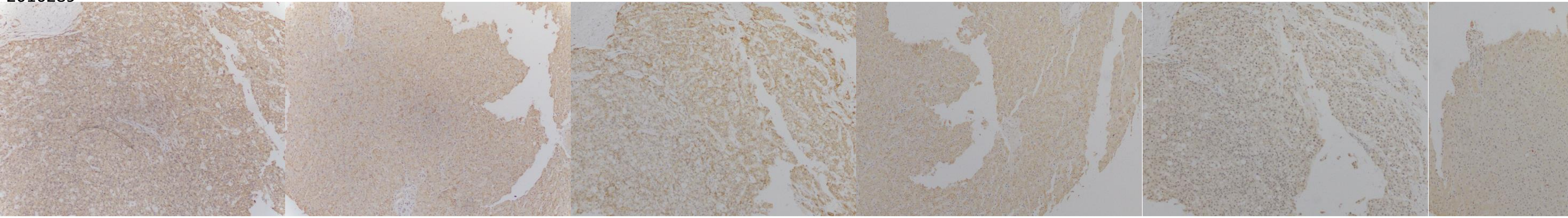

2016292

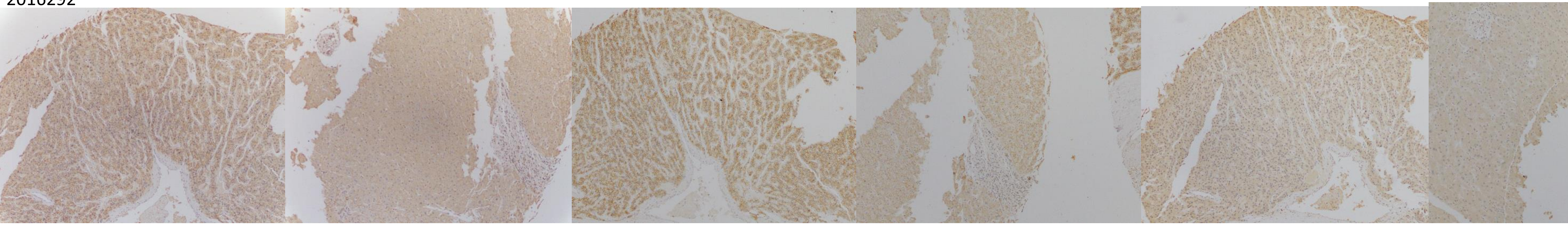

2016293

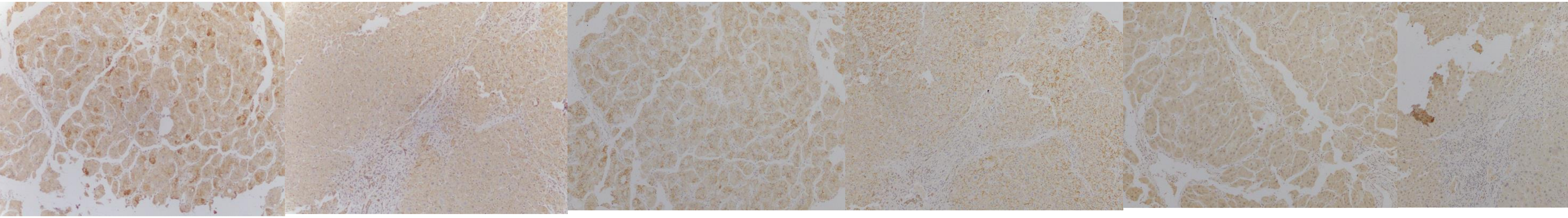

E-cad

ca pc

2016297

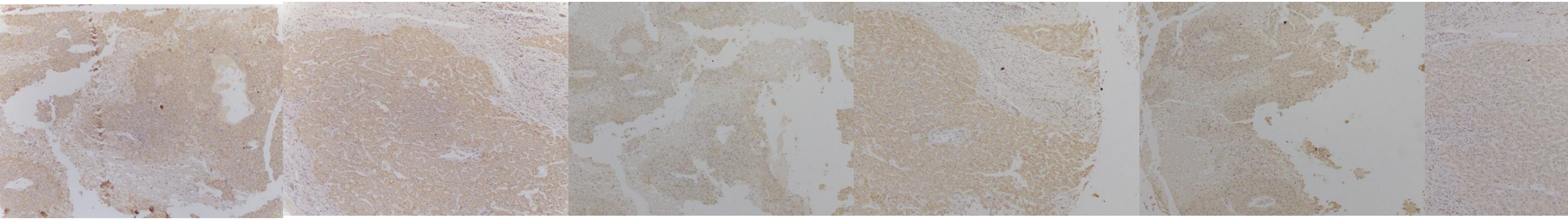

2016300

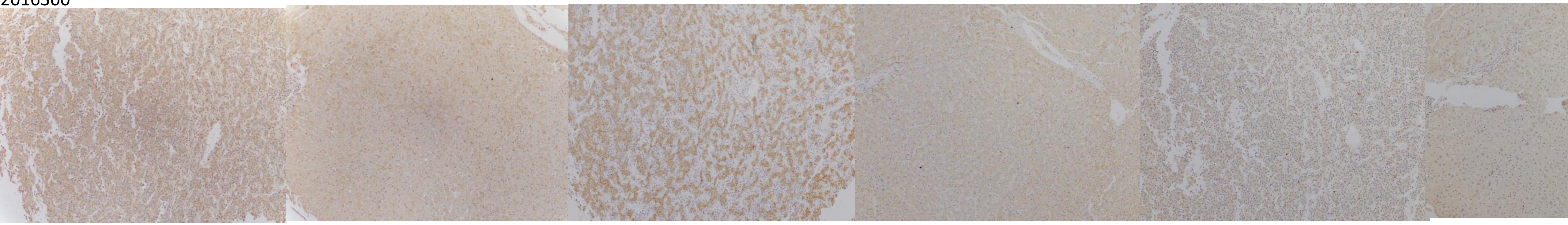

2016301

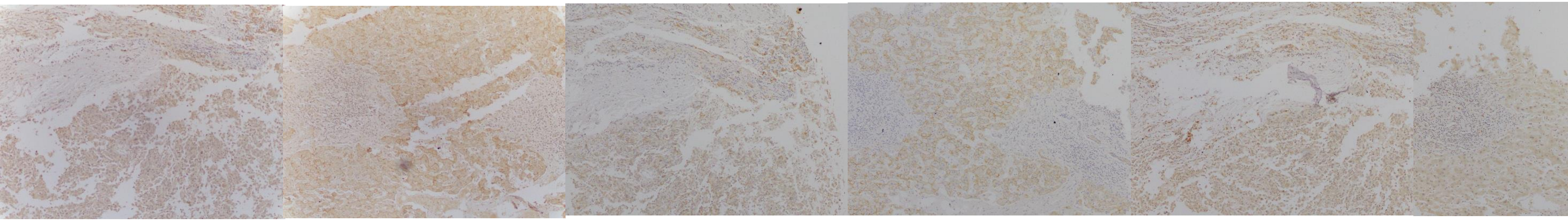

E-cad

ca pc

2016307

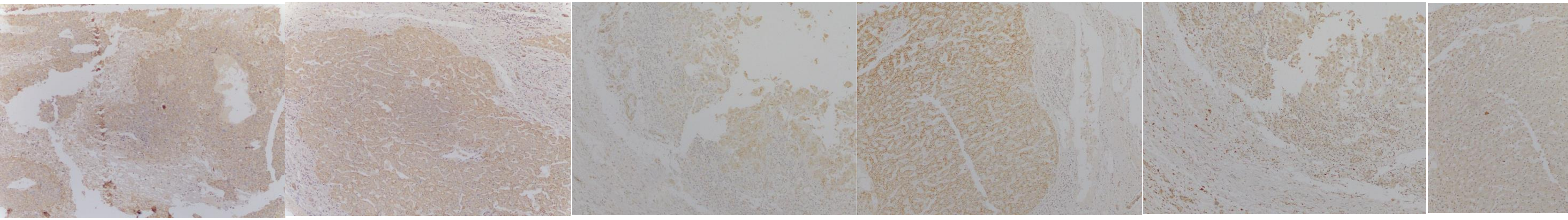

2016310

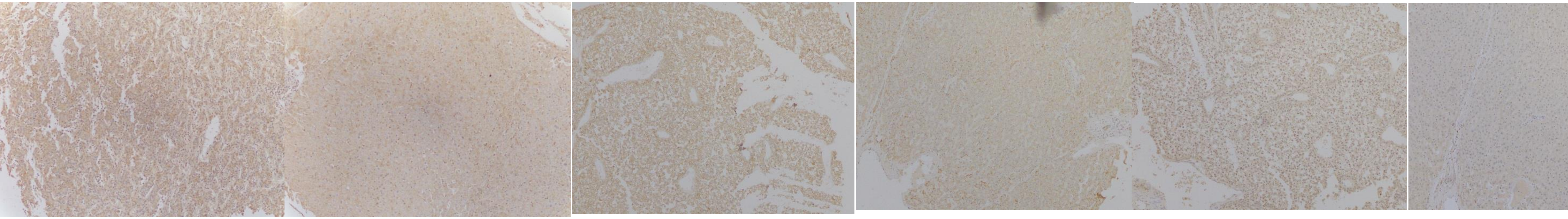

2016312

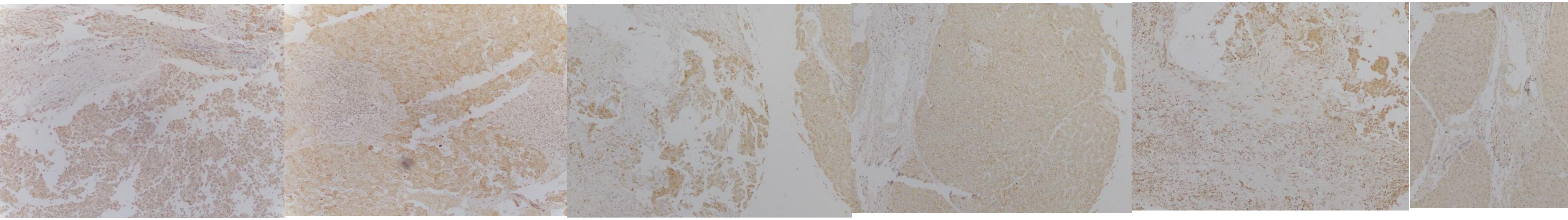

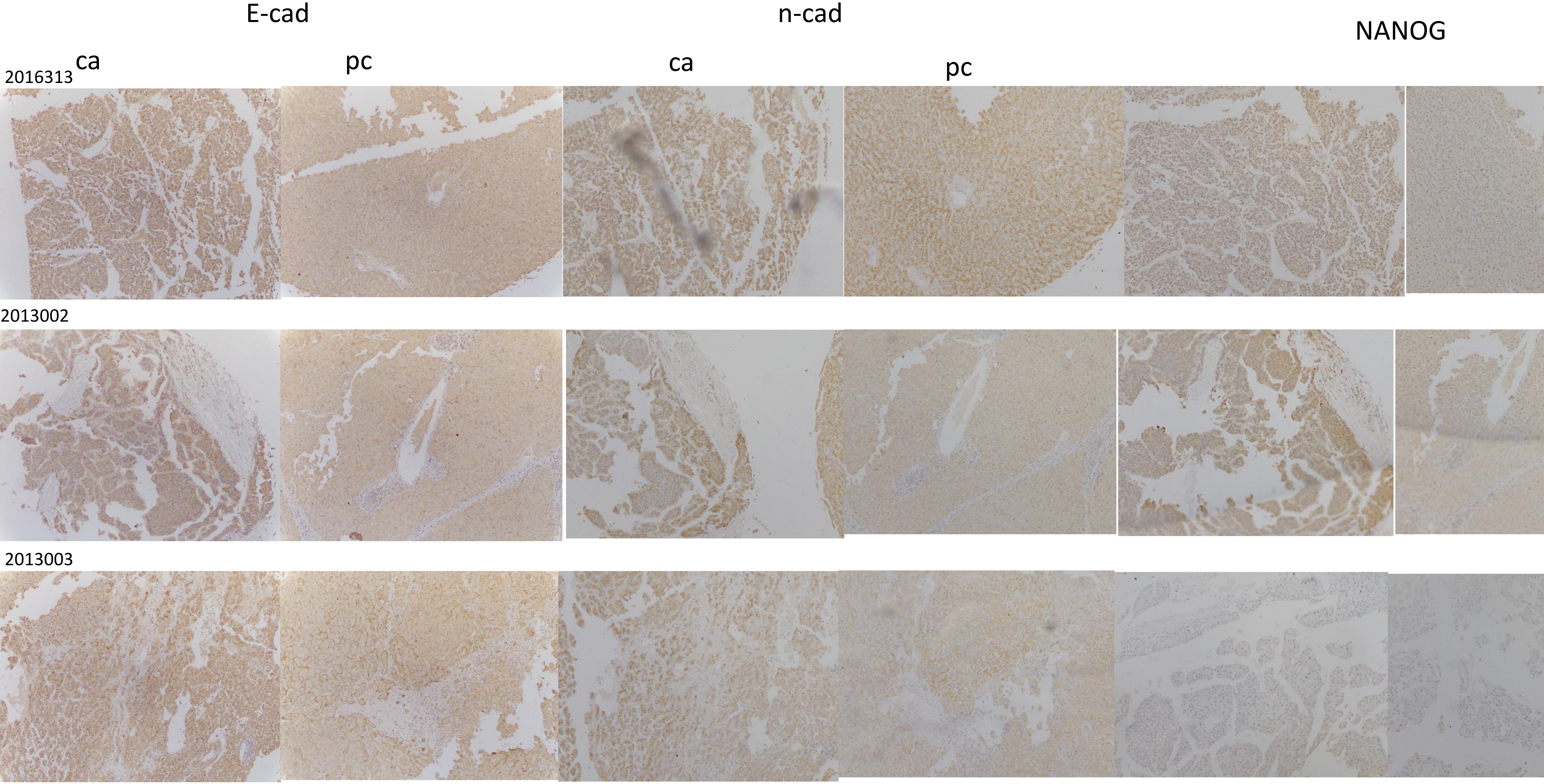
E-cad
n-cad
NANOG
ca
pc
ca
pc
2016313
2013002
2013003

E-cad n-cad NANOG

2015285 ca

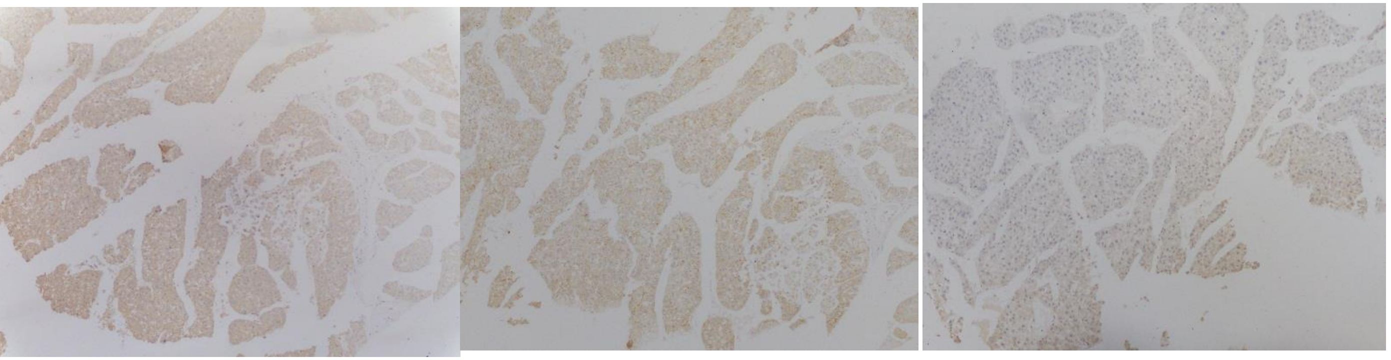

2015296

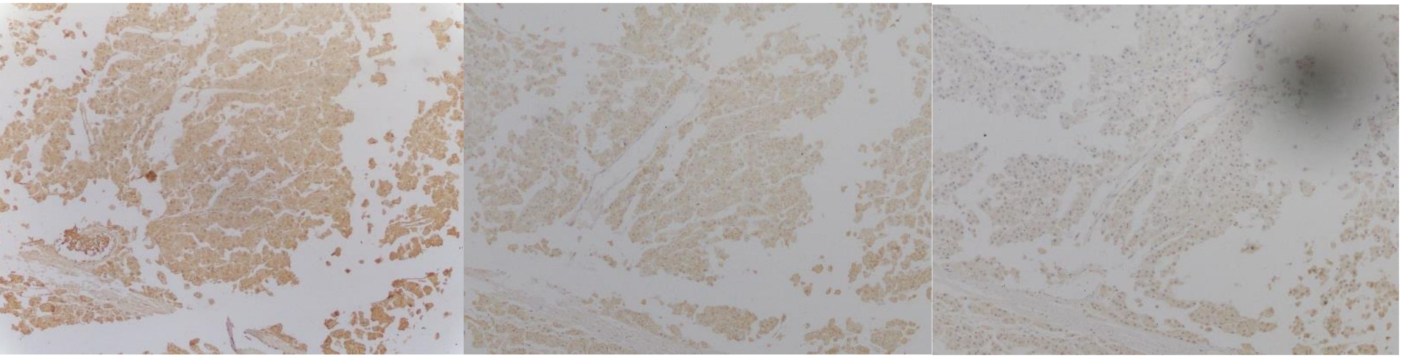

2015336

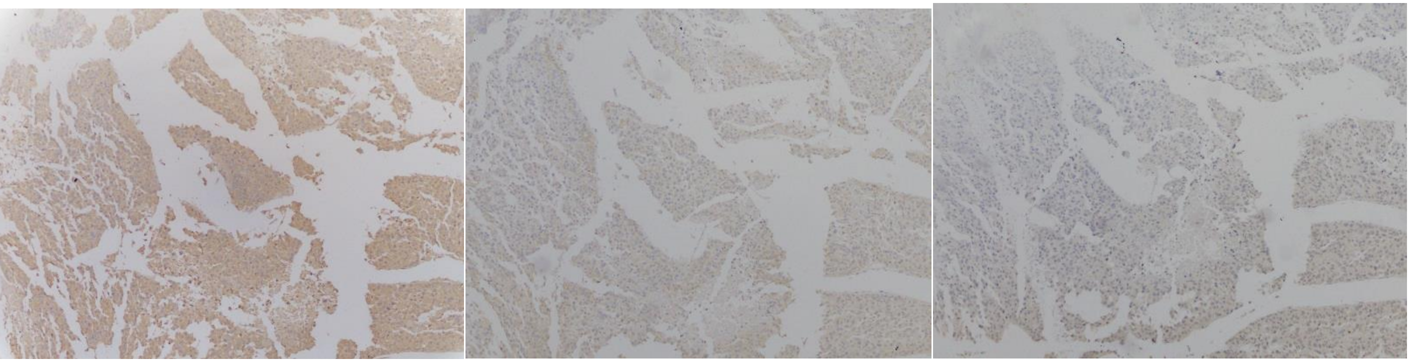

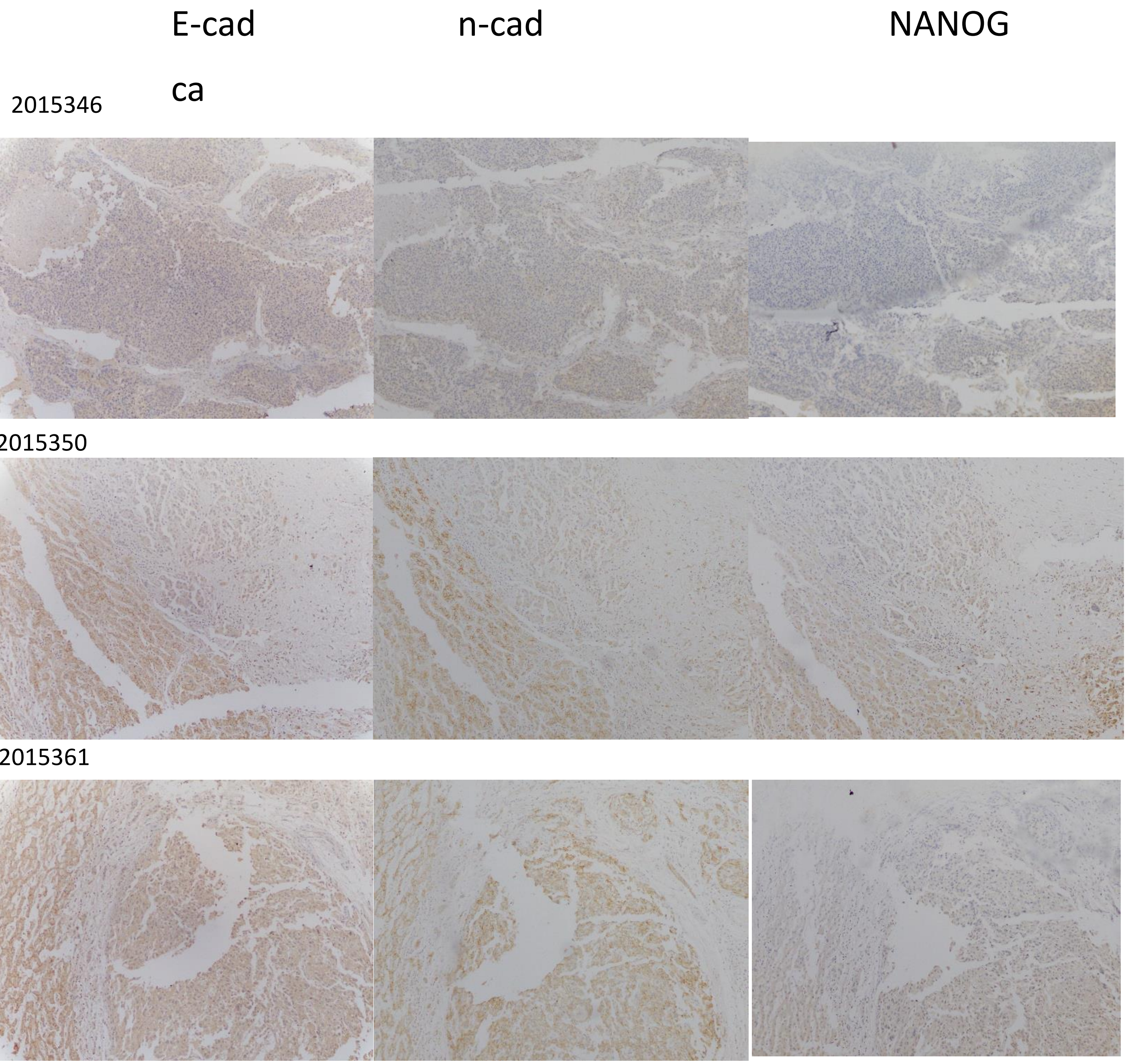
E-cad
ca
n-cad
NANOG
2015346
2015350
2015361

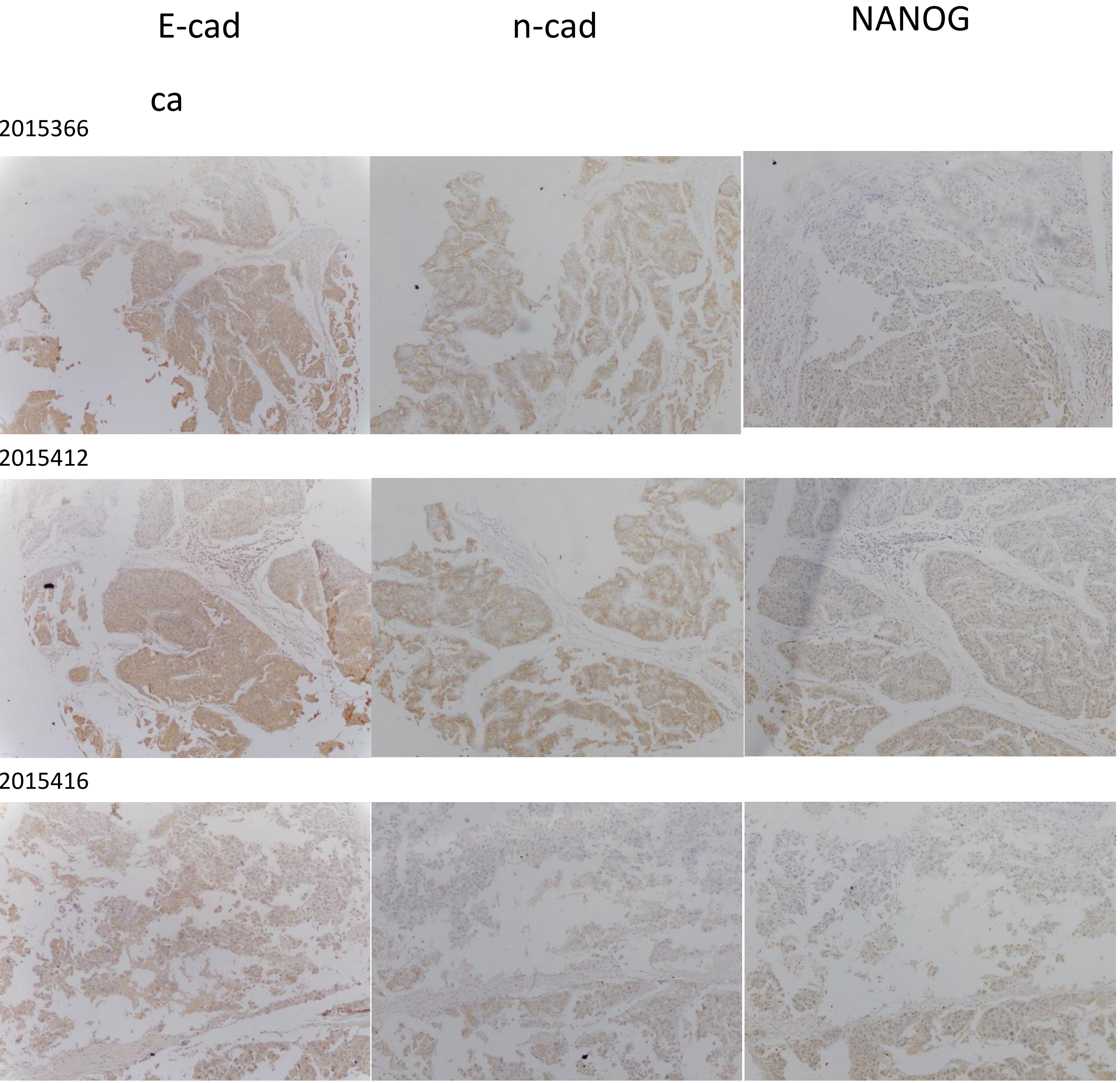
E-cad
n-cad
NANOG
ca
2015366
2015412
2015416

E-cad n-cad NANOG

ca

2015417

2015433

2015447

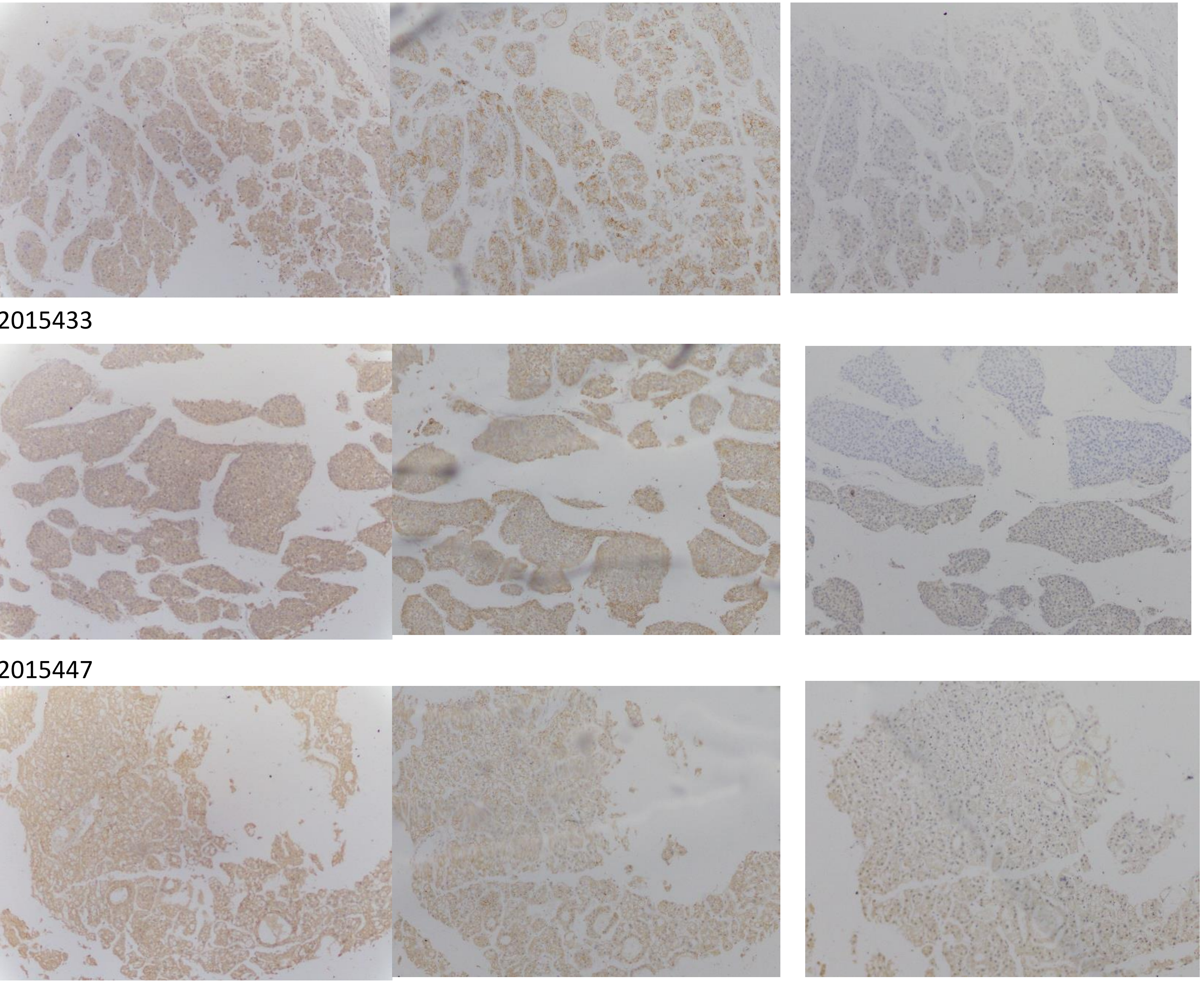

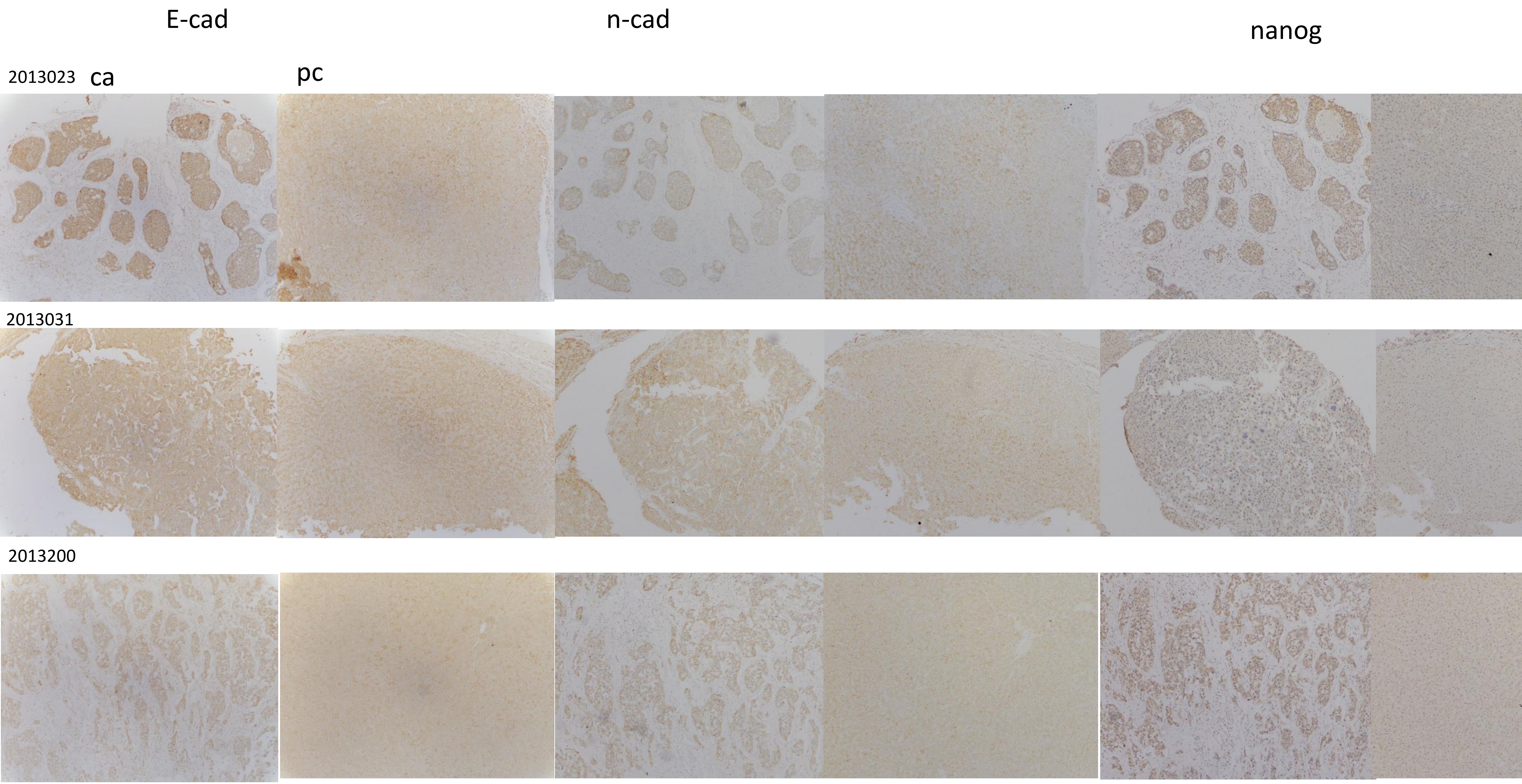
E-cad
n-cad
nanog
2013023
ca
pc
2013031
2013200

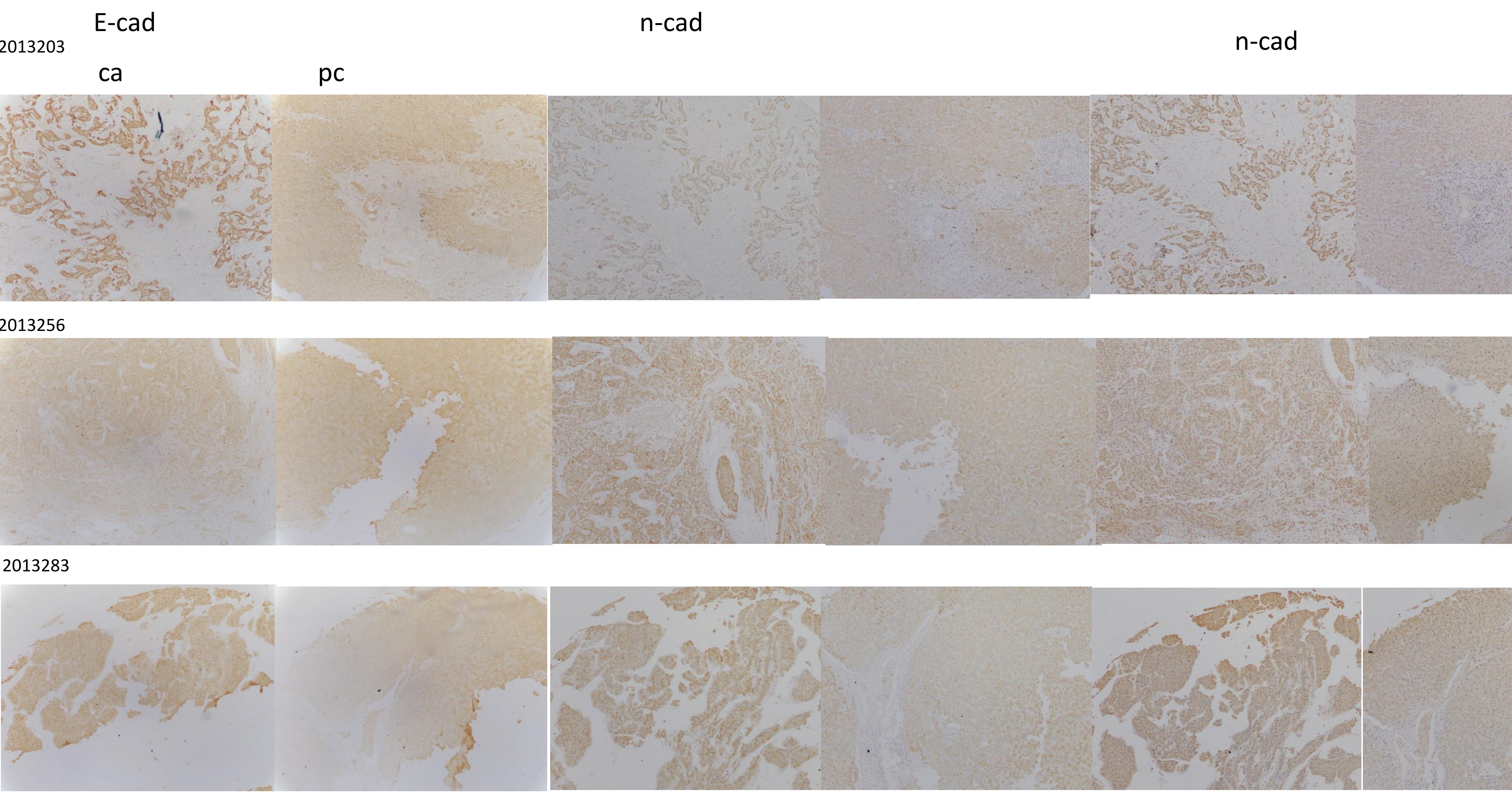
E-cad
n-cad
n-cad
2013203
ca
pc
2013256
2013283

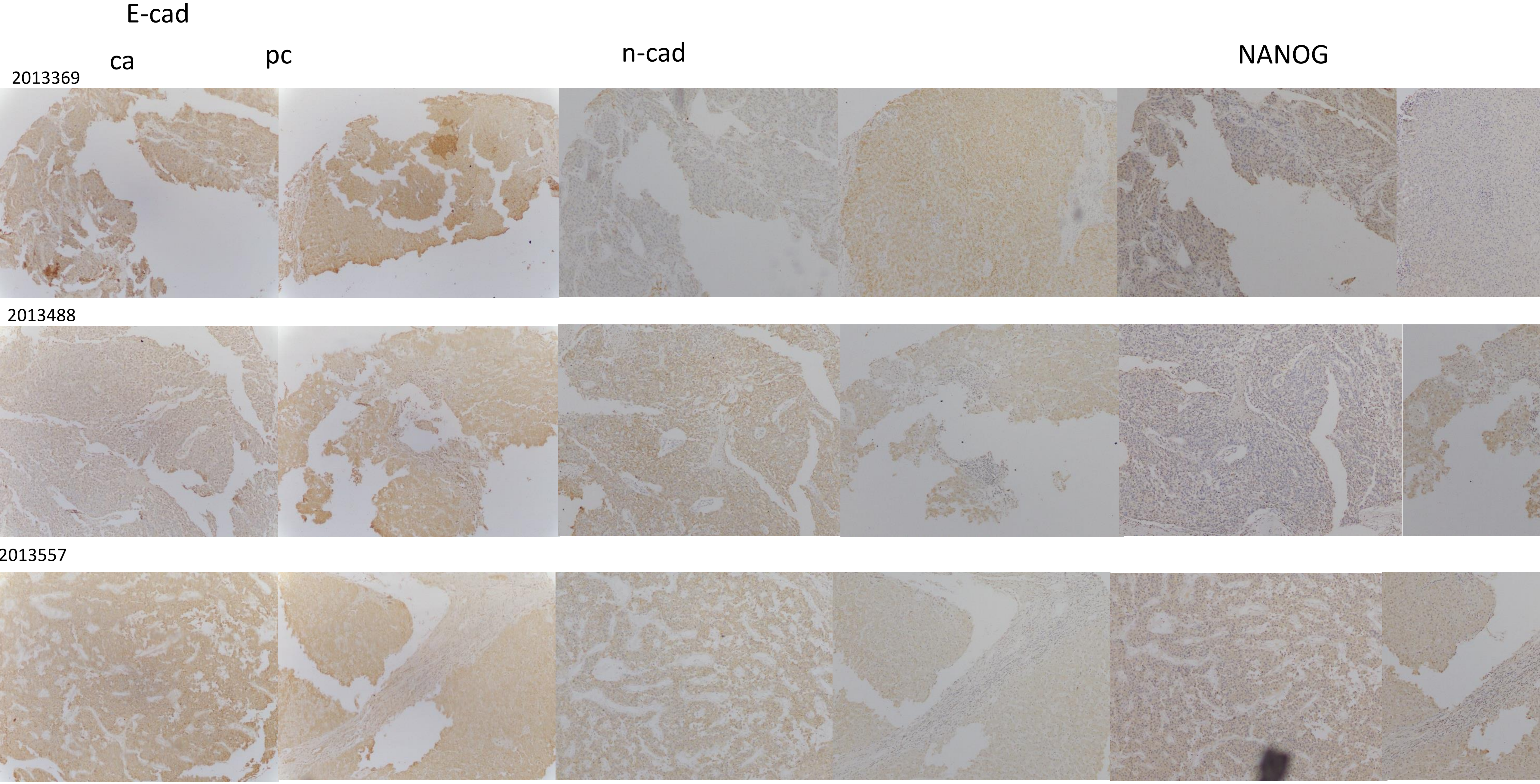
E-cad
ca
pc
n-cad
NANOG
2013369
2013488
2013557

E-cad

ca pc n-cad NANOG

2013571

2013852

2013867

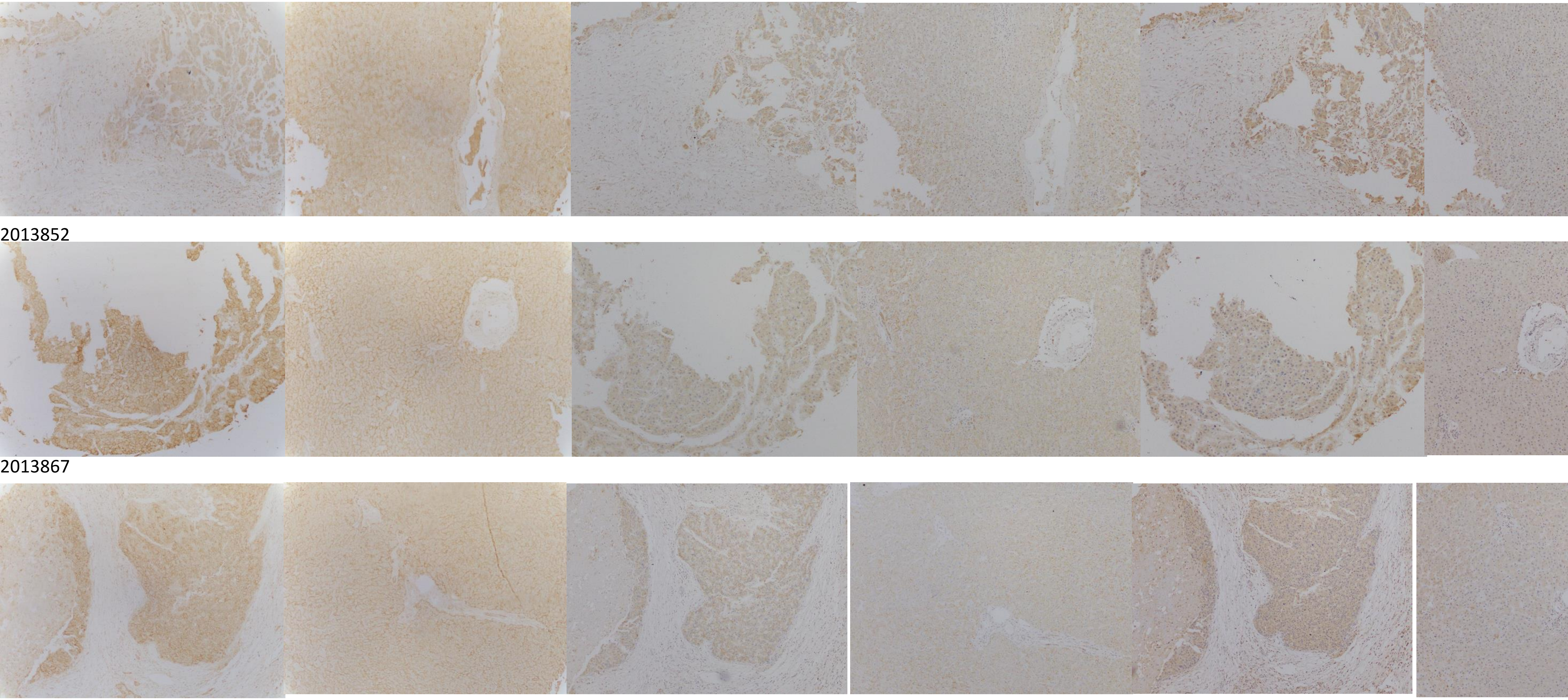

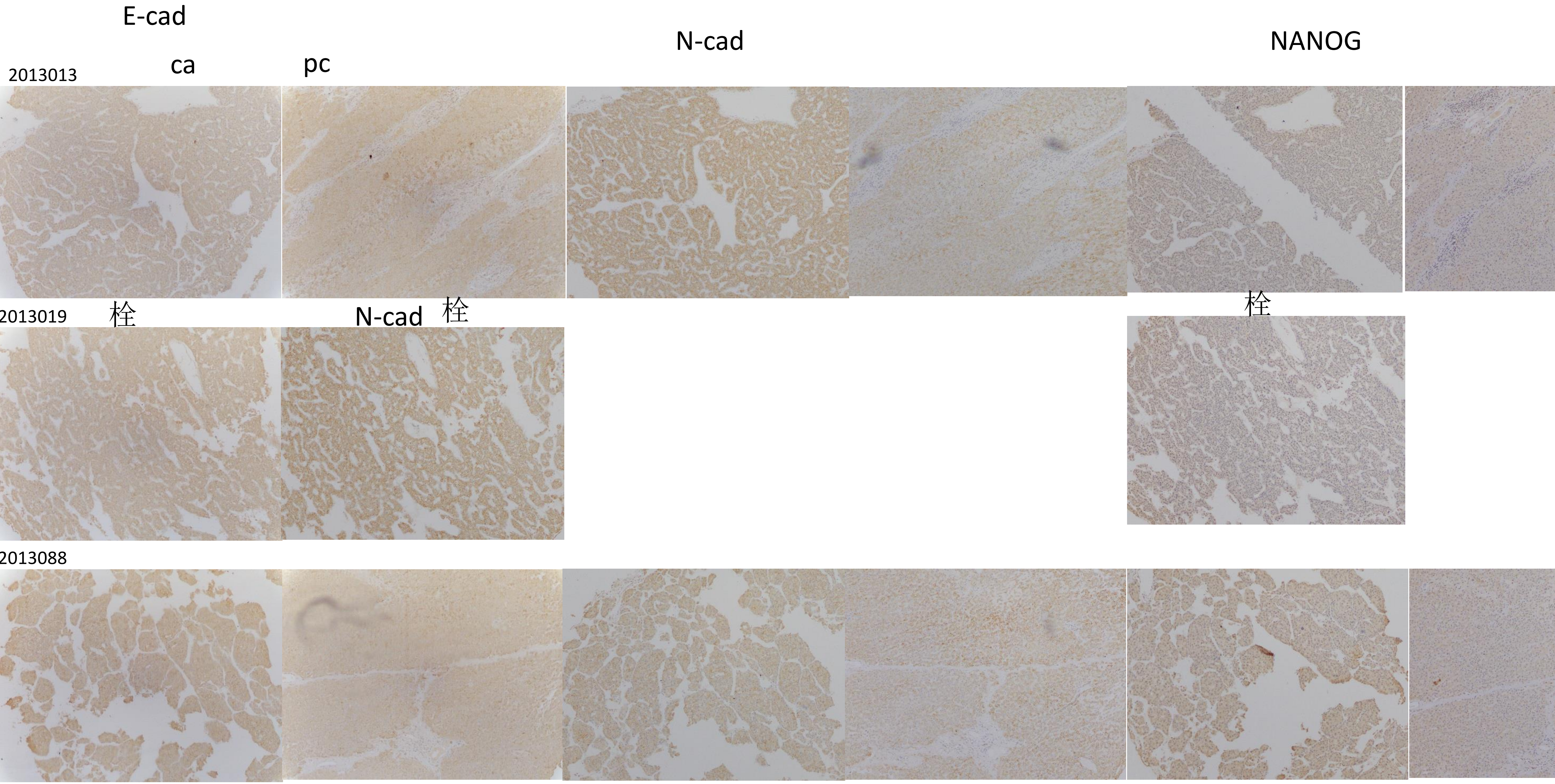

E-cad
ca
pc
N-cad
NANOG
2013013
2013019
栓
N-cad
栓
栓
2013088

2013088栓 E-cad N-cad NANOG

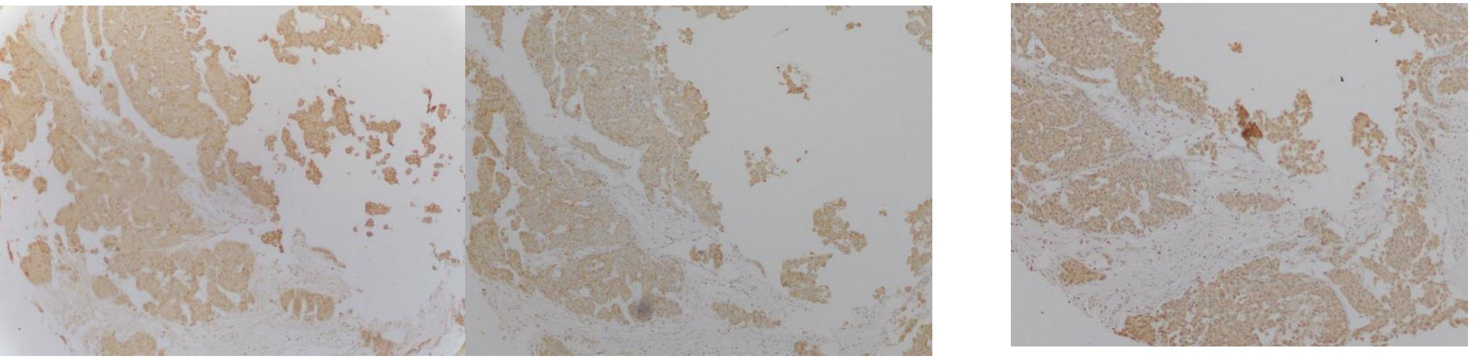

2015539 ca N-cad

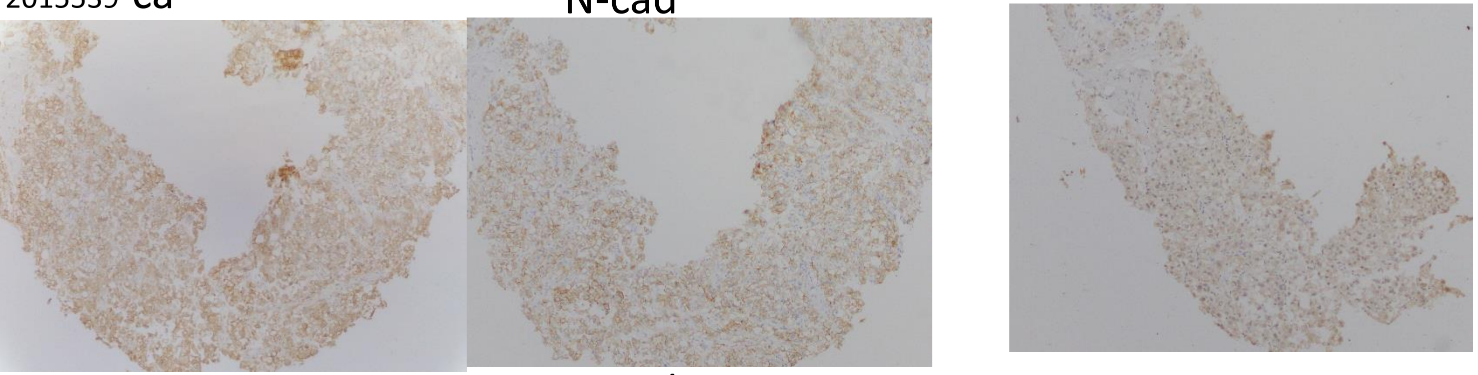

2015557 ca N-cad

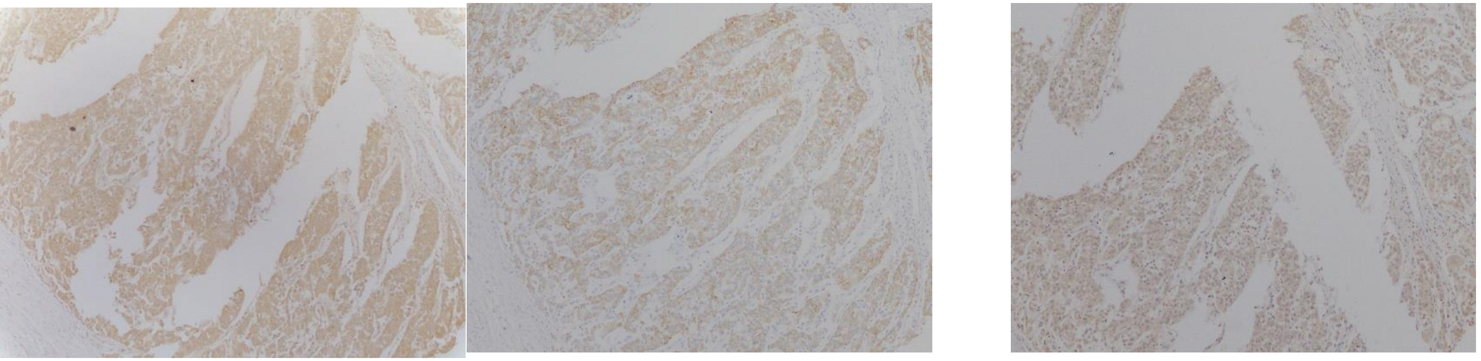

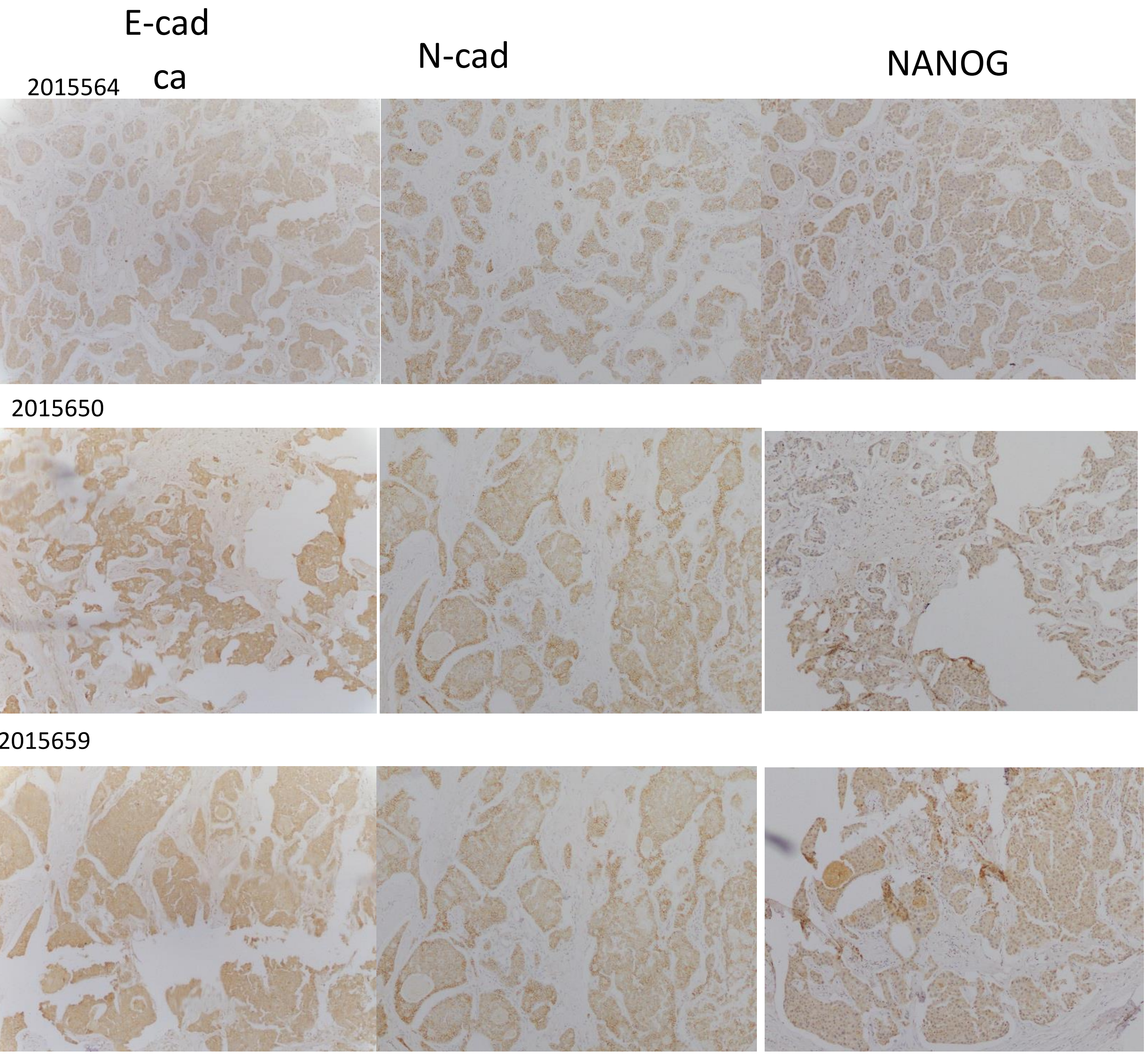
E-cad
ca
N-cad
NANOG
2015564
2015650
2015659

E-cad n-cad NANOG

2016028 ca

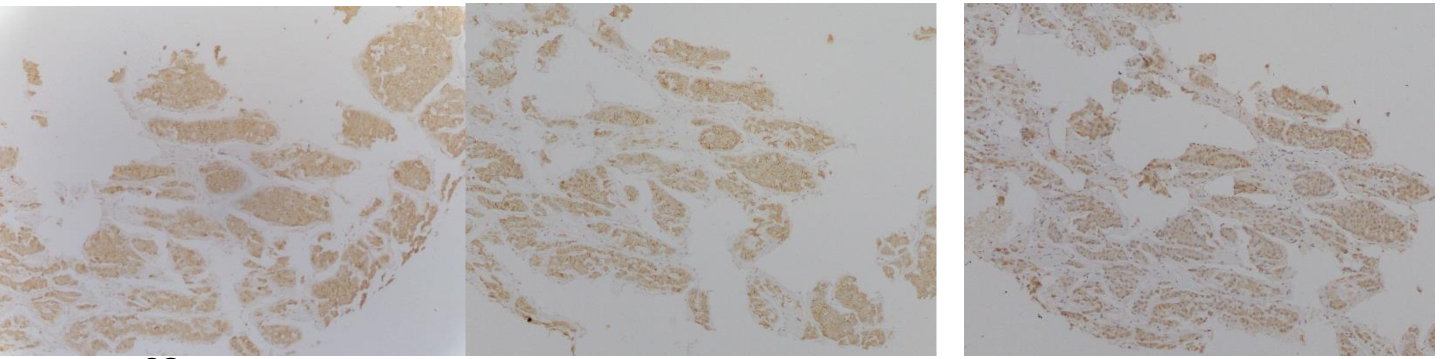

2016182 ca

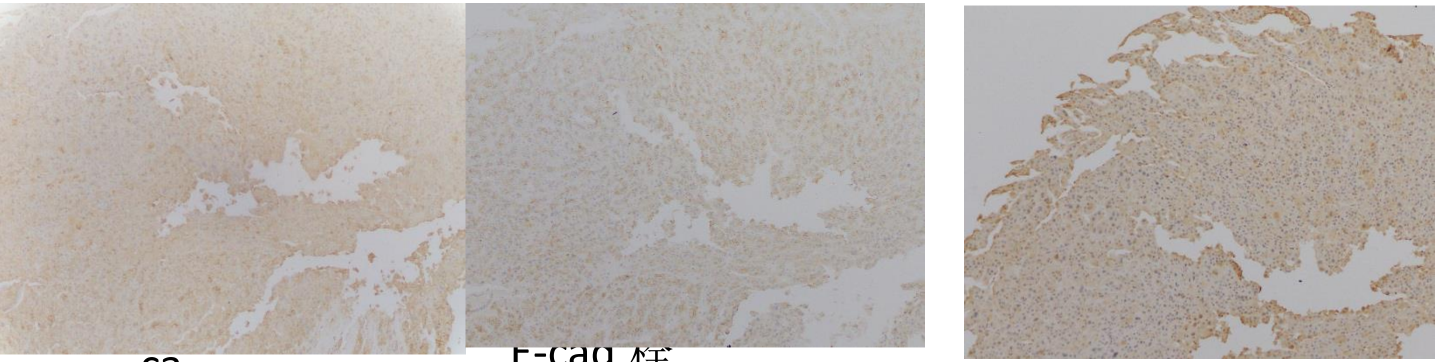

2013199 ca E-cad 柱

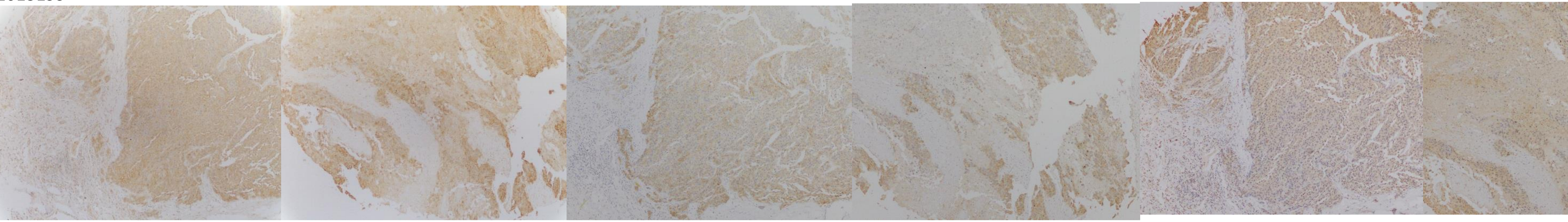

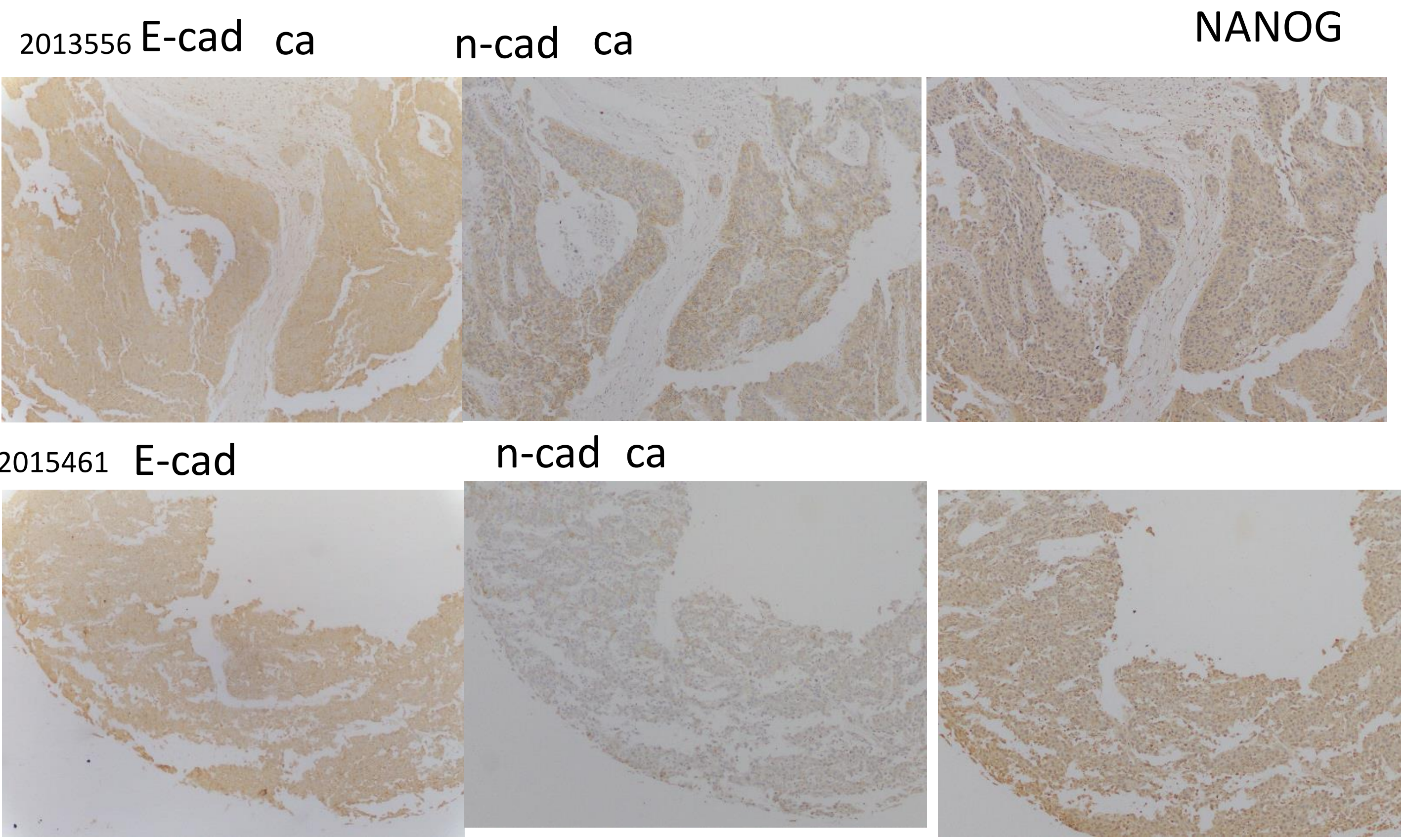
2013556 E-cad ca
n-cad ca
NANOG
2015461 E-cad
n-cad ca
