# Supplementary material for: Association of Preoperative NANOG-Positive Circulating Tumor Cell Levels With Recurrence of Hepatocellular Carcinoma
Source: Front Oncol. 2021 May 27;11:601668. doi: 10.3389/fonc.2021.601668 (PMC8190394; doi:10.3389/fonc.2021.601668)
Supplement: Supplementary file 2 [file DataSheet_2.docx]

Supplementary Material

# Supplementary Figures and Tables

Table S1 Patient characteristics

| Variable | N | % |
| --- | --- | --- |
| Gender(Male/Female) | 141/19 | 88.1/11.9 |
| Age(≤60/>60 years) | 123/37 | 76.6/23.1 |
| Tumor Size(≤5/>5cm) | 97/63 | 60.6/39.4 |
| Tumor Size (≤3/>3cm) | 64/96 | 40/60 |
| Tumor Number (≤1/>1) | 122/38 | 76.3/23.8 |
| Differentiation(H/M/L) | 23/103/34 | 14.4/64.4/21.3 |
| BCLC Stage(A/B+C) | 93/67 | 58.1/41.9 |
| Metastasis(Yes/No) | 21/139 | 13.1/86.9 |
| AFP (≤400/>400μg/L) | 104/56 | 65/35 |
| PIVKA(≤400/>400μg/L) | 90/70 | 56.2/43.8 |
| CTC (+/-) | 144/16 | 90/10 |
| CTC(≤13.3/>13.3) | 113/47 | 70.6/29.4 |
| Nanog(≤6.7/>6.7) | 105/55 | 65.6/34.4 |
| PIVKA(≤40/>40μg/L) | 43/117 | 26.9/73.1 |
| Vascular Invasion (+/-) | 59/101 | 36.9/63.1 |
| MVI(Yes/No) | 49/111 | 30.6/69.4 |
| MVD(Yes/No) | 28/132 | 17.5/82.5 |
| HBsAg(+/-) | 143/17 | 89.4/10.6 |
| Direct Bilirubin(≤4/>4) | 70/90 | 43.7/56.3 |
| Total Bilirubin (≤10/>10) | 13/147 | 8.1/91.9 |
| ALT(≤84/>84IU/L) | 134/26 | 83.8/16.3 |
| TNM(I-II/III-IV) | 113/47 | 70.6/29.4 |
| Edmondson Stage(I-II/III-IV) | 108/52 | 67.5/32.5 |
| HBVDNA(100≤/<100) | 89/71 | 55.6/44.4 |
| NLR(≤1.77/>1.77) | 38/122 | 23.8/76.3 |
| INR(≤1/>1) | 57/103 | 35.6/64.4 |
| Alcohol (Yes/No) | 71/89 | 44.4/55.6 |
| APRI(≤1/>1) | 144/16 | 90/10 |
| HB((≤120/>120)) | 17/143 | 10.6/89.4 |
| Neutrophil(≤4/>4) | 115/45 | 71.9/28.1 |
| Lymphocyte(≤1/>1) | 44/116 | 27.5/72.5 |
| PT(≤12/>12) | 86/74 | 53.8/46.3 |
| PLT(≤100/>100) | 45/115 | 28.1/71.9 |
| AST (≤45/>45) | 94/66 | 58.7/41.3 |
| ALB(≤35/>35) | 14/146 | 8.8/91.2 |
| ICG(≤10/>10%) | 136/24 | 85/15 |
| Recurrence(Yes/No) | 81/79 | 50.6/49.4 |
| Liver Cirrhosis (Yes/No) | 160/0 | 100/0 |

**Table S2 Primer sequences**

| **Gene** | **Primers sequences** |
| --- | --- |
| **EpCAM** | TGGTGCTCGTTGATGAGTCA AGCCAGCTTTGAGCAAATGA  AAAGCCCATCATTGTTCTGG CTCTCATCGCAGTCAGGATC  TCCTTGTCTGTTCTTCTGAC CTCAGAGCAGGTTATTTCAG |
| **CK8** | CGTACCTTGTCTATGAAGGA ACTTGGTCTCCAGCATCTTG  CCTAAGGTTGTTGATGTAGC CTGAGGAAGTTGATCTCGTC  CAGATGTGTCCGAGATCTGG TGACCTCAGCAATGATGCTG |
| **CK18** | AGAAAGGACAGGACTCAGGC GAGTGGTGAAGCTCATGCTG  TCAGGTCCTCGATGATCTTG CAATCTGCAGAACGATGCGG  AAGTCATCAGCAGCAAGACG CTGCAGTCGTGTGATATTGG |
| **CK19** | CTGTAGGAAGTCATGGCGAG AAGTCATCTGCAGCCAGACG  CTGTTCCGTCTCAAACTTGG TTCTTCTTCAGGTAGGCCAG  CTCAGCGTACTGATTTCCTC GTGAACCAGGCTTCAGCATC |
| **Vimentin** | GAGCGAGAGTGGCAGAGGAC CTTTGTCGTTGGTTAGCTGG  CATATTGCTGACGTACGTCA GAGCGCCCCTAAGTTTTTAA  AAGATTGCAGGGTGTTTTCG GGCCAATAGTGTCTTGGTAG |
| **Twist** | ACAATGACATCTAGGTCTCC CTGGTAGAGGAAGTCGATGT  CAACTGTTCAGACTTCTATC CCTCTTGAGAATGCATGCAT  TTTCAGTGGCTGATTGGCAC TTACCATGGGTCCTCAATAA |
| **CD45** | TCGCAATTCTTATGCGACTC TGTCATGGAGACAGTCATGT  GTATTTCCAGCTTCAACTTC CCATCAATATAGCTGGCATT  TTGTGCAGCAATGTATTTCC TACTTGAACCATCAGGCATC |
| **Nanog** | TGAGGCATCTCAGCAGAAGA TGTCCGAATAAGCAGATCC  GGGACTGGTGGAAGAATCAG GACACTCTTCTCTGCAGAAG  TTCTGTTTCTTGACCGGGAC GGAAGAGAACACAGTTCTGG  CATTGAGTACACACAGCTGG TTGGAGAGTTCTTGCATCTG |

**TableS3 Association of CTC counts and subtypes with early clinical characteristics**

| **Clinical characteristics** | **N** | **CTC totalcell** | | **E CTC** | | **Hybrid type** | | **M CTC** | |
| --- | --- | --- | --- | --- | --- | --- | --- | --- | --- |
|  |  | **P** | **r** | **p** | **r** | **p** | **r** | **p** | **r** |
| Gender(Male/Female) | 141/19 | 0.57 | -0 | 0.38 | 0.07 | 0.63 | -0.04 | 0.63 | -0.04 |
| Age(≤60/>60 years) | 123/37 | 0.77 | -0.2 | 0.88 | 0.01 | 0.29 | -0.08 | 0.29 | -0.08 |
| Tumor Size(≤5/>5cm) | 97/63 | 0.19 | 0.11 | 0.46 | 0.06 | 0.17 | 0.11 | 0.17 | 0.11 |
| Tumor Size(≤3/>3cm) | 64/96 | 0.26 | 0.09 | 0.41 | -0.1 | **0.02** | 0.187 | **0.02** | 0.179 |
| Tumor Number (≤1/>1) | 122/38 | 0.95 | 0.01 | 0.52 | 0.05 | 0.78 | 0.022 | 0.78 | 0.022 |
| Differentiation(H/M/L) | 23/103/34 | 0.94 | -0 | 0.89 | -0 | 0.92 | -0.01 | 0.79 | 0.021 |
| BCLC Stage(A/B+C) | 93/67 | **0.00** | **0.24** | 0.67 | 0.03 | **0.00** | **0.24** | **0.03** | **0.171** |
| Metastasis(Yes/No) | 21/139 | 0.51 | 0.05 | 0.38 | -0.1 | 0.27 | 0.089 | 0.61 | -0.04 |
| AFP (≤400/>400μg/L) | 104/56 | 0.64 | -0 | 0.56 | 0.05 | 0.80 | -0.02 | 0.57 | -0.05 |
| PIVKA(≤400/>400μg/L) | 90/70 | 0.48 | 0.06 | 0.45 | 0.06 | 0.51 | 0.053 | **0.02** | 0.18 |
| PIVKA(≤40/>40μg/L) | 43/117 | 0.56 | 0.05 | 0.18 | -0.1 | 0.73 | 0.028 | 0.90 | -0.01 |
| Vascular Invasion (+/-) | 59/101 | 0.14 | 0.12 | 0.52 | 0.05 | 0.27 | 0.089 | 0.14 | 0.118 |
| MVI(Yes/No) | 101/49 | 0.15 | 0.12 | 0.49 | 0.06 | 0.21 | 0.099 | 0.27 | 0.088 |
| MVD(Yes/No) | 132/28 | 0.87 | -0 | 0.85 | 0.02 | 0.78 | -0.02 | 0.27 | 0.088 |
| HBsAg(+/-) | 143/17 | 0.43 | 0.06 | 0.66 | 0.04 | 0.49 | 0.056 | 0.66 | -0.04 |
| Direct Bilirubin(≤4/>4) | 70/90 | 0.51 | 0.05 | 0.43 | -0.1 | **0.02** | 0.182 | 0.36 | 0.073 |
| Total Bilirubin (≤18/>18) | 13/147 | 0.10 | 0.13 | 0.17 | -0.1 | 0.35 | 0.075 | 0.72 | 0.029 |
| ALT(≤84/>84IU/L) | 134/26 | 0.07 | 0.14 | 0.59 | 0.04 | **0.04** | 0.161 | 0.07 | 0.144 |
| TNM(I-II/III-IV) | 113/47 | 0.77 | 0.02 | 0.94 | 0.01 | 0.62 | -0.04 | 0.64 | 0.037 |
| EdmondsonStage(H/M/L) | 23/103/34 | 0.86 | 0.01 | 0.7 | 0.03 | 0.92 | -0.01 | 0.79 | 0.021 |
| HBVDNA(≤100/>100) | 89/71 | **0.04** | 0.16 | 0.06 | 0.15 | 0.06 | 0.15 | **0.004** | 0.227 |
| NLR(≤1.77/>1.77) | 38/122 | 0.59 | -0.4 | 0.58 | 0.05 | 0.25 | -0.09 | 0.35 | -0.07 |
| INR(≤1/>1) | 57/103 | 0.33 | 0.08 | 0.09 | -0.1 | 0.13 | 0.122 | 0.85 | 0.015 |
| Alcohol (Yes/No) | 89/71 | 0.28 | 0.09 | 0.92 | -0 | 0.41 | 0.066 | 0.38 | 0.07 |
| APRI(≤1/>1) | 144/16 | 0.78 | 0.02 | 0.89 | 0.01 | 0.55 | 0.047 | 0.64 | 0.037 |
| HB((≤120/>120)) | 13/143 | 0.89 | 0.01 | 0.06 | -0.1 | 0.84 | 0.016 | 0.42 | -0.07 |
| Neutrophil(≤2/>2) | 115/45 | 0.20 | 0.1 | 0.17 | 0.11 | 0.16 | 0.112 | 0.23 | 0.095 |
| Lymphocyte(≤1/>1) | 44/116 | 0.51 | 0.05 | 0.91 | 0.01 | 0.63 | 0.038 | 0.42 | 0.065 |
| PT(≤12/>12) | 86/74 | 0.28 | 0.09 | 0.23 | -0.1 | 0.17 | 0.109 | 0.82 | -0.02 |
| PLT(≤100/>100) | 45/115 | 0.94 | 0.01 | 0.07 | 0.14 | 0.13 | 0.122 | 0.39 | 0.069 |
| WBC(≤10/>10) | 158/2 | **0.03** | 0.18 | 0.28 | 0.09 | **0.02** | 0.182 | 0.72 | -0.03 |
| AST (≤45/>45) | 94/66 | 0.32 | 0.08 | 0.49 | 0.06 | 0.34 | 0.075 | **0.02** | 0.186 |
| ALB(≤35/>35) | 14/146 | **0.04** | -0.2 | 0.09 | -0.1 | 0.95 | 0.005 | **0.01** | -0.2 |
| ICG(≤10/>10%) | 136/24 | 0.99 | 0 | 0.92 | 0.01 | 0.22 | -0.1 | 0.39 | -0.07 |

p value is calculated by chi-square test or Pearson's chi-squared test

.

**Table S4 Association of clinical characteristics and pathological factors with** **early recurrence**

| **Clinical characteristics** | **Early recurrence** | |  |
| --- | --- | --- | --- |
|  | No | Yes | P |
| Gender(Male/Female) | 71/8 | 70/11 | 0.5 |
| Age(≤60/>60years) | 63/16 | 60/21 | 0.395 |
| Tumor Size(≤5/>5cm) | 52/27 | 45/36 | 0.184 |
| Tumor Size(≤3/>3cm) | 34/45 | 30/51 | 0.439 |
| Tumor Number (≤1/>1) | 63/16 | 59/22 | 0.305 |
| Differentiation(H/M/L) | 11/52/16 | 12/51/18 | 0.93 |
| BCLC Stage(A/B+C) | 50/29 | 43/38 | 0.191 |
| Metastasis (Yes/NO) | 68/11 | 71/10 | 0.768 |
| AFP (≤400/>400μg/L) | 57/22 | 47/34 | 0.061 |
| PIVKA(≤400/>400μg/L) | 46/33 | 44/37 | 0.618 |
| CTC(+/-) | 70/9 | 74/7 | 0.562 |
| CTC(≤13.3/>13.3) | 57/22 | 56/25 | 0.675 |
| Nanog≤6.7/>6.7 | 62/17 | 43/38 | **0.001** |
| PIVKA(≤40/>40μg/L) | 23/56 | 20/61 | 0.528 |
| Vascular Invasion (+/-) | 31/48 | 28/53 | 0.54 |
| MVI(Yes/No) | 26/53 | 23/58 | 0.535 |
| MVD(Yes/No) | 11/68 | 17/64 | 0.24 |
| HBsAg(+/-) | 69/10 | 74/7 | 0.41 |
| Direct Bilirubin(≤4/>4) | 35/44 | 35/46 | 0.889 |
| Total Bilirubin (≤10/>10 | 7/72 | 6/75 | 0.737 |
| ALT(≤84/>84IU/L) | 65/14 | 69/12 | 0.618 |
| TNM(I-II/III-IV) | 58/21 | 55/26 | 0.444 |
| Edmondson Stage(I-II/III-IV) | 55/24 | 53/28 | 0.572 |
| HBV DNA(≤100/>100) | 46/33 | 43/38 | 0.513 |
| NLR(≤1.77/>1.77) | 18/61 | 20/61 | 0.777 |
| INR(≤1/>1) | 34/45 | 23/58 | 0.053 |
| ALCHOL(Yes/No) | 43/36 | 46/35 | 0.764 |
| APRI(≤1/>1) | 70/9 | 74/7 | 0.562 |
| HB(≤120/>120) | 9/70 | 8/73 | 0.756 |
| Neutrophil(≤4/>4) | 54/25 | 61/20 | 0.328 |
| Lymphocyte(≤1/>1) | 20/59 | 24/57 | 0.541 |
| PT(≤12/>12) | 48/31 | 38/43 | 0.079 |
| PLT(≤100/>100) | 21/58 | 24/57 | 0.668 |
| AST (≤45/>45) | 42/37 | 52/29 | 0.156 |
| ALB(≤35/>35) | 5/74 | 9/72 | 0.285 |
| ICG(≤10/>10%) | 68/11 | 68/13 | 0.707 |

p value is calculated by chi-square test or Pearson's chi-squared test

Table S5 Correlation between expression of nanog and clinic opathology in 126 patients with HCC

| **Clinical characteristics** | **NANOG** | |  |
| --- | --- | --- | --- |
|  | **Low level** | **High level** | **p-value** |
| Gender:(Male/Female) | 8/58 | 3/57 | 0.157 |
| Age:(≤50 / >50 years) | 42/24 | 42/18 | 0.449 |
| Tumor size:(≤5 />5 cm) | 26/40 | 16/44 | 0.13 |
| Tumor size:(≤3 />3 cm) | 11/55 | 5/55 | 0.161 |
| Differentiation: (H/M/L) | 5/55/6 | 3/39/18 | **0.011** |
| BCLC stage:(A/B+C) | 39/27 | 18/42 | **0.001** |
| Metastasis: (-/+) | 49/17 | 28/32 | **0.002** |
| Vascularthrombus:(-/+) | 49/17 | 33/27 | **0.024** |
| Recurrence:(-/+) | 25/41 | 9/51 | **0.004** |
| Survival:(-/+) | 33/33 | 46/14 | **0.002** |
| Lymphatic metastasis:(-/+) | 64/2 | 55/5 | 0.194 |
| TNM:(I-II/III-VI) | 28/38 | 14/46 | **0.023** |

P value of < 0.05 was considered statistically-significant Pearson Correlation Coefficien

Table S6 The correlation between different ages and the expression of nanog

|  | **Young** | **Middle age** | **old** |
| --- | --- | --- | --- |
| **Nanog Low** | **5(35.7%)a** | **33(66.0%)a** | **67(69.8%)a** |
| **Nanog High** | **9(64.3%)b** | **17(34.0%)a** | **29(30.2%)a** |
| **total** | **14** | **50** | **96** |

The table marked the results of the pairwise comparison by subscripts (a, b, c, etc.). If the marked letters between the two groups are the same, it means that the difference between the two groups is not statistically significant; if the two marked letters are different, it means The difference between the two groups was statistically significant.

## Supplementary Figures


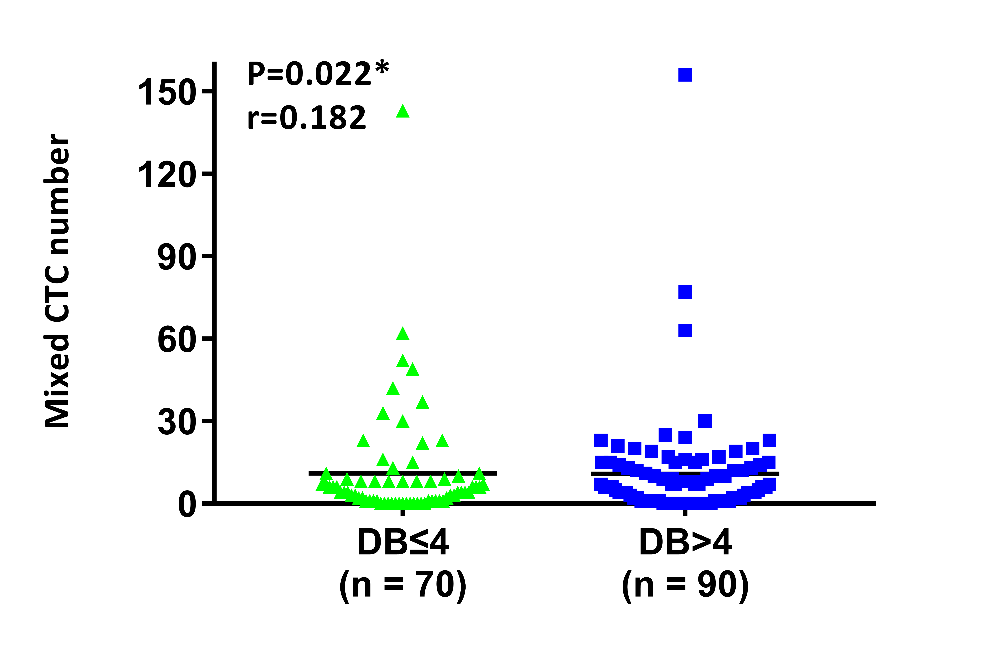


**Supplementary Figure 1.** Correlations of the expression level of mixed CTC number with Direct bilirubin(DB).


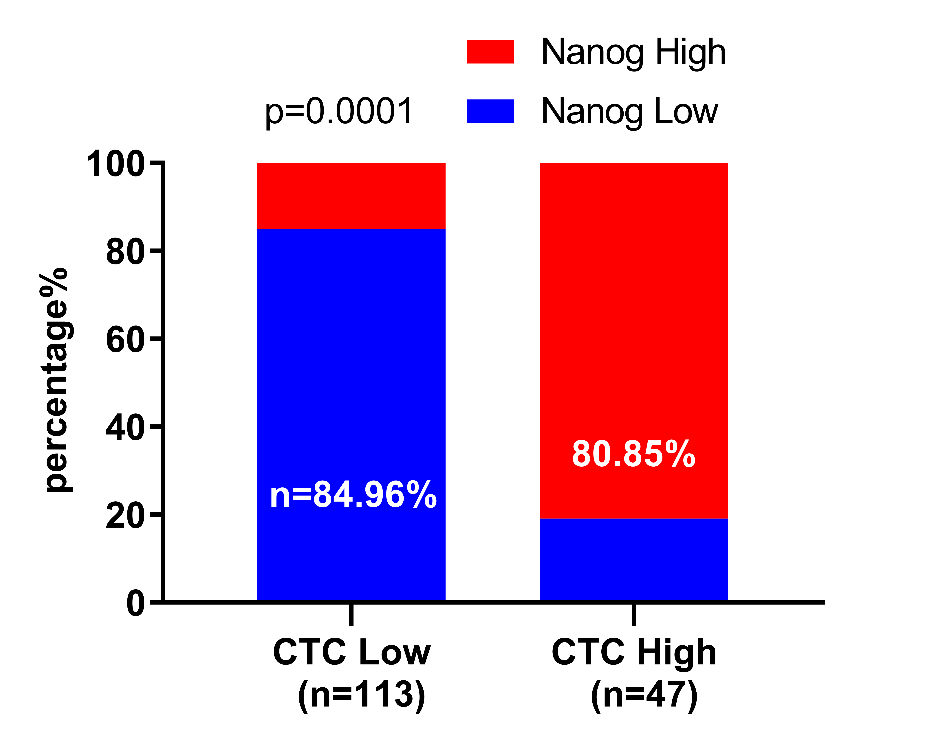


**Supplementary Figure 2.** The correlation between the number of CTC and the number of NANOG positive cells in peripheral blood.
